# Epidemiological and Clinical Features of *Streptococcus dysgalactiae* ssp. *equisimilis stG62647* and Other *emm* Types in Germany

**DOI:** 10.3390/pathogens12040589

**Published:** 2023-04-13

**Authors:** Andreas Itzek, Victoria Weißbach, David Meintrup, Beate Rieß, Mark van der Linden, Stefan Borgmann

**Affiliations:** 1 German National Reference Center for Streptococci, Institute of Medical Microbiology, University Hospital RWTH Aachen, 52074 Aachen, Germany; 2 Department of Infectious Diseases and Infection Control, Ingolstadt Hospital, 85049 Ingolstadt, Germany; 3 Faculty of Engineering and Management, University of Applied Sciences Ingolstadt, 85049 Ingolstadt, Germany

**Keywords:** *Streptococcus dysgalactiae* subspecies *equisimilis*, SDSE, Germany, *stG62647*, *emm* type, surveillance, clinical study

## Abstract

(1) Background: *Streptococcus dysgalactiae* subspecies *equisimilis* (SDSE) is an important β-hemolytic pathogen historically described as mainly affecting animals. Studies epidemiologically assessing the pathogenicity in the human population in Germany are rare. (2) Methods: the present study combines national surveillance data from 2010 to 2022 with a single-center clinical study conducted from 2016 to 2022, focusing on *emm* type, Lancefield antigen, antimicrobial resistance, patient characteristics, disease severity, and clinical infection markers. (3) Results: The nationwide reported invasive SDSE infections suggest an increasing infection burden for the German population. One particular *emm* type, *stG62647*, increased over the study period, being the dominant type in both study cohorts, suggesting a mutation-driven outbreak of a virulent clone. The patient data show that men were more affected than women, although in the single-center cohort, this trend was reversed for patients with *stG62647* SDSE. Men affected by *stG62647* developed predominantly fascial infections, whereas women suffering from superficial and fascial non-stG62647 SDSE infections were significantly younger than other patients. Increasing age was a general risk factor for invasive SDSE infections. (4) Conclusions: further studies are needed to further elucidate the raised questions regarding outbreak origin, underlying molecular mechanisms as well as sex-dependent pathogen adaptation.

## 1. Introduction

*Streptococcus dysgalactiae* subspecies *equisimilis* (SDSE) is an important β-hemolytic pathogen mainly expressing Lancefield antigens C or G, causing severe infections in humans [1]. The taxonomic and pathogenic classification of the bacterial species has undergone several changes [2,3,4], but the historic description as mainly animal pathogenic, rarely associated with zoonotic infections [5] is highly questionable [6,7]. This volatility might be a reason why its impact on human health is still underestimated by public health institutions [8], even though SDSE resembles the pathogenic potential of the major β-hemolytic human pathogen *S. pyogenes* able to cause severe tissue infections [9,10], sepsis [11,12], toxic shock syndrome [13,14], and in rare cases meningitis [11,15]. Furthermore, the incidence of human *S. dysgalactiae* infections is constantly increasing worldwide [16,17,18,19,20], in some geographical regions even overtaking *S. pyogenes* [21,22,23].

A potential explanation for the success of this pathogen might be its virulence factor arsenal [24], the majority of which is already well described for *S. pyogenes* [25], including the major virulence mediator, the M protein [26,27]. This filamentous protein, encoded by the *emm* gene (**E**ncoding **M**ature **M** protein) is presented in a coiled-coil dimeric structure on the bacterial surface [28], covalently linked to the Gram-positive cell wall by the LPXTG mechanism [29]. Expressed in very high amounts, the M protein covers the bacterial cell like a protective fur [30], interacting with different host molecules [31,32,33] and in that way inhibiting phagocytosis [34,35] and mediating interaction with different cell types [36,37,38,39]. The N-terminal region of the M protein shows high variability in amino acid composition, facilitating antigenic diversity [40], that on one hand might be the reason for recurrent infections with the same bacterial species [41,42], and on the other hand, allows epidemiological tracing by nucleotide sequence-based *emm* typing [43,44].

To date, more than one hundred different *emm* types have been described for SDSE [45], of which only a small subset is frequently isolated in Germany [46,47]. Significant differences in the *emm* type distribution have been described for various geographical regions [18,19,20,48], but only a few studies discovered the long-term *emm* type shift toward one obviously very successful type, denoted *stG62647*. First mentioned in a multicenter study from Argentina in 2016, this *emm* type was already described as the most abundant in the analyzed patient cohort [48]. In 2017, a retrospective study confirmed the dominance of *stG62647* also for invasive SDSE infections in Switzerland [49]. In the same year, an increase in *stG62647* infections since 2013 was also described in Norway, combined with a comprehensive genome analysis approach to examine the molecular basis for the success of this particular *emm* type [50]. Interestingly, Oppegaard et al. [50] showed that the vast majority of *stG62647* isolates from invasive infections in Bergen contained a genetic disruption in the streptococcal invasive locus (*sil*), induced by the insertion of a transposase into the *silB*-gene, potentially interfering with virulence factor regulation, offering a possible explanation for the fulminant disease development observed. Since then, the dominance of *stG62647* in invasive but also non-invasive SDSE infections has been confirmed by different single- and multicenter studies in Germany [46,47], Sweden [51,52], Denmark [52], and Spain [53]. However, further research, explaining the evolutionary and pathogenic success of this particular *emm* type, is still lacking.

The present work combines national surveillance data with a single-center clinical study to highlight recent developments in the *emm* type distribution of invasive SDSE infections in Germany with a special focus on the rise of the very successful type *stG62647*.

## 2. Materials and Methods

### 2.1. Sample Collection

The German National Reference Center for Streptococci (GNRCS) has performed surveillance of invasive streptococcal infections in Germany since 1996. Primary microbiological laboratories are asked to send streptococcal isolates of invasive infections on a voluntary basis. The collection of corresponding patient data is addressed by a standardized questionnaire, to be filled out by the participating centers. The requested information includes primary bacterial species identification, isolation material, date of isolation, patient date of birth, patient sex, patient residence, diagnosis, and underlying diseases.

### 2.2. Data Preparation

All bacterial isolates sampled between 1 January 2010 and 31 December 2022, identified as SDSE were included in the study. Exclusion criteria were patient national extraction (patients without regular residence in Germany), non-invasive infection (isolate not sampled from a normally sterile anatomical site, sampling information missing, or SDSE isolate not identified as the primary source of infection), and potential clonality (isolates from the same patient with identical *emm* type/subtype and antibiotic sensitivity pattern).

Sample material data were consolidated into seven categories, including blood isolates, tissue isolates (tissue, biopsy, aortic valve, mitral valve, heart valve, calf muscle, knee tissue, and muscle tissue), puncture isolates (puncture, joint puncture, and knee joint puncture), swab isolates (swab and Achilles’ tendon swab), cerebrospinal fluid isolates, urine isolates, and isolates from other sources (prosthesis material, fragment knee, implant material, and secretion).

### 2.3. Microbiological Analyses

All bacterial isolates received at the GNRCS were cultivated on Tryptone Soya agar with sheep blood (Thermo Scientific™, Schwerte, Germany) for 18 h at 37 °C and 5% CO_2_. The species identification was performed by a combination of hemolysis and colony size assessment, catalase, leucine aminopeptidase [54], and pyrrolidonylarylamidase tests [55] as well as Lancefield antigen typing (Prolex™ Streptococcal Grouping Kit, Richmond Hill, ON, Canada; ImmuLex Streptococcus Group L, SSI-Diagnostica, Hillerød, Denmark), and in some cases MALDI-Biotyper^®^ analysis and/or *16S rRNA* gene sequencing (primers: 27F and 1492R). For SDSE isolates, *emm* typing was performed in accordance with the CDC guidelines for *S. pyogenes* using two different primer sets [56,57] and strepBLAST 2.0 server. The current *emm* nomenclature denotes the gene symbol (e.g., *stG*) followed by a number specifying the main type (e.g., *stG62647*), while the subtype, characterized by single mutation variants of the main type, is given after a dot (e.g., *stG62647.0*).

### 2.4. Antibiotic Sensitivity Pattern

Susceptibility testing for nine antimicrobial substances (penicillin, amoxicillin, cefotaxime, vancomycin, erythromycin/clarithromycin, clindamycin, chloramphenicol, tetracycline, and levofloxacin) was performed by broth microdilution (Sensititre™ NLMCP2, Thermo Scientific™, East Grinstead, UK) following the guidelines M100 (Performance Standards for Antimicrobial Susceptibility Testing) and M07 (Methods for Dilution Antimicrobial Susceptibility Tests for Bacteria That Grow Aerobically) of the Clinical and Laboratory Standards Institute (CLSI).

### 2.5. Single Center Microbiological Analyses

Ingolstadt Hospital is a secondary 1155-bed hospital in the center of Bavaria (southeastern Germany), providing health care for about 500,000 inhabitants in the planning region 10. The hospital boasts three intensive care units and intermediate care as well as a stroke unit. The hospital has four internal and various surgical facilities. At the microbiological laboratory of Ingolstadt Hospital, SDSE isolates were cultured in thioglycolate broth, on sheep blood agar (bioMérieux, Nürtingen, Germany), Schaedler agar containing 5% sheep blood (bioMérieux), and/or chocolate agar (bioMérieux). Urine samples were cultured on sheep blood agar covered with a 50 µg pipemidic acid disk (BioRad, Marnes-la-Coquette, France) to inhibit the growth of Gram-negative bacteria. Blood cultures were grown in aerobe and anaerobe blood culture bottles using the BactAlert blood culture system (bioMérieux). After bacterial growth was detected by the BactAlert system, blood samples from aerobe bottles were given on blood and chocolate agar and additionally on Schaedler agar in the case of bacterial growth in anaerobe blood culture bottles. Species identification was either determined using Vitek 2 MS (bioMérieux) or the Vitek 2 compact (bioMérieux) with appropriate Vitek 2 identification (ID) cards.

### 2.6. Clinical Chemistry

Laboratory values (concentration of leukocytes, C-reactive protein (CRP), lactate dehydrogenase (LDH), creatine phosphokinase (CPK), and aspartate aminotransferase (AST)) were regarded as surrogate markers for inflammation and tissue destruction. The values of blood samples obtained from the day of bacterial sampling were observed. If no blood had been taken on the day of bacterial sampling, data from samples obtained on the preceding or the following days were used, a maximum of three days before or after bacterial sampling. The concentrations of CRP, LDH, CPK, and AST were determined using the Alinity analyzer (Abbott, Abbott Park, IL, USA) whereas the concentration of leukocytes was measured with the UniCel DxC 600 Chemistry Analyzer (Beckman Coulter Inc., Brea, CA, USA). The laboratory of Ingolstadt Hospital is certified in accordance with the national accreditation body of the Federal Republic of Germany (Deutsche Akkreditierungsstelle; DAkkS; registration number D-ML-19856-01-00).

### 2.7. Clinical Cohort Description

Ingolstadt Hospital sent 123 SDSE isolates causing colonization, as well as peripheral and invasive infections from December 2016 to November 2022 to the GNRCS for *emm* and Lancefield antigen typing. From this single-center study cohort, 19 cases were excluded due to the lack of patients’ informed consent. The remaining 104 SDSE cases were classified into 82 invasive and 22 non-invasive infections (isolate not sampled from a normally sterile anatomical site or SDSE isolate not identified as the primary source of infection). Infections were further categorized into three groups for assessing the impact of particular *emm* types on the clinical course. Colonization of the skin and urinary tract, as well as infections restricted to the skin and mucous membranes, were categorized as superficial infections. Infections of soft tissue and organs including urinary tract infections with a significant concentration of bacteria (>100,000 cfu/µL) were regarded as fascial infections. Isolation of SDSE from blood culture and infections leading to systemic affections, e.g., the need for catecholamine treatment, were categorized as systemic infections. The category “Sampling (days after admission)” was defined as the time period from patient admission to the day of bacterial sampling, finally resulting in growth-dependent detection of SDSE. If SDSE had been isolated from a sample taken at the pre-hospital appointment, the number of days until sampling resulted in a negative value (patient 102). The length of hospital stay was calculated in days, as the time period from patient admission until discharge.

### 2.8. Statistical Analysis

Descriptive statistics for categorical variables are displayed as absolute or relative frequencies. For continuous variables, mean, median, and standard deviations are shown. Hypothesis tests for proportions were performed using a chi-square test. Mean values were compared using a *t*-test. In both cases, small *p*-values were interpreted as an indication in favor of the alternative hypothesis.

## 3. Results

### 3.1. Invasive SDSE Infections in Germany

**Figure 1:**
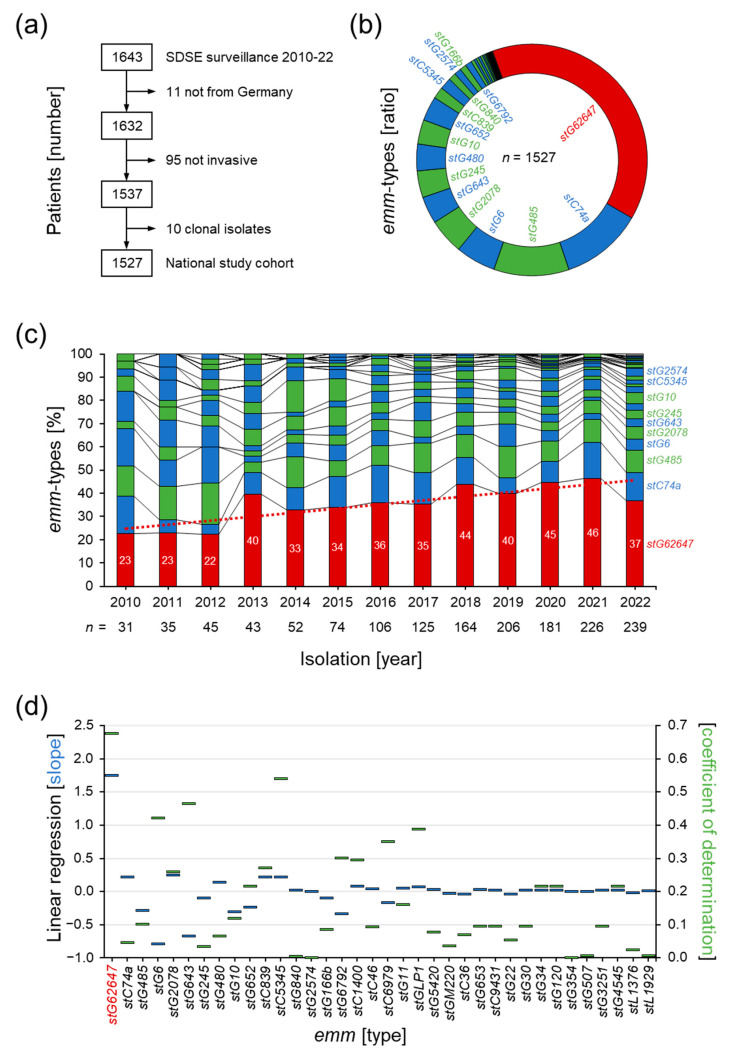
Surveillance of invasive SDSE infections in Germany in the years 2010 to 2022 by the German National Reference Center for Streptococci. (**a**) Summary of the performed study cohort validation. (**b**) Distribution of *emm* types in the national study cohort. (**c**) Yearly changes in the *emm* type composition of the national study cohort (columns) with linear regression of the *stG62647* isolate rate (dotted line). (**d**) Slope (blue bars) and coefficient of determination (green bars) of the linear regression of the chronological changes in *emm* type composition in the national study cohort.

In the study period from 2010 to 2022, the GNRCS received 1643 SDSE isolates associated with severe human infections. From this collection, 116 isolates were excluded due to not fulfilling the inclusion criteria for a national study cohort of invasive SDSE infections (Figure 1a). A total of 11 isolates were derived from patients without regular residence in Germany, 95 isolates were not sampled from normally sterile body sites, sampling information was missing, or SDSE could not be identified as the primary source of infection, and 10 isolates were identified as potential clones. The final study cohort included 1527 invasive SDSE isolates from German patients (Table S2).

The performed typing of the M protein gene (*emm*) revealed a dominance of one particular *emm* type denoted *stG62647* (Figure 1b). A total of 39% (*n* = 594) of all invasive SDSE isolates in the national study cohort belonged to this type, while all other *emm* types showed abundance values below 12%, with *stC74a* (11.5%, *n* = 174) and *stG485* (10.5%, *n* = 161) being the second and third most abundant types.

Chronological analysis showed a steady increase in invasive *stG62647* cases over the study period, starting from 23% in 2010, doubling to 46% in 2021, but decreasing to 37% in 2022 (Figure 1b). Detailed analysis of the *emm* type composition of each study year clearly showed that *stG62647* was the *emm* type with the highest linear increase, with a slope of 1.8 and a coefficient of determination of 0.67 (Figure 1c), including a sudden upsurge in 2013 to 40%. No other *emm* type showed a significant linear relationship during the study period, and all correlations found were spurious.

### 3.2. Dissection of Epidemiological Markers

**Figure 2:**
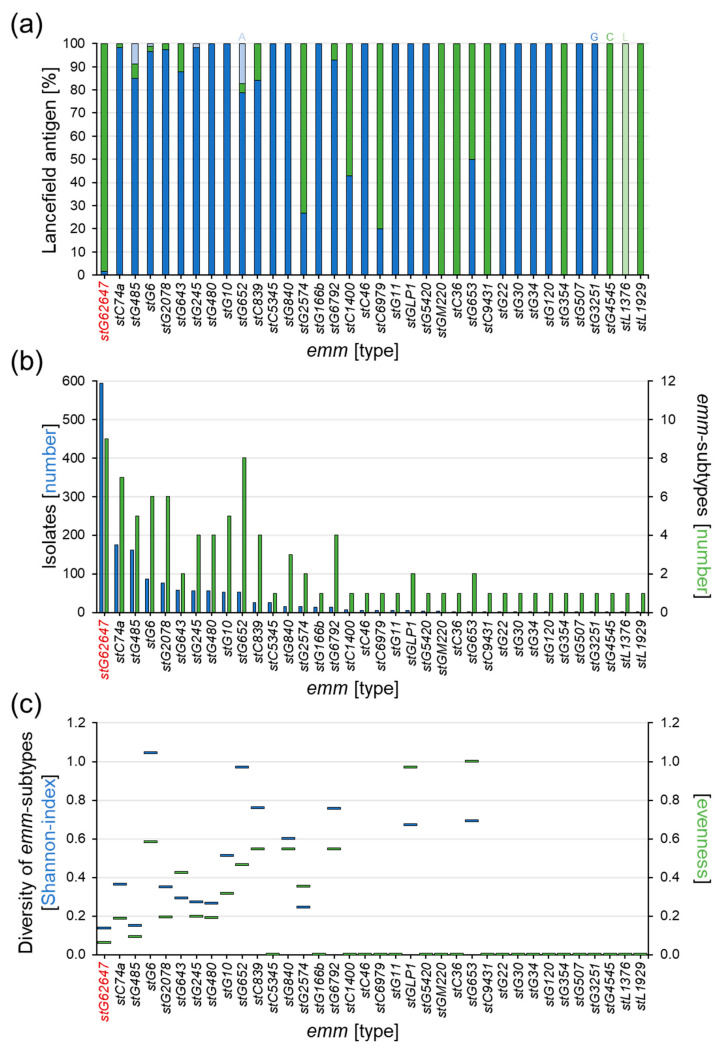
Analysis of the Lancefield antigen expression and mutation rate within the different *emm* types of the national study cohort. (**a**) Ratio of Lancefield antigen expression of the 36 *emm* types in the national study cohort (columns), showing antigen A (light blue), antigen C (green), antigen G (blue), and antigen L (light green). (**b**) Number of isolates per *emm* type (blue columns) and number of corresponding *emm* subtypes (green columns) for all 36 *emm* types in the national study cohort. (**c**) Comparison of relative mutation rate within the 36 *emm* types by Shannon index (blue bars) and evenness (green bars).

The determination of the Lancefield antigen repertoire showed that *stG62647*, in contrast to the majority of the other *emm* types in the national study cohort, mainly expresses the C antigen (98.3%, *n* = 584) and only 1.7% (*n* = 10) of the *stG62647* isolates were positive for the G antigen (Figure 2a). The Lancefield types A and L were not found in *stG62647*. Other *emm* types, mainly associated with Lancefield antigen C, such as *stC36*, *stC1400*, *stC6979*, *stC9431*, *stG354*, *stG2574*, *stGM220,* and *stL1929* are much less abundant in the study cohort, with 1 to 15 isolates in twelve years. Consequently, *stG62647* with 594 isolates is by far the most abundant Lancefield-C SDSE, associated with invasive infections in humans.

A distinct correlation between isolate numbers and the relative mutation rate of the different *emm* types was not observed in the study cohort. However, even though *stG62647* showed the highest number of *emm* subtypes in the cohort, the sample distribution among these nine different subtypes was extremely shifted toward *stG62647.0*, with 594 isolates (Figure 2b), leaving only twelve isolates for the remaining eight subtypes *stG62647.3* (*n* = 1), *stG62647.4* (*n* = 4), *stG62647.5* (*n* = 1), *stG62647.6* (*n* = 1), *stG62647.7* (*n* = 1), *stG62647.8* (*n* = 1), *stG62647.11* (*n* = 2), and *stg62647.13* (*n* = 1), which is clearly depicted by the resulting very low diversity indices (Figure 2c). With a Shannon index of 0.14 and an evenness of 0.06, *stG62647* has the lowest intra-*emm*-type diversity of all *emm* types associated with subtypes in the national study cohort, closely followed by *stG485* (H′ ≈ 0.15, J′ ≈ 0.09).

### 3.3. Comparison of stG62647 with Other emm Types

**Figure 3:**
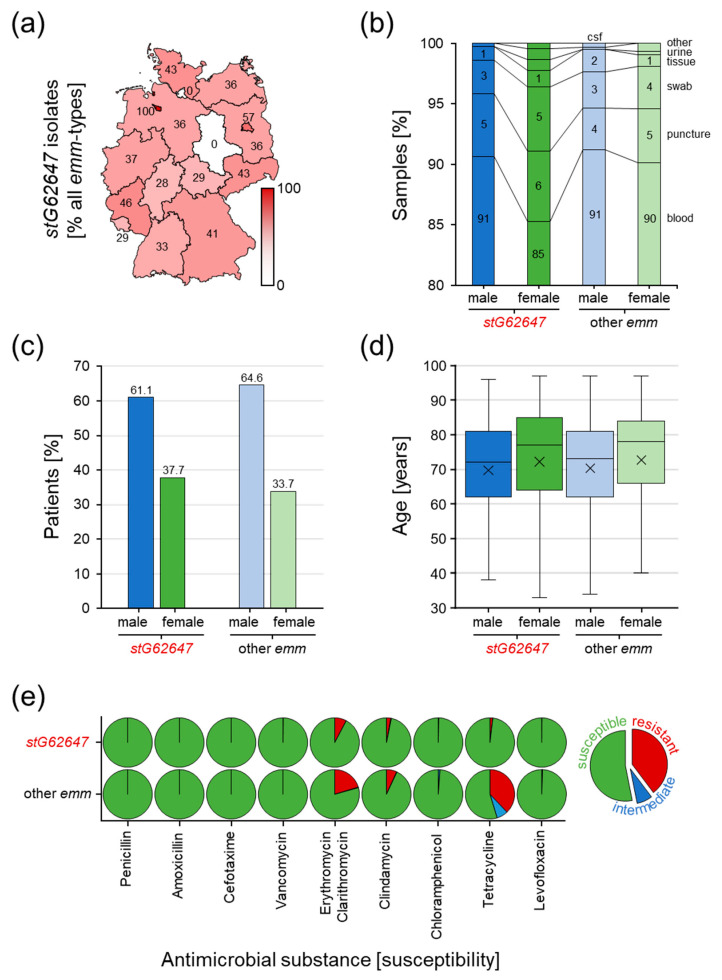
Analysis of potential differences between invasive SDSE infections associated with *stG62647* and other *emm* types in the national study cohort. (**a**) Geographical disaggregation of the ratio of *stG62647* cases against total invasive SDSE infections (red gradation and values in the map) by the German federal state of the corresponding patient residence. (**b**) Differences in sample material ratio between *stG62647* cases (dark colored columns) and other *emm* types (light colored columns), discriminating male (blue) and female (green) patients. (**c**) Comparison of invasive SDSE infections associated with *stG62647* (dark colored columns) and all other *emm* types (light colored columns) of the national study cohort discriminating male (blue) and female (green) patients. (**d**) Analysis of invasive infections associated with *stG62647* (dark colored boxes) and all other *emm* types (light colored boxes) of the national study cohort regarding age distribution for male (blue) and female (green) patients with the median (line) and arithmetic mean (cross). (**e**) Susceptibility of *stG62647* isolates and isolates of all other *emm* types of the national study cohort to nine antimicrobial substances, discriminating susceptible (green), intermediate (blue), and resistant (red) following the guidelines of the Clinical and Laboratory Standards Institute.

The geographical distribution showed no distinct accumulation of invasive SDSE infections caused by *stG62647* (Figure 3a). Very high ratios of cases associated with *stG62647* are the result of generally low numbers of invasive SDSE isolates, reported to the GNRCS in the corresponding federal state during the study period, as depicted for Bremen with 100% (*stG62647*/other = 1/0), Hamburg with 0% (*stG62647*/other = 0/2), and Saxony-Anhalt with 0% (*stG62647*/other = 0/2). The ratio of invasive *stG62647* infections versus other *emm* types in the remaining thirteen German federal states varied between 28% in Hesse (*stG62647*/other = 35/90) and 57% in Berlin (*stG62647*/other = 4/3).

The consolidation of the provided data regarding sample material clearly revealed that the vast majority of invasive SDSE isolates are sampled from blood cultures, with 90.6% for male and 85.3% for female *stG62647*-affected patients as well as 91.2% male and 90.1% female patients infected by other SDSE *emm* types (Figure 3b). The marginally lower amount of blood cultures observed for female *stG62647* patients is compensated by other isolation sources, mainly swab cultures. However, no significant differences in the sampling material distribution could be observed between the different *emm* types.

The consideration of patient sex showed that men in general are more affected by invasive SDSE infections than women (Figure 3c). However, this trend appears to be less pronounced for *stG62647*, with only 61.1% of patients being male, while all other *emm* types show a higher ratio, with 64.6% male patients.

The combination of patient data regarding sex and age at the time of infection revealed that women are in general five years older than men when affected by an invasive SDSE infection, with a median of 77.5 years compared to 72.5 years for men (Figure 3d). Significant differences in sex-dependent age distribution between cases associated with *stG62647* and other *emm* types could not be observed.

Determination of antimicrobial susceptibility showed differences between the compared *emm* types (Figure 3e). Isolates of the *stG62647 emm* type are more susceptible to five of the nine antimicrobial substances tested. While all analyzed SDSE isolates showed full susceptibility to penicillin, amoxicillin, cefotaxime, and vancomycin, resistance against erythromycin/clarithromycin (*stG62647*/other = 8%/20%), clindamycin (3%/7%), tetracycline (2%/38%), and to a minor extent levofloxacin (0.17%/0.32%) and chloramphenicol (0.00%/0.11%) was, in general, lower in *stG62647* isolates compared to all other *emm* types.

### 3.4. Description of the Clinical Cohort at Ingolstadt Hospital

At Ingolstadt Hospital, 82 invasive SDSE cases were examined, of which 36 were female and 46 male (Table 1). The age of the affected patients ranged from 27 to 93 years with a median of 65. None of the included patients died as a consequence of the identified SDSE infection. Most of the bacterial isolates were identified in the blood (29.3%), swab cultures (25.6%), and sore smear (23.2%), while the remaining samples were from other isolation sources (Table S1). Based on diagnostic data and sampling material, the cases were categorized into superficial (34.1%), fascial (31.7%), and systemic infections (34.1%). The average time span between patient admission and bacterial sampling was 3.7 days, while the average patient hospitalization time was 20.8 days. The Lancefield antigen distribution of the corresponding SDSE isolates was almost uniform, with antigen C at 48.8% and antigen G at 51.2%. Sequencing of the M protein gene revealed that 38 (46.3%) isolates belonged to the *emm* type *stG64247*, all expressing Lancefield antigen C (Table S1). The remaining 44 SDSE isolates belonged to 15 additional *emm* types comprising one to nine isolates, respectively.

### 3.5. Impact of Bacterial emm Type, Patient Sex, and Age on Clinical Courses

**Figure 4:**
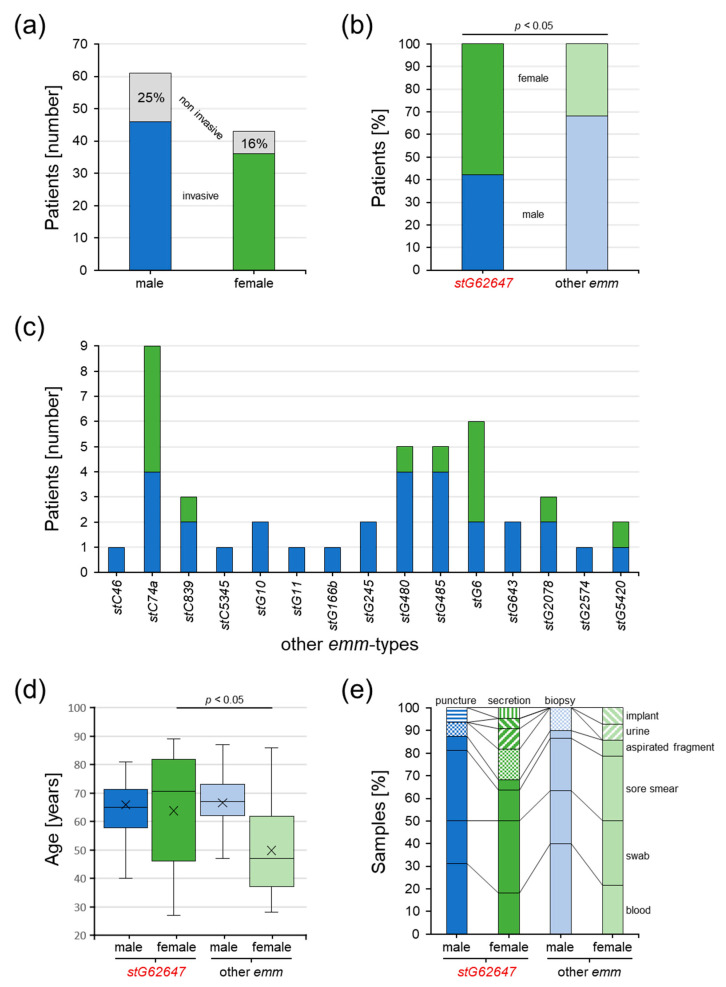
Characteristics of SDSE-infected patients of the single-center cohort from Ingolstadt Hospital separated into *stG62647* cases (dark colored) and cases associated with other *emm* types (bright colored) divided by male (blue) and female patients (green). (**a**) The number of invasive (colored bars) and excluded non-invasive SDSE cases (grey bars). (**b**) The ratio of invasive SDSE cases separated by patient sex and *emm* type; *p*-value was calculated by the chi-squared test. (**c**) Distribution of *emm* types other than *stG62647* in invasive SDSE cases, separated by patient sex. (**d**) Distribution of patient age separated by patient sex and *emm* type of invasive SDSE cases with indicated median (line) and arithmetic mean (cross); *p*-value originated from a contrast within a two-way ANOVA model. (**e**) Distribution of isolation material separated by patient sex and *emm* type of invasive SDSE cases.

At Ingolstadt Hospital, SDSE isolates of 104 patients were examined, of which 82 were classified as invasive (46 male, 36 female), whereas 22 were excluded from the single-center cohort (15 male, 7 female) and categorized as non-invasive (Figure 4a).

In the single-center study cohort, a significant difference in the patient sex distribution dependent on the *emm* type could be identified (Figure 4b). Invasive SDSE cases associated with the *emm* type *stG62647* more frequently affected females (57.9%) than males (42.1%), whereas infections with other *emm* types showed the reverse trend, with 31.8% female and 68.2% male patients.

The remaining 15 *emm* types were not uniformly distributed between male and female patients (Figure 4c). Whereas males were affected by all 15 other *emm* types, invasive SDSE infections in females were associated with only seven, namely *stC74a* (*n* = 5), *stG6* (*n* = 4), and *stC839*, *stG480*, *stG485*, *stG2078*, and *stG5420* (*n* = 1 each).

Considering patient age, it was obvious that females affected by *stG62647* SDSE isolates were significantly older (median 70.5 years) than females associated with other *emm* types (median 47.0 years), whereas no substantial differences could be observed in the invasive SDSE infections of male patients (Figure 4d). Interestingly, excluding the *emm* type assignment, male (median 66.5 years) and female (median 66.0 years) cases showed no differences in age distribution.

Taking the type of isolation material into account, invasive SDSE isolates were more frequently identified in the blood of male patients (*stG62647*/other *emm* 31.25%/40.00%) than in females (*stG62647*/other *emm* 18.18%/21.43%) (Figure 4e). However, no statistically significant correlation could be identified.

**Figure 5:**
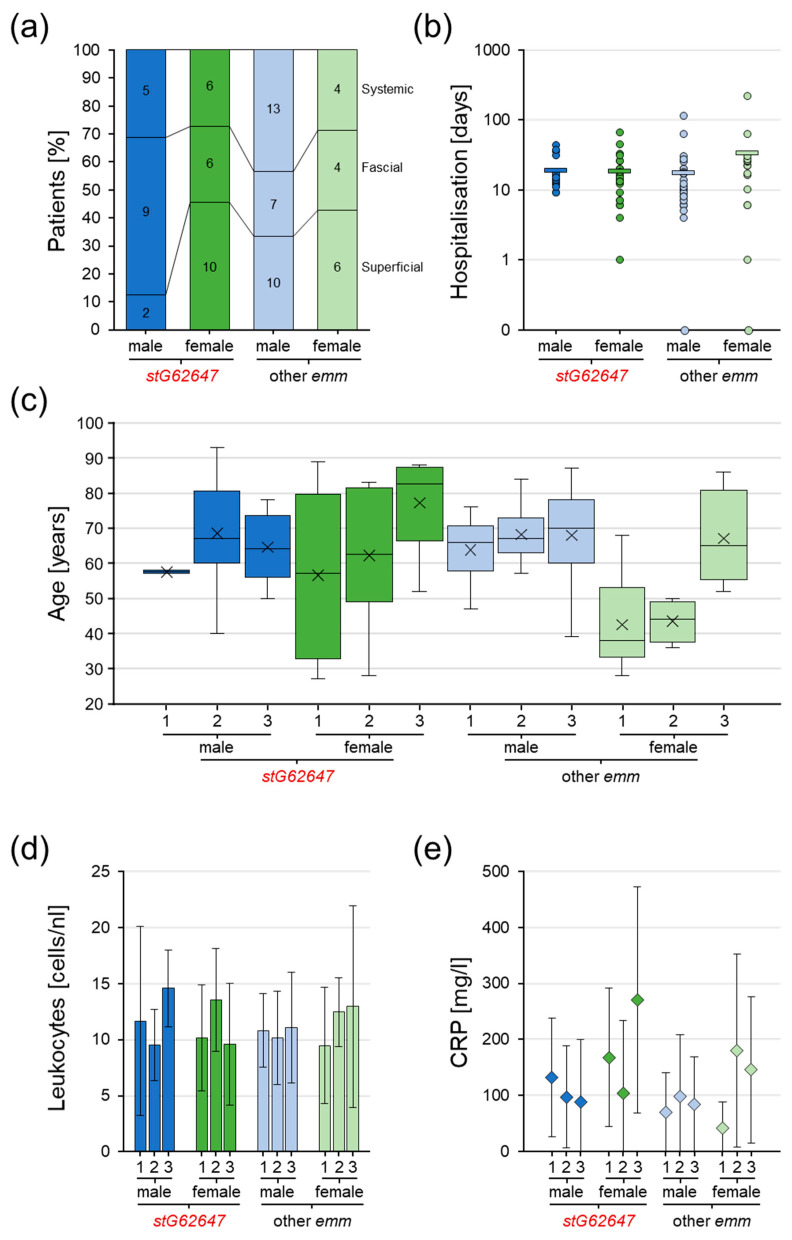
Assessment of Ingolstadt Hospital invasive SDSE case infection severity classification grouped into superficial (1), fascial (2), and systemic (3), separated into *stG62647* cases (dark colored) and cases associated with other *emm* types (bright colored), divided by male (blue) and female patients (green). (**a**) Distribution of infection severity separated by patient sex and *emm* type with total numbers (numbers inside columns). (**b**) Distribution of hospital stay duration separated by patient sex and *emm* type with single values (circles) and arithmetic mean (bar). (**c**) Distribution of patient age separated by infection severity, patient sex, and *emm* type with indicated median (line) and arithmetic mean (cross). (**d**) Distribution of leukocyte count in blood separated by infection severity, patient sex, and *emm* type indicated by arithmetic mean (columns) and standard deviation (whiskers). (**e**) Distribution of CRP concentration in blood separated by infection severity, patient sex, and *emm* type indicated by arithmetic mean (diamonds) and standard deviation (whiskers).

Further analyses were applied by categorizing the 82 invasive SDSE patients of the Ingolstadt Hospital single-center cohort into superficial, fascial, and systemic infections to assess the potential impact of *emm* type and patient sex. Male patients affected by SDSE *stG62647* more frequently developed fascial infections (*stG62647*/other *emm* 56.25/23.33%), but fewer superficial (12.50/33.33%) and systemic (31.25/43.33%) characteristics than males associated with other *emm* types, whereas female cases showed no obvious differences in the distribution of the infection severity classification (Figure 5a). Although it was obvious that infections in males were more severe compared to female patients, the difference did not reach statistical significance.

The duration of hospitalization showed no significant differences in the average hospital stay (Figure 5b). However, female patients affected by *emm* types other than *stG62647* stayed on average almost twice as long in the hospital (33 days) as females affected by *stG62647* (19 days) and males (other *emm*, 18 days; *stG62647*, 19 days). The variance for male patients affected by SDSE *stG652647* was slightly lower (6–43 days), compared to males infected by other *emm* types (0–129 days) as well as females (*stG62647*, 1–66 days; other *emm*, 0–217 days).

Combining the infection severity classification with patient sex and considering the patient age distribution, it was obvious that females suffering from superficial and fascial infections of *emm* types other than *stG62647* were distinctly younger (superficial median, 38 years; fascial median, 44 years) than female patients infected by *stG62647* (superficial median, 57 years; fascial median, 63 years) as well as males in general (Figure 5c). Male patients showed no significant differences in age distribution dependent on the infection severity. However, male patients suffering from fascial infections by SDSE *stG62647* were marginally older (median 67 years) than males systemically infected by this *emm* type (median 64 years), which is in contrast to males affected by other *emm* types and female patients. Besides these trends, no statistical significance could be obtained.

Considering general infection markers such as leukocyte cell count and C-reactive protein (CRP) concentration as indicators of infection severity to identify potential differences between *stG62647* and other *emm* types dependent on patient sex, it was obvious that the average values of both clinical markers did not consistently reflect the applied infection severity classification nor the observed patient age distribution (Figure 5d,e). The huge inter-patient variance within the different groups allowed no statistically significant conclusions.

### 3.6. S. dysgalactiae Infections Resembling S. pyogenes and S. agalactiae

**Figure 6:**
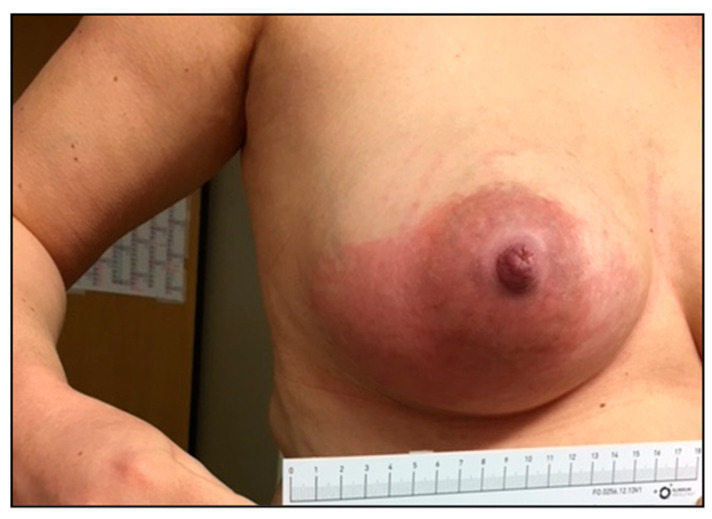
Photographic documentation of an SDSE *stG62647.0-*induced mastitis puerperalis in a 33-year-old mother (patient 2; Table S1) after giving her first birth.

Many infections caused by SDSE have shown disease patterns and courses resembling those of *S. pyogenes*. Among them are erysipelas, wound infections at various body sites, and implant-associated infections, such as endo- and periprosthetic infections, as well as nosocomial infections. Interestingly, within the observation period, four women who had recently given birth suffered from SDSE-induced mastitis puerperalis (patients 2, 3, and 4: *stG62647.0*; patient 51: *stG485.0*. See Table S1), whereas no cases of *S. pyogenes* mastitis occurred in Ingolstadt Hospital (Figure 6).

Apart from mimicking infections of *S. pyogenes*, SDSE also caused infections usually induced by *S. agalactiae*. An example was the infection of a newborn girl who developed a newborn infection but rapidly recovered after ampicillin and gentamicin application (case not included in study cohort). Interestingly, three years before, her mother had suffered from a postpartum infection caused by β-hemolytic group B streptococci after giving birth to her older daughter. This case suggests that, besides mediating infections typically caused by *S. pyogenes*, SDSE might also lead to infections usually associated with *S. agalactiae*, and even may substitute this bacterial species in predisposed patients.

## 4. Discussion

The incidence of invasive *Streptococcus dysgalactiae* ssp. *equisimilis* infections is rising worldwide [16,17,18,19,20,21,22,23], with one *emm* type, denoted *stG62647*, constantly increasing [46,47,48,49,50,51,52,53]. Even though a comparative genome analysis approach revealed the potential involvement of a mutated virulence regulator [50], a comprehensive explanation for the evolutionary and pathogenic success of this particular *emm* type is still lacking.

The presented work combines national surveillance data with a single-center clinical study, on one hand, to provide an epidemiologic overview of invasive SDSE in Germany, and, on the other hand, to highlight recent developments in the *emm* type distribution and related clinical aspects with a special focus on the very successful type *stG62647*. While the national study cohort gives insight into changes in the *emm* type ratio in the study years 2010 to 2022, with additional attention to microbiological, geographical, and sample characteristics, the single-center cohort intended to obtain a representative cross-section of the total SDSE burden at a single hospital site in Germany concentrating on patient characteristics, classified disease severity, and clinical infection markers.

The results of this combinatory approach suggest an increasing infection burden for the German population, indirectly indicated by the continuously increasing number of invasive SDSE isolates reported to the GNRCS from 31 in 2010 to 239 in 2022 (Figure 1c). However, it is not clear if this increase is indeed the result of extended bacterial spread, rising invasiveness, or just an increased awareness of SDSE infections within the observation period. Since epidemiological data on SDSE colonization are not available for Germany, the reason for the increased number of invasive infections remains unclear. Furthermore, epidemiologic data from the SARS-CoV-2 pandemic with the institution of non-pharmaceutical interventions, such as face masks and social distancing, suggest that carriage, transmission as well as infection initiation of SDSE are not well understood and might differ from what is known for its close relative *S. pyogenes* [47].

At Ingolstadt Hospital, the overall number of SDSE cases between 2010 and 2022 increased only to a very minor extent, although with great variation in the number of isolates per year, ranging from 56 cases in 2010 and 94 cases in 2012 (Figure S1). Therefore, enhanced virulence or improved attention to SDSE infections provide probable explanations for the rising numbers of invasive SDSE reported to the GNRCS. The potential change in awareness of this particular bacterial species might have been facilitated by technical innovations in microbiological routine diagnostics. Within the study period, many diagnostic laboratories in Germany shifted the streptococcal identification routine away from serotyping, only enabling the classification of isolates by Lancefield antigen groups, to species identification by MALDI-TOF MS [58]. At Ingolstadt Hospital the technique was introduced in January 2015 [59]. As the MALDI-TOF system in most cases provides the taxonomic notation of a bacterial isolate, it can be assumed that the introduction of this technique contributed to increased submission rates of *S. dysgalactiae* ssp. *equisimilis*.

In Germany, a general trend of an aging population is observed [60], which leads to a shift in the incidence of many different diseases including bacterial infections [61]. Surveillance data show invasive SDSE infections and severity primarily increasing with age (Figure 5c), mainly affecting older people [23,47,52,62]. This shift in population composition might also contribute to the observed increase in invasive SDSE infections reported to the GNRCS, potentially overlaid by a severity bias leading to increased submission rates of conspicuous cases.

The rising number of invasive SDSE infections in the national study cohort between 2010 and 2022 might be the result of multifactorial changes in bacterial virulence, clinical awareness, and population structure, potentially not reflecting a general trend. However, the change in *emm* type distribution toward one very successful type, *stG62647*, cannot be neglected (Figure 1c). The performed linear regression, even though not the ideal model to describe the observed trend in *emm* type distribution, clearly showed a significant increase in *stG62647* in the national study cohort (Figure 1d). None of the other *emm* types is characterized by a comparable slope above a value of 0.3, while *stG62647* on average increased by 1.8% per year. However, the corresponding coefficient of determination, although depicting the highest reliability within all *emm* types in the study cohort, shows a comparably low value of 0.67, clearly indicating a deviation from an ideal linear trend.

The main reason for this impaired regression fitting, besides the drop in 2022, seems to be the sudden increase in *stG62647* isolates in 2013 (Figure 1c), which was initially observed in Norway by Oppegaard et al., in 2017 [50], and could now be confirmed by national surveillance data from Germany. Whether the observed rise in *stG62647* in 2013 was initiated by the described mutation event in the virulence regulator needs to be clarified by a comparative genome approach including *stG62647* strains isolated before 2013. However, this sudden increase within one year strengthens the hypothesis of a virulence-driven outbreak, representing a potential origin of the observed dominance of this particular *emm* type.

One possible reason, besides increased virulence, might be the stG62647 M protein itself. Even though not much is known about the functional repeat structure of this M type, the highly variable N-terminal region seems to be a very successful antigenic variant. Assessing the subtype distribution of all *emm* types in the national study cohort, it becomes obvious that *stG62647* produced a comparatively low number of *emm* subtypes in relation to the corresponding number of reported infection events. Furthermore, the subtype distribution within this particular *emm* type is extremely shifted toward *stG62647.0*, which is clearly indicated by very low diversity indices (Figure 2c). With a Shannon index of 0.14 and an evenness of 0.06, *stG62647* has the lowest intra-*emm*-type diversity of all *emm* types in the study cohort. However, whether this implies a superior antigenic structure needs to be addressed by additional immunological studies. These observations suggest a global outbreak of an *stG62647.0* clone, spontaneously mutated in or before 2013, that turned out to be very successful. Interestingly, *stG62647.0* occurs in two different variants, expressing mainly Lancefield antigen C, but also Lancefield antigen G (Figure 2a). Oppegaard et al. reported solely group C *stG62647* isolates [50], which reflects the situation in the Ingolstadt Hospital cohort (Table S1), possibly a result of low case numbers in these single-center studies. However, a first step to address the hypothesis of a clonal origin of the observed *stG62647* rise could be the tracking of the virulence-regulator mutation in the *sliB*-gene in *stG62647* isolates expressing the Lancefield antigen G.

The question of the chronological and geographical origin of this potential clonal outbreak will most probably remain unanswered. The assessment of patient residence data of the national study cohort revealed no geographical accumulation of invasive SDSE *stG62647* cases in German federal states (Figure 3a).

The analysis of additional patient data available in the national study cohort showed that around 90% of *stG62647* and non-*stG62647* isolates had been identified in blood cultures, indicating that the majority of reported SDSE isolates had indeed caused not only invasive but also systemic infections, which argues against the hypothesis that non-*stG62647 emm* types per se exhibit lower pathogenicity (Figure 3b).

The majority of invasive SDSE was isolated from male patients, independently of the *emm* type. However, the percentage of affected males was slightly higher when infected by non-*stG62647* strains (Figure 3c). A comparable trend was observed in the single-center cohort of Ingolstadt Hospital, where the proportion of male patients affected by *stG62647* was lower than the percentage of males affected by other *emm* types (Figure 4b). However, in contrast to the national cohort, in Ingolstadt Hospital *stG62647* was isolated more frequently from female patients, whereas the resulting low numbers of female non-*stG62647* cases may explain the observed differences in *emm* type diversity (Figure 4c). Nevertheless, the reason for this discrepancy between the two study cohorts is not clear. In Ingolstadt Hospital, the ratio of male to female patients suffering from *stG62647* bloodstream infections was almost similar to that of non-*stG62647* affected patients (Figure 4e). Taking the patient sex into account, invasive SDSE were more frequently isolated in the blood of male patients, than of females, but only males affected by non-*stG62647 emm* types showed a higher number of systemic infections, whereas the *stG64647* cases were more frequently associated with fascial infections (Figure 5a) accompanied by a slightly higher age (Figure 5c). Unfortunately, the only other single-center study from Germany assessing the *emm* type distribution of SDSE infections did not describe the ratio of affected male and female patients nor did it address infection severity [46]. Since skin and mucous membranes of males and females exhibit significant differences in their composition [63,64,65], the molecular interactions of SDSE virulence factors, such as M protein, might be affected, which makes a sexual preference of particular *emm* types possible. Therefore, further studies are needed to examine whether particular *emm* types prefer colonization of male or female humans.

In the single-center cohort from Ingolstadt Hospital, female patients affected by invasive SDSE showed significant *emm*-type-dependent age differences, with females affected by non-*stG62647* isolates being about 20 years younger than females suffering from *stG62647* infections, and males in general (Figure 4d). This effect could not be explained by differences in isolation material (Figure 4e) nor infection severity (Figure 5a) and had no obvious influence on the duration of hospitalization (Figure 5b). However, the combined analysis of *emm* type, patient sex, and infection severity clearly showed that this age effect was mainly based on non-*stG62647* superficial and fascial infections affecting younger women (Figure 5c). In addition to that, a general trend of increasing infection severity with patient age could be observed. Unfortunately, the *emm*-type-dependent age effect in females as well as the general trend of increasing infection severity with growing age was not reflected by infection markers such as leukocyte count (Figure 5d) or CRP concentration (Figure 5e), probably because of immense variation between patients, induced by low case numbers in the subgroups of the single-center cohort. Additionally, it needs to be argued that the applied infection severity classification, even though based on diagnostic data, isolation material information, and clinical infection values, represents a subjective categorization, prone to bias. However, the observation that females are less affected by non-*stG62647* SDSE but at a younger age is of high importance and needs to be further elucidated by other single-center studies.

The presented data clearly show a dominance of *stG62647* in invasive SDSE infections in Germany. However, a sudden drop in 2022 might indicate a reverse trend in the coming years (Figure 1c). The assessment of antimicrobial resistance data shows slightly lower non-susceptibility to erythromycin, clindamycin, and tetracycline in *stG62647* isolates, compared to other *emm* types. Before 2013, *stG62647* was only rarely identified in invasive SDSE infections. The first time isolates of this *emm* type were received at the GNRCS was 2005 (*n* = 2) and in the literature, it was first mentioned in 2016 [48]. Therefore, *stG62647* was most likely not frequently confronted with antibiotics, a potential explanation for the higher susceptibility observed for erythromycin, clindamycin, and tetracycline. However, whether this leads to a substantial advantage in the treatment of invasive SDSE infections remains to be shown.

A much more concerning observation is the potential replacement of infections, usually associated with β-hemolytic pathogens such as *S. pyogenes* and *S. agalactiae* by *S. dysgalactiae* ssp. *equisimilis*, especially considering the observed increase in case numbers (Figure 1c). It is well known that SDSE shows a similar pathogenic potential as *S. pyogenes*, inducing severe tissue infections, sepsis, and toxic shock [9,10,11,12,13,14]. However, the isolation of invasive SDSE strains from diseases commonly caused by *S. agalactiae* such as mastitis or infections in newborns [66,67], as described here (Section 3.5) and also reported to the GNRCS, gives rise to serious concern. The impact of this potential pathogenicity shift on public health is currently not assessable but needs close monitoring.

## 5. Conclusions

In recent years, the number of invasive *Streptococcus dysgalactiae* subspecies *equisimilis* isolates reported to the GNRCS has continuously increased. However, it is not clear whether this increase is based on a biological phenomenon, a change in clinical awareness, or population structure. Within the observation period, the proportion of *emm* type *stG62647* increased from 23% to 46% with a drop to 37% in 2022, and this type seems to exhibit increased virulence. In older patients, infections were more severe, and men are more prone to invasive SDSE infections than women, although in the single-center cohort, this trend was reversed in patients with *stG62647*, who mainly developed fascial infections. Women suffering from superficial and fascial infections by non-*stG62647* SDSE were significantly younger than other patients. The observed sex-associated differences need further studies to elucidate the epidemiology and underlying mechanisms of the suggested clonal outbreak scenario.

## Figures and Tables

**Table 1:** Invasive SDSE infections at Ingolstadt Hospital from December 2016 to November 2022.

Sex	Male 46 (56.1%)	Female 36 (43.9%)		
**Age** [years]	Median 65	Range 27–93		
**Material** [sampling]	Blood 24 (29.3%)	Swab 21 (25.6%)	Sore smear 19 (23.2%)	Other 18 (21.9%)
**Infection** [classification]	Superficial 28 (34.1%)	Fascial 26 (31.7%)	Systemic 28 (34.1%)	
**Sampling** [days]	Average 3.7	Range −5–175		
**Hospital stay** [days]	Average 20.8	Range 0–217		
**Lancefield** [antigen]	C 40 (48.8%)	G 42 (51.2%)		
**Genotype** [*emm* type]	*stG62647* 38 (46.3%)	Other 44 (53.7%)		

Material: sampling material; infection: classification of infection severity; sampling: number of days from patient admission to sampling; hospital stay: number of days from patient admission to discharge from hospital.

## Data Availability

All data are included within the article and the Supplementary Materials.

## Supplementary Materials

The following supporting information can be downloaded at: https://www.mdpi.com/article/10.3390/pathogens12040589/s1, Figure S1. Numbers of total SDSE infection cases treated at Ingolstadt Hospital between 2010 and 2022. Table S1. Patients colonized and/or infected with SDSE at Ingolstadt Hospital admitted between December 2016 and November 2022. Table S2. Invasive SDSE infections in Germany from 2010 to 2022 reported to the GNRCS.
