# Supplementary material for: Epidemiological and Clinical Features of Streptococcus dysgalactiae ssp. equisimilis stG62647 and Other emm Types in Germany
Source: Pathogens. 2023 Apr 13;12(4):589. doi: 10.3390/pathogens12040589 (PMC10143538; doi:10.3390/pathogens12040589)
Supplement: Supplementary file 1 [file pathogens-12-00589-s001.zip › pathogens-2283318-supplementary.pdf]

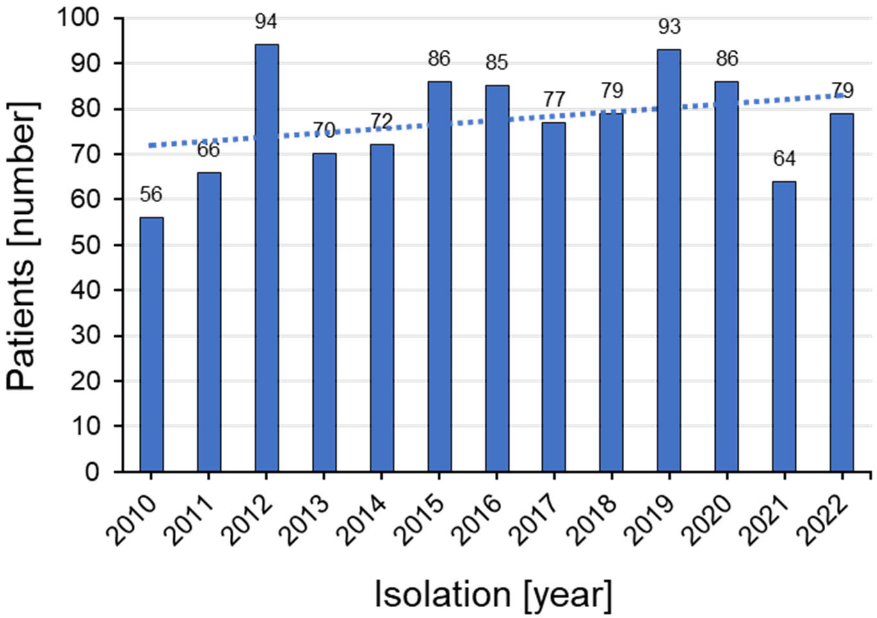

Figure S1. Numbers of total SDSE infection cases treated at Ingolstadt hospital between 2010 and 2022 (blue bars) with linear regression (dotted line).

Table S1. Patients colonized and/or infected with SDSE at Ingolstadt hospital admitted between December 2016 and November 2022.

**Number**	**Sex**	**Age** [years]	**Diagnosis**	**Material**	**Infection** [classification]	**Invasivity** [classification]	**EMM** [type]	**Lancefield** [antigen]	**Sampling** [days after admission]	**Hospital stay** [days]	**Leucocyte** [cells/nl]	**CRP** [mg/l]	**LDH** [U/l]	**CPK** [U/l]	**GOT** [U/l]
1	male	57	Erysipelas lower leg, Malum perforans infection toe	Biopsy	Superficial	invasive	*stG62647.0*	G	3	35	5.7	207.4		42	53
2	female	33	Mastitis puerperalis	Secretion breast	Superficial	invasive	*stG62647.0*	G	1	6	7.2	199.8			10
3	female	32	Mastitis puerperalis	Sore smear	Superficial	invasive	*stG62647.0*	G	0	14	7.8	152.8			16
4	female	33	Mastitis puerperalis	Sore smear	Superficial	invasive	*stG62647.0*	C	0	1					
5	male	58	Abscess forearm, Soft tissue tumor	Sore smear	Superficial	invasive	*stG62647.0*	G	0	9	17.6	56.8			
6	female	27	Endometritis	Swab vagina	Superficial	invasive	*stG62647.0*	G	0	4	14	222.9			10
7	female	44	Abscess, Varicose veins, Ankle joint fusion, Obesity, Smoker	Swab wound	Superficial	invasive	*stG62647.0*	G	3	16	6	4.1			
8	female	70	Wound infection, Dilated cardiomyopathy, Diabetes mellitus, Obesity	Swab wound	Superficial	invasive	*stG62647.0*	G	8	26	6.7	6.1	171		10

9	female	79	Ulcerations lower leg, Sepsis	Swab wound	Superficial	invasive	*stG62647.0*	G	0	15	13.8	278.9	129		10
10	female	77	Wound debridement lower leg, Ischemia, Peripheral artery disease	Swab wound	Superficial	invasive	*stG62647.0*	G	0	12	19.7	231.8		168	
11	female	82	Urinary tract infection, Sepsis, Lumbar spine fracture	Urine	Superficial	invasive	*stG62647.0*	G	4	13	6.3	55.6	201		17
12	female	89	Urinary tract infection, Erysipelas lower leg	Urine	Superficial	invasive	*stG62647.0*	G	0	13	9.9	356.6	184		26
13	male	63	Periprosthetic infection upper leg, Sepsis	Aspirated fragment	Fascial	invasive	*stG62647.0*	C	0	12	12.3	281			
14	female	81	Empyema of left hip	Biopsy	Fascial	invasive	*stG62647.0*	C	0	33	14.8	172.3		20	14
15	female	56	Mummified toes, Necrosis	Biopsy	Fascial	invasive	*stG62647.0*	G	12	26	18.7	48.2	270		43
16	female	66	Osteoarthritis knee	Biopsy	Fascial	invasive	*stG62647.0*	C	0	20	9.3	338.9			
17	male	40	Empyema knee joint, Drug abuse	Puncture knee	Fascial	invasive	*stG62647.0*	C	1	15	8.4	171.3	1020		

18	male	69	Ulcus ball of foot, Sepsis	Sore smear	Fascial	invasive	*stG62647.0*	G	0	16	7.5	61.5		
19	male	80	Necrosis Achilles tendon	Sore smear	Fascial	invasive	*stG62647.0*	C	2	31	9.3	17.5	124	
20	female	83	Pressure ulcer buttocks, Parkinson's disease	Sore smear	Fascial	invasive	*stG62647.0*	G	0	31	7.9	35.9		
21	male	67	Wound infection, Amputation of big toe, Korsakoff syndrome	Sore smear	Fascial	invasive	*stG62647.0*	G	1	12	13.1	143.2		13
22	male	66	Skin necrosis foot, Stasis dermatitis	Sore smear	Fascial	invasive	*stG62647.0*	C	0	13	6.6	31.7	252	155
23	female	28	Preterm uterine contractions, Caesarean section	Swab uterine cavity	Fascial	invasive	*stG62647.0*	G	0	6	18.4	4.1		29
24	male	81	Osteomyelitis index finger, Hereditary spastic paraplegia	Swab wound	Fascial	invasive	*stG62647.0*	C	2	9	8.5	127.8		10
25	female	59	Abcess gamma nail left hip, Sepsis, Diabetic gangrene	Swab wound	Fascial	invasive	*stG62647.0*	C	2	9	12.1	21.6		
26	male	93	Erysipelas lower leg, Toe amputation	Swab wound	Fascial	invasive	*stG62647.0*	C	0	17	5.2	27.1		

27	male	57	Ulcerations feet, Arteriovenous occlusive disease, Diabetes mellitus, Hypertonia	Swab wound	Fascial	invasive	*stG62647.0*	G	0	14	14.7	12.5			
28	female	71	Infection of total knee endoprosthesis	Aspirated fragment	Systemic	invasive	*stG62647.0*	C	0	45	5.8	424.7	230	92	26
29	female	80	Bruise on knee, Breast cancer, Brain metastasis	Blood culture	Systemic	invasive	*stG62647.0*	C	1	7	3.5	493.8			
30	male	64	Erysipelas lower leg	Blood culture	Systemic	invasive	*stG62647.0*	C	1	15	14.9	60.4			114
31	male	78	Erysipelas of lower leg, Sepsis, Pneumonia, Diabetes mellitus, Spastic hemiplegia	Blood culture	Systemic	invasive	*stG62647.0*	C	0	43	18.5	96	161		14
32	male	50	Pflegmon lower leg, Spesis	Blood culture	Systemic	invasive	*stG62647.0*	G	0	11	11.8	6.1		167	63
33	female	85	Periocular erysipelas, Anaphylaxis	Blood culture	Systemic	invasive	*stG62647.0*	G	0	20	12.4	88.1			20
34	female	88	Erysipelals lower leg, Spesis, Chronic obstructive pulmonary disease	Blood culture	Systemic	invasive	*stG62647.0*	C	0	7	16.1	18.2	201	76	15
35	male	69	Diabetic foot syndrome, Sepsis	Blood culture	Systemic	invasive	*stG62647.0*	C	0	15	10.5	7.8	224		252

36	female	52	Soft tissue infection, Squamous-cell carcinoma	Blood culture	Systemic	invasive	*stG62647.0*	G	4	18	5.1	175.5	186		61
37	male	62	Erysipelas, Diabetes mellitus, Renal failure, Heart failure	Blood culture	Systemic	invasive	*stG62647.0*	C	1	37	17.1	274.3	428	2476	127
38	female	87	Abcess gamma nail left hip, Sepsis	Implant	Systemic	invasive	*stG62647.0*	G	1	66	14.6	426			8
39	male	41	Pneumonia, Sepsis	Secretion tracheal	Superficial	non invasive	*stG62647.0*	G	0	6	5.4	76	119		29
40	female	0	Newborn infection, Preterm birth	Swab throat	Superficial	non invasive	*stG62647.0*	C	1	6	15.8	6.7			
41	female	31	Appendicitis	Swab vagina	Superficial	non invasive	*stG62647.0*	G	5	10	9.3	129.9			21
42	male	69	Prostate carcinoma, Hypertension, Hypercholesterolemia	Urine	Superficial	non invasive	*stG62647.0*	G	0	13	6	1.8			
43	male	80	Urinary tract infection, Sepsis, Pneumonia	Urine	Superficial	non invasive	*stG62647.0*	G	0	12	14	14.2	147		12
44	male	65	Femur fraction	Urine	Superficial	non invasive	*stG62647.0*	G	0	39	7.3	97.4	247		

45	male	72	Renal failure, Viral pneumonia	Urine	Superficial	non invasive	*stG62647.0*	C	1	17	6.8	85	379		30
46	female	57	Sepsis, Cerebral infarction	Urine	Superficial	non invasive	*stG62647.0*	G	4	50	8	57.3		47	39
47	male	77	Chronic skin inflammation penis, Benign prostatic hyperplasia	Urine	Systemic	non invasive	*stG62647.0*	C	0	11	11.9	15.4	2033	2985	748
48	female	40	Ascites, Hemihepatectomy, Cholangiocarcinoma	Aspirated fragment	Superficial	invasive	*stC74a.0*	C	1	16	17.1	108.8			22
49	male	48	Soft tissue infection, Compartment syndrom lower leg	Biopsy	Superficial	invasive	*stC839.2*	G	5	10	5.3	2		133	
50	male	65	Gangrene, Osteolysis metatarsus	Sore smear	Superficial	invasive	*stG485.0*	C	0	20	15.6	45.3	181		27
51	female	35	Mastitis puerperalis	Sore smear	Superficial	invasive	*stG485.0*	G	0	22					
52	male	76	Ulcerations feet, Arteriovenous occlusive disease	Sore smear	Superficial	invasive	*stG643.0*	G	0	9	8.4	14.1			
53	male	67	Running sore, Stasis dermatitis, Diabetes mellitus, Hypertension, Alcohol dependence	Swab lower leg	Superficial	invasive	*stG485.0*	C	42	114	9.8	11.3		107	19

54	male	47	Phlegmone penis	Swab urether	Superficial	invasive	*stG5420.0*	G	0	6	12.2	145.7			
55	male	62	Scrotal abscess	Swab urether	Superficial	invasive	*stG480.0*	C	0	11	12.7	112.2			
56	male	70	Wound infection, Traumatic brain injury	Swab wound	Superficial	invasive	*stC74a.0*	C	1	20	15.6	45.3			22
57	female	28	Chest inflammation	Swab wound	Superficial	invasive	*stC74a.0*	G	0	0					
58	female	48	Wound infection, Atopic dermatitis, Cancer, Psychosis	Swab wound	Superficial	invasive	*stC74a.12*	G	175	217	6.7	16.2			
59	male	73	Ulcerations, Gout	Swab wound	Superficial	invasive	*stG10.0*	G	1	9	10	88.8			
60	male	61	Ulcerations feet, Arteriovenous occlusive disease	Swab wound	Superficial	invasive	*stG480.0*	C	-5	4	8.3	13.6			
61	male	69	Erysipelas lower leg, Peripheral artery disease	Swab wound	Superficial	invasive	*stG480.0*	C	0	6	10.1	217.8			
62	female	68	Ulcerations, Arteriovenous occlusive disease	Swab wound	Superficial	invasive	*stG6.1*	C	0	25	8.4	37.5		33	510

63	female	36	Bladder rupture, Sepsis, Pressure ulcer, Persistent vegetative state	Urine	Superficial	invasive	*stG6.1*	G	0	6	5.7	5.8	145	116
64	male	57	Chronic osteomyelitis, Amputation of second toe	Biopsy	Fascial	invasive	*stG245.0*	G	0	6	10.1	37.5		16
65	male	73	Prepatellar bursitis, Hypertension	Biopsy	Fascial	invasive	*stG480.0*	C	2	10	15	138.9		
66	female	46	Osteitis, Sepsis	Implant	Fascial	invasive	*stG5420.0*	G	0	26	16.1	279.3		
67	male	69	Dermal necrosis, Arteriovenous occlusive disease	Sore smear	Fascial	invasive	*stC74a.0*	C	9	18	5	4		
68	male	67	Erysipelas, Deep vein thrombosis	Sore smear	Fascial	invasive	*stC74a.0*	G	10	30	9.9	301.7	222	24
69	male	63	Empyema upper ankle joint, Sepsis	Sore smear	Fascial	invasive	*stC839.8*	C	0	14	8.3	28.6		
70	male	84	Abscess of forefoot, Gout, Hypercholesterolemia	Sore smear	Fascial	invasive	*stG11.0*	C	0	9	16.2	13.4		
71	female	50	Phlegmone lower leg after surgery, Sepsis	Sore smear	Fascial	invasive	*stG480.0*	C	4	17	11.5	371.2	181	14

72	female	42	Pyoderma gangrenosum lower legs, Soft tissue damage, Hepatitis B/C, Drug abuse	Sore smear	Fascial	invasive	*stG6.1*	C	0	10	8.8	48.1			6
73	male	64	Ulcerations feet, Sepsis, Seizures, Middle cerebral artery infarction	Sore smear	Fascial	invasive	*stG643.1*	G	0	62	6.6	167			
74	female	36	Necrotic soft tissue infection lower leg	Swab wound	Fascial	invasive	*stC74a.12*	C	9	27	13.4	20.6			
75	male	78	Periprosthetic infection knee, Erysipelas	Aspirated fragment	Systemic	invasive	*stG245.0*	G	1	25	4.4	195.8			11
76	male	67	Erysipelas of lower leg	Blood culture	Systemic	invasive	*stC46.0*	C	0	6	18.2	25.1		236	53
77	male	71	Infection exacerbation, Chronic obstructive lung disease, Coronary artery disease	Blood culture	Systemic	invasive	*stC5345.0*	C	3	11	11.6	3.6	190		20
78	female	52	Port infection, Spesis, Neutropenia, Breast cancer	Blood culture	Systemic	invasive	*stC74a.0*	C	0	30	0.2	242.5		521	123
79	male	39	Injection abcess, Thrombose internal iliac vein	Blood culture	Systemic	invasive	*stC74a.12*	C	0	17	12.9	189	358	66	31
80	female	86	Erysipelas lower leg, Sepsis, Kardial decompensation	Blood culture	Systemic	invasive	*stC839.2*	C	0	62	19.7	50.7	327		72

81	male	58	Osteomyelitis metatarsus	Blood culture	Systemic	invasive	*stG10.0*	G	0	8	14.1	58.7			71
82	male	86	Infection of unknown origin, Diabetes mellitus, Obesity	Blood culture	Systemic	invasive	*stG166b.0*	C	0	7	15.4	47.3	139		9
83	male	74	Erysipelas, Bladder cancer, Prostate cancer	Blood culture	Systemic	invasive	*stG2078.0*	G	0	7	17.1	15.2	197	145	19
84	male	63	Sepsis, Prostate carcinoma	Blood culture	Systemic	invasive	*stG2078.0*	C	0	5	11.3	15.3	273		
85	male	50	Pneumonia, Sepsis, Hepatitis C, Drug abuse, Smoker	Blood culture	Systemic	invasive	*stG2574.3*	C	0	12	7.7	260	240	1152	51
86	male	78	Erysipelas lower leg, Diabetes mellitus	Blood culture	Systemic	invasive	*stG485.0*	C	0	17	10.6	115.5			33
87	male	87	Infection of unknown origin, Chronic obstructive lung diesease, Kidney failure	Blood culture	Systemic	invasive	*stG485.0*	C	0	8	1.7	120.2	207	1960	86
88	male	70	Erysipelas of lower leg	Blood culture	Systemic	invasive	*stG6.0*	G	0	17	12.7	5.7			19
89	male	62	Diatbetic foot gangrene, Sepsis	Blood culture	Systemic	invasive	*stG6.0*	G	0	27	5.9	45.7	209	309	14

90	female	65	Erysipelas lower leg, Leg vein thrombosis	Blood culture	Systemic	invasive	*stG6.1*	G	0	6	18.9	16.2			79
91	female	65	Several wounds on limbs, Sepsis	Sore smear	Systemic	invasive	*stG2078.0*	G	1	1	13	273	210		10
92	male	74	Metastatic cholangiocellular carcinoma	Secretion bronchial	Superficial	non invasive	*stC36.7*	C	26	49	4.4	17.2			14
93	male	64	Pneumoniae, Urinary tract infection, Sepsis, Traumatic brain injury, Hydrocephalus	Secretion bronchial	Superficial	non invasive	*stG485.0*	G	1	7	11.3	134.5	279		12
94	female	63	Lung metastases, Breast cancer	Secretion tracheal	Superficial	non invasive	*stC839.2*	G	6	14	9.9	14	176		27
95	male	55	Prostate cancer, Spesis	Swab rectum	Superficial	non invasive	*stG652.2*	G	0	0	6.6	1.8			
96	male	34	Mixed intoxication, Hepatitis B/C	Swab urether	Colonization	non invasive	*stC74a.0*	G	52	129	9.6	1			
97	male	74	Kidney insufficiency, Synkope with fall, Diabetes mellitus, Obesity	Urine	Colonization	non invasive	*stC36.7*	G	6	10	8.1	2.7		236	17
98	female	71	Urinary tract infection with E. coli, Sepsis, Kidney insufficiency, Wound infection lower [illegible]	Urine	Superficial	non invasive	*stC74a.0*	C	2	10	7.6	142.8	194		14

99	male	39	Scrotal cancer	Urine	Superficial	non invasive	*stG166b.0*	G	0	8	3	25.3	224		6
100	male	85	Prostate cancer, Transitional cell carcinoma	Urine	Superficial	non invasive	*stG485.0*	G	0	0	7.4	1.1			
101	female	75	Thalamus infarction, Coronary artery disease, Syncope, Diabetes mellitus	Urine	Superficial	non invasive	*stG643.0*	C	5	11	6.9	97		183	
102	female	31	Endomyometritis	Swab vagina	Fascial	non invasive	*stC839.2*	G	-2	1	14.2	24.4			30
103	male	60	Kidney failure, Transuretral resection, Prostatic hyperplasia	Urine	Fascial	non invasive	*stC74a.0*	G	0	1	12.4	10.1			
104	male	54	Prostate cancer, Sepsis	Urine	Systemic	non invasive	*stG643.0*	C	1	5	19	226.8	171		

Number: Sequential patient number; Diagnosis: Primary diagnosis and und underlying diseases Material: Sampling Material for microbiological examination; Infection: Classification of infection severity; Invasivity: Classification of invasivity of SDSD infection; EMM: Allotted *emm*-type of the corresponding SDSE isolate; Lancefield: Lancefield-antigen of the corresponding SDSE isolate; Sampling: Number of days from patient admission to sampling; Hospital stay: Number of days from patient admission to discharge from hospital; Leukocyte: Leukocyte cell count in blood in cells/nl; CRP: C-reacive protein concentration in blood in mg/l; LDH: lactate dehydrogenase concentration in blood in units/l; CPK: creatine phosphokinase concentration in blood in units/l; AST: aspartate aminotransferase concentration in blood in units/l

Table S2. Invasive SDSE infections in Germany 2010 to 2022 reported to the GNRCS

Number	Sex	Age [years]	Federal state	Diagnosis	Material	Isolation [date]	Lancefield [antigen]	EMM [type]	Penicillin [µg/ml]	Amoxicillin [µg/ml]	Cefotaxime [µg/ml]	Vancomycin [µg/ml]	Erythromycin [µg/ml]	Clindamycin [µg/ml]	Chloramphenicol [µg/ml]	Tetracyclin [µg/ml]	Levofloxacin [µg/ml]
102708	M	69	NI	no data	Blood	19.12.2022	G	*stC74a.0*	0,015	0,03	0,03	0,5	4	0,12	4	0,5	1
102388	n/a	90	BW	Infekt unklarer Genese	Blood	25.12.2022	C	*stG62647.0*	0,015	0,015	0,015	0,5	0,12	0,12	2	0,25	1
102300	F	56	BY	no data	Blood	26.12.2022	C	*stG62647.0*	0,015	0,03	0,03	0,5	0,12	0,12	4	0,5	0,5
102293	M	76	NW	Sepsis	Blood	29.12.2022	C	*stG62647.0*	0,015	0,03	0,03	0,5	0,12	0,12	2	0,5	1
102194	F	81	NW	no data	Blood	22.12.2022	G	*stG10.0*	0,015	0,03	0,03	0,5	0,12	0,12	4	64	1
102193	F	0	NW	no data	Blood	21.12.2022	G	*stG10.0*	0,015	0,03	0,03	0,5	0,12	0,12	4	64	1
102064	F	70	NW	Sepsis	Blood	24.12.2022	G	*stG10.0*	0,015	0,03	0,03	0,5	0,12	0,12	4	64	1
101980	M	71	SH	Sepsis, Erysipel, sept. Arthritis, Phlegmone	Blood	19.12.2022	G	*stG2078.0*	0,015	0,03	0,03	0,5	0,12	0,12	4	0,5	1
101979	M	83	SH	Erysipel	Blood	18.12.2022	C	*stG62647.0*	0,015	0,03	0,03	0,5	0,12	0,12	4	0,5	1
101900	F	90	NW	Sepsis	Blood	20.12.2022	G	*stG643.0*	0,015	0,015	0,03	0,5	0,12	0,12	4	32	0,5
101692	M	89	NW	Sepsis, Wundinfektion	Blood	12.12.2022	C	*stG62647.0*	0,015	0,015	0,03	0,5	0,12	0,12	4	0,5	1
101625	F	89	NI	no data	Blood	06.12.2022	G	*stG485.0*	0,015	0,03	0,03	0,5	0,12	0,12	4	1	1
101550	F	87	NI	Erysipel	Blood	12.12.2022	C	*stG62647.0*	0,015	0,015	0,015	0,5	0,12	0,12	4	0,5	1
101539	M	66	SH	no data	Blood	05.12.2022	G	*stG643.0*	0,015	0,03	0,03	0,5	0,12	0,12	4	64	0,5
101487	M	72	SN	Sepsis, Erysipel	Blood	09.12.2022	G	*stG2078.0*	0,015	0,015	0,03	0,5	0,12	0,12	4	0,5	1
101476	M	65	SH	no data	Blood	06.12.2022	C	*stG62647.0*	0,015	0,03	0,03	0,5	0,12	0,12	4	0,5	1
101421	M	77	BY	Sepsis	Blood	03.12.2022	G	*stG643.0*	0,015	0,015	0,015	0,5	0,12	0,12	4	32	0,5
101379	M	81	HE	no data	Blood	06.12.2022	C	*stG62647.0*	0,015	0,03	0,03	0,5	0,12	0,12	4	0,5	1
101345	M	85	NW	no data	Blood	04.12.2022	C	*stG6.1*	0,015	0,03	0,03	0,5	0,12	0,12	4	0,5	1
101309	M	65	NW	Sepsis	Blood	01.12.2022	G	*stC5345.0*	0,015	0,03	0,03	0,5	0,12	0,12	4	0,5	1
101227	M	69	SH	Erysipel	Blood	23.11.2022	C	*stG62647.0*	0,015	0,015	0,03	0,5	0,12	0,12	2	0,25	1
101196	M	78	SN	Sepsis, Erysipel	Blood	27.11.2022	C	*stG62647.0*	0,015	0,03	0,03	0,5	0,12	0,12	4	0,5	0,5
101177	F	52	RP	no data	Blood	21.11.2022	G	*stC74a.0*	0,015	0,015	0,015	0,5	0,12	0,12	2	0,5	1
101136	F	94	SN	no data	Blood	21.11.2022	C	*stGM220.0*	0,015	0,015	0,015	0,5	256	256	4	0,5	1
101103	M	93	SL	Erysipel	Blood	18.11.2022	G	*stG485.0*	0,015	0,015	0,015	0,5	0,12	0,12	4	0,5	1
101102	M	84	BY	Sepsis	Blood	21.11.2022	G	*stG643.0*	0,015	0,03	0,03	0,5	0,12	0,12	4	64	1
101075	F	47	NW	no data	Blood	13.11.2022	G	*stG245.0*	0,015	0,015	0,015	0,5	0,12	0,12	4	8	0,5
101071	F	86	HE	no data	Blood	16.11.2022	G	*stC1400.0*	0,015	0,015	0,03	0,5	4	0,12	4	0,25	0,5
101026	M	83	SH	no data	Blood	13.11.2022	G	*stC74a.0*	0,015	0,03	0,06	0,5	4	0,12	4	0,5	1
101024	M	64	SN	Sepsis, Pneumonie	Blood	10.11.2022	G	*stC74a.0*	0,015	0,03	0,03	0,5	0,12	1	4	64	1
101020	M	89	NW	Sepsis	Blood	16.11.2022	G	*stG2078.0*	0,015	0,03	0,03	1	0,12	0,12	4	0,5	2
101016	M	79	NW	Sepsis	Blood	16.11.2022	G	*stC5345.0*	0,015	0,03	0,06	0,5	0,12	0,12	4	0,5	1
101002	M	78	SN	Sepsis	Blood	15.11.2022	G	*stC74a.0*	0,015	0,06	0,03	0,5	4	0,12	4	0,5	1
100982	M	63	BW	Sepsis	Blood	13.11.2022	G	*stC74a.0*	0,015	0,03	0,03	0,5	0,12	0,12	4	0,5	1
100938	M	88	MV	Fieber	Blood	10.11.2022	C	*stG62647.0*	0,015	0,03	0,03	0,5	0,12	0,12	4	0,5	1
100932	F	80	MV	no data	Blood	13.11.2022	C	*stG62647.0*	0,015	0,03	0,03	0,5	0,12	0,12	4	0,5	1
100931	M	86	MV	Sepsis	Blood	11.11.2022	G	*stG485.0*	0,015	0,03	0,03	0,5	0,12	0,12	4	4	0,5
100919	M	75	NW	no data	Blood	08.11.2022	C	*stG62647.0*	0,015	0,015	0,015	0,5	0,12	0,12	4	0,5	0,5
100910	F	78	SN	V. a. Urosepsis	Blood	05.11.2022	G	*stG245.1*	0,015	0,03	0,03	0,5	0,12	0,12	2	0,25	1
100909	M	90	SN	Infekt Kniegelenk	Gelenkpunktat	11.11.2022	G	*stG480.0*	0,015	0,03	0,03	0,5	0,12	0,12	4	0,5	1
100880	F	77	SH	Erysipel	Blood	06.11.2022	C	*stG62647.0*	0,015	0,03	0,03	0,5	0,12	0,12	4	0,5	1
100837	M	77	SH	no data	Blood	06.11.2022	C	*stG62647.0*	0,015	0,03	0,03	0,5	0,12	0,12	2	0,25	1
100832	M	31	HE	no data	Blood	04.11.2022	C	*stG62647.0*	0,015	0,03	0,03	0,5	0,12	0,12	4	0,5	1
100820	F	76		Erysipel	Blood	08.11.2022	C	*stG62647.0*	0,015	0,03	0,03	0,5	0,12	0,12	4	0,5	1
100817	F	65	NW	no data	Blood	07.11.2022	C	*stG62647.0*	0,015	0,03	0,03	0,5	0,12	0,12	4	0,5	1
100769	M	87	RP	Erysipel	Blood	28.10.2022	C	*stG62647.0*	0,015	0,03	0,03	0,5	0,12	0,12	4	0,5	1
100763	F	86	MV	Ulcus	Blood	02.11.2022	G	*stG2078.0*	0,015	0,015	0,03	0,5	256	16	4	0,5	1
100725	F	86	HE	Sepsis	Blood	31.10.2022	G	*stC1400.0*	0,015	0,03	0,03	0,5	8	0,12	4	0,25	0,5
100682	F	86	SH	Sepsis	Blood	27.10.2022	C	*stG62647.0*	0,015	0,03	0,03	0,5	0,12	0,12	2	0,25	1

100650	M	65	SH	Sepsis, Erysipel	Blood	22.10.2022		n/a	0,015	0,015	0,015	0,5	0,12	0,12	4	0,5	1
100643	M	53	RP	Sepsis, Erysipel	Blood	24.10.2022	C	*stG62647.0*	0,015	0,03	0,03	0,5	0,12	0,12	4	0,5	1
100620	F	42	RP	Sepsis	Blood	21.10.2022	G	*stG245.0*	0,015	0,015	0,06	0,5	0,12	0,12	4	16	0,5
100617	F	88	MV	no data	Blood	23.10.2022	G	*stG6.1*	0,015	0,015	0,015	0,5	0,12	0,12	4	0,25	0,5
100616	F	84	MV	no data	Blood	23.10.2022	G	*stG485.0*	0,015	0,015	0,015	0,5	0,12	0,12	4	0,5	0,5
100588	M	53	NW	no data	Blood	24.10.2022	C	*stG62647.0*	0,015	0,03	0,03	0,5	4	0,25	4	0,25	1
100582	n/a	101	NI	no data	Blood	22.10.2022	G	*stC74a.0*	0,015	0,03	0,03	0,5	4	1	4	4	1
100548	M	80	NW	Erysipel, Pneumonie	Blood	21.10.2022	C	*stG62647.0*	0,015	0,03	0,03	0,5	0,12	0,12	2	0,25	1
100538	F	79	BY	Sepsis	Blood	19.10.2022	C	*stG62647.0*	0,015	0,015	0,03	0,5	0,12	0,12	4	0,5	1
100530	F	52	RP	Sepsis	Blood	18.10.2022	C	*stG62647.0*	0,015	0,03	0,03	0,5	0,12	0,12	4	0,25	1
100518	M	57	MV	no data	Blood	16.10.2022	G	*stC74a.0*	0,015	0,015	0,015	0,5	0,12	0,12	4	8	0,5
100473	F	70	HE	no data	Blood	14.10.2022	G	*stG11.0*	0,015	0,03	0,03	0,5	0,12	0,12	4	0,5	1
100459	F	41	NW	no data	Blood	16.10.2022	C	*stG62647.0*	0,015	0,03	0,03	0,5	0,12	0,12	4	0,5	1
100224	M	89	SN	no data	Blood	02.10.2022	G	*stC74a.0*	0,015	0,03	0,03	0,5	4	0,12	4	1	1
100184	M	57	HE	Sepsis, septischer Schock	Blood	25.09.2022	G	*stG6.1*	0,015	0,015	0,015	0,5	4	0,12	4	0,5	1
100150	F	50	NI	Sepsis, Abszess, Empyem	Blood	28.09.2022	C	*stG62647.0*	0,015	0,03	0,015	1	0,12	0,12	4	0,5	1
100138	M	79	MV	no data	Blood	30.09.2022	G	*stG652.0*	0,015	0,015	0,03	0,5	0,12	0,12	4	32	1
100058	F	84	SH	Sepsis	Blood	20.09.2022	C	*stG2574.3*	0,015	0,015	0,015	0,5	0,12	0,12	4	0,5	1
100006	M	63	RP	Erysipel, Sepsis	Blood	19.09.2022	G	*stG840.2*	0,015	0,015	0,015	0,5	1	0,12	4	64	1
100006	M	63	RP	Erysipel, Sepsis	Blood	19.09.2022	G	*stG840.2*	0,015	0,015	0,015	0,5	1	0,12	4	64	1
99996	M	69	NW	Erysipel	Blood	18.09.2022	C	*stG2574.3*	0,015	0,03	0,03	0,5	0,12	0,12	4	0,5	0,5
99993	F	76	NW	no data	Blood	09.09.2022	C	*stG62647.0*	0,015	0,015	0,015	1	0,12	0,12	4	0,5	2
99992	M	48	NW	Phlegmone	Blood	09.09.2022	G	*stG2574.3*	0,015	0,015	0,015	0,5	8	256	4	64	0,5
99975	M	72	SL	Erysipel	Blood	14.09.2022	G	*stG245.0*	0,015	0,03	0,03	0,5	0,12	0,12	4	0,5	0,5
99956	M	43	SN	no data	Blood	11.09.2022	G	*stC74a.9*	0,015	0,015	0,015	0,5	4	0,12	4	0,5	1
99955	M	70	SN	Phlegmone	Blood	14.09.2022	G	*stG485.0*	0,015	0,03	0,03	0,5	0,12	0,12	4	0,5	1
99952	M	87	NW	Sepsis	Blood	15.09.2022	G	*stC74a.0*	0,015	0,015	0,03	0,5	0,12	0,12	4	4	0,5
99950	M	96	MV	Sepsis	Blood	16.09.2022	C	*stG62647.0*	0,015	0,015	0,015	0,5	4	1	4	0,25	1
99933	F	87	SN	Sepsis, Arthritis	Blood	12.09.2022	C	*stG62647.13*	0,015	0,015	0,015	0,5	0,12	0,12	4	0,5	1
99931	F	97	SN	Sepsis, Erysipel	Blood	15.09.2022	C	*stG62647.11*	0,015	0,015	0,03	0,5	0,12	0,12	8	0,5	1
99922	M	76	NW	Sepsis	Blood	11.09.2022	C	*stG62647.0*	0,015	0,03	0,03	0,5	0,12	0,12	4	0,5	1
99910	F	88	NW	Sepsis	Blood	12.09.2022	C	*stG62647.0*	0,015	0,015	0,03	0,5	0,12	0,12	4	0,25	1
99859	M	93	BW	Sepsis, Phlegmone	Blood	29.08.2022	C	*stG62647.0*	0,015	0,03	0,03	0,5	0,12	0,12	4	0,5	0,5
99843	M	74	RP	Sepsis	Blood	03.09.2022	G	*stG10.0*	0,015	0,015	0,03	0,5	0,12	0,12	4	1	1
99812	M	83	SH	Endokarditis	Blood	01.09.2022	G	*stG485.0*	0,015	0,03	0,015	0,5	0,12	0,12	4	8	1
99802	M	80	MV	no data	Blood	31.08.2022	G	*stC74a.0*	0,015	0,015	0,015	0,5	2	0,12	4	4	1
99794	F	90	SN	Sepsis	Blood	25.08.2022	G	*stG6.1*	0,015	0,015	0,015	0,5	0,12	0,12	2	32	0,5
99765	F	65	BY	Erysipel Unterschenkel	Blood	18.08.2022	G	*stG6.1*	0,015	0,03	0,03	0,5	4	0,12	4	0,5	0,5
99761	M	75	HE	no data	Blood	22.08.2022	G	*stC1400.0*	0,015	0,015	0,015	0,5	2	0,12	4	0,25	0,5
99757	M	71	NW	Sepsis	Blood	24.08.2022	G	*stG840.0*	0,015	0,015	0,015	0,5	256	64	4	64	1
99756	F	80	NW	Sepsis	Blood	26.08.2022	C	*stG62647.0*	0,015	0,015	0,03	0,5	0,12	0,12	4	0,5	1
99750	F	66	HE	no data	Blood	22.08.2022	G	*stG643.0*	0,015	0,015	0,03	0,5	0,12	0,12	4	32	1
99737	M	92	NW	no data	Blood	16.08.2022	G	*stG480.0*	0,015	0,015	0,015	0,5	0,12	0,12	2	0,25	1
99736	M	89	NW	no data	Blood	16.08.2022	G	*stG840.0*	0,015	0,03	0,03	0,5	0,12	0,12	4	0,5	1
99735	M	89	NW	no data	Blood	15.08.2022	C	*stG62647.0*	0,015	0,015	0,015	0,5	0,12	0,12	4	0,5	1
99733	F	78	SH	HWI	Blood	21.08.2022	G	*stC74a.0*	0,015	0,03	0,015	0,5	2	0,12	4	4	1
99728	M	83	BY	Sepsis	Blood	23.08.2022	G	*stG6792.0*	0,015	0,015	0,03	0,5	0,12	0,12	4	0,25	1
99705	M	80	RP	Sepsis	Blood	23.08.2022	A	*stG652.0*	0,015	0,015	0,015	0,5	0,12	0,12	4	8	1
99699	M	63	BW	Sepsis	Blood	17.08.2022	G	*stG6.1*	0,015	0,015	0,015	0,5	4	0,12	2	0,5	1
99697	n/a	68	BW	Sepsis	Blood	13.08.2022	G	*stG480.0*	0,015	0,015	0,015	0,5	256	0,25	4	32	1
99692	M	84	SH	Pneumonie, Erysipel	Blood	18.08.2022	C	*stG62647.0*	0,015	0,015	0,03	0,5	256	256	4	0,5	1
99687	F	94	NI	Sepsis	Blood	15.08.2022	G	*stG485.0*	0,015	0,015	0,03	0,5	4	0,12	4	4	0,5

99670	F	62	NW	no data	Blood	15.08.2022	C	*stG62647.0*	0,015	0,03	0,03	0,5	8	0,5	4	0,5	2
99669	M	66	NW	no data	Blood	14.08.2022	G	*stG485.0*	0,015	0,03	0,03	0,5	0,12	0,12	4	8	0,5
99656	M	80	BW	Sepsis	Blood	14.08.2022	C	*stG62647.0*	0,015	0,03	0,03	0,5	0,12	0,12	4	0,5	1
99629	n/a	90	NI	no data	Blood	26.07.2022	G	*stG652.0*	0,015	0,03	0,03	0,5	0,12	0,12	4	0,5	0,5
99606	M	85	SN	Sepsis	Blood	09.08.2022	C	*stG62647.0*	0,015	0,03	0,03	0,5	0,12	0,12	2	0,5	1
99593	M	64	RP	Erysipel	Blood	06.08.2022	C	*stG62647.0*	0,015	0,03	0,03	0,5	0,12	0,12	4	0,5	1
99573	M	89	RP	Sepsis	Blood	09.08.2022	A	*stG485.0*	0,015	0,015	0,015	0,5	0,12	0,12	4	8	2
99569	F	63	SH	Erysipel	Blood	06.08.2022	C	*stC839.0*	0,015	0,03	0,03	0,5	32	0,12	4	64	1
99562	M	70	RP	no data	Blood	30.07.2022	G	*stG120.0*	0,015	0,015	0,03	0,5	0,12	0,12	4	4	0,5
99520	M	79	NW	Sepsis	Blood	31.07.2022	G	*stG485.0*	0,015	0,03	0,03	0,5	4	0,12	4	0,5	1
99518	F	29	NW	Sepsis, Peritonitis	Blood	31.07.2022	G	*stG643.0*	0,015	0,03	0,03	0,5	0,12	0,12	4	0,5	1
99514	M	85	RP	Erysipel	Blood	29.07.2022	G	*stG245.0*	0,015	0,03	0,03	0,5	4	0,12	4	0,5	0,5
99424	F	65	BY	Sepsis	Blood	21.07.2022	C	*stG62647.0*	0,015	0,03	0,03	0,5	0,12	0,12	2	0,5	1
99417	F	97	NW	Sepsis	Blood	17.07.2022	G	*stG245.0*	0,015	0,03	0,03	0,5	0,12	0,12	4	0,5	0,5
99401	M	43	SN	Sepsis	Blood	17.07.2022	G	*stC74a.0*	0,015	0,03	0,03	0,5	0,12	0,12	4	8	1
99393	F	75	BY	no data	Blood	17.07.2022	G	*stG10.0*	0,015	0,03	0,03	0,5	0,12	0,12	4	16	1
99381	M	77	NW	Erysipel	Blood	05.07.2022	G	*stG10.0*	0,015	0,03	0,03	0,5	0,12	0,12	4	64	1
99376	M	80	NW	Erysipel, Phlegmone	Blood	09.07.2022	G	*stGLP1.2*	0,015	0,015	0,03	0,5	256	256	4	64	1
99348	F	86	SN	no data	Blood	12.07.2022	G	*stC74a.9*	0,015	0,015	0,015	0,5	0,12	0,12	4	8	1
99337	M	76	SH	Pneumonie	Blood	15.07.2022	C	*stG2574.3*	0,015	0,03	0,03	0,5	0,12	0,12	4	0,5	1
99331	M	58	NW	Sepsis	Blood	12.07.2022	C	*stG62647.0*	0,015	0,03	0,03	0,5	0,12	0,12	4	0,5	0,5
99326	M	79	RP	Phlegmone	Blood	13.07.2022	C	*stG62647.0*	0,015	0,03	0,03	0,5	0,12	0,12	4	0,5	1
99325	F	59	RP	Sepsis	Blood	12.07.2022	C	*stG62647.0*	0,015	0,03	0,03	0,5	0,12	0,12	4	0,5	1
99321	F	83	NW	Sepsis	Blood	12.07.2022	G	*stG480.0*	0,015	0,015	0,03	0,5	0,12	0,12	4	0,5	1
99315	F	59	NW	Sepsis	Blood	10.07.2022	G	*stG485.0*	0,015	0,015	0,03	0,5	0,12	0,12	4	1	1
99259	M	62	NW	Sepsis	Blood	02.07.2022	G	*stG485.0*	0,015	0,03	0,03	0,5	0,12	0,12	4	64	1
99247	M	12	BY	chronische Bursitis	Biopsie	02.07.2022	C	*stG62647.0*	0,015	0,015	0,03	0,5	0,12	0,12	4	0,25	1
99245	M	90	BW	Sepsis	Blood	03.07.2022	G	*stG485.0*	0,015	0,03	0,03	0,5	0,12	0,12	4	4	1
99233	M	81	NW	Sepsis	Blood	04.02.2022	G	*stC74a.9*	0,015	0,03	0,03	0,5	256	256	4	0,5	1
99228	F	68	BY	Ulzerationen	Swab wound	01.07.2022	G	*stG6.1*	0,015	0,03	0,03	0,5	4	0,12	4	0,5	0,5
99216	F	1	RP	no data	Blood	28.06.2022	C	*stG62647.0*	0,015	0,03	0,03	0,5	0,12	0,12	2	0,5	1
99174	M	87	HE	Sepsis	Blood	24.06.2022	G	*stG480.0*	0,015	0,03	0,03	0,5	0,12	0,12	4	0,5	2
99169	M	54	NW	no data	Blood	17.06.2022	C	*stG643.0*	0,015	0,03	0,03	0,5	0,12	0,12	4	0,5	1
99158	n/a	78	BY	no data	Blood	24.06.2022	C	*stG62647.0*	0,015	0,03	0,03	0,5	0,12	0,12	4	0,5	1
99137	F	84	NW	Sepsis	Blood	21.06.2022	G	*stC74a.0*	0,015	0,03	0,03	0,5	0,12	0,12	4	0,5	1
99129	M	62	NW	no data	Blood	14.06.2022	C	*stG62647.0*	0,015	0,03	0,03	0,5	0,12	0,12	2	0,5	1
99117	M	69	BY	Erysipel Unterschenkel	Swab wound	18.06.2022	G	*stG480.0*	0,015	0,03	0,03	0,5	0,12	0,12	4	0,5	1
99068	M	91	SN	Erysipel	Blood	14.06.2022	G	*stC46.0*	0,015	0,015	0,015	0,5	0,12	0,12	4	0,5	1
99021	M	40	BY	Kniegelenkempyem	Swab Kniegelenk	10.06.2022	C	*stG62647.0*	0,015	0,03	0,03	0,5	0,12	0,12	4	0,5	1
99020	M	63	BY	Sepsis, infizierte Lymphozele	Blood	12.06.2022	G	*stG2078.0*	0,015	0,03	0,03	0,5	0,12	0,12	4	0,5	1
99015	F	84	NW	Harnwegsinfekt	Blood	11.06.2022	G	*stG10.13*	0,015	0,03	0,03	0,5	0,12	0,12	4	64	1
99003	F	81	SH	Phlegmone	Blood	11.06.2022	G	*stG10.0*	0,03	0,03	0,03	0,5	0,12	0,12	2	64	1
98986	M	79	HE	Sepsis	Blood	05.06.2022	C	*stG62647.0*	0,015	0,03	0,03	0,5	8	0,25	4	1	1
98975	M	67	SH	Aneurysma	Blood	10.06.2022	C	*stG62647.0*	0,015	0,03	0,03	0,5	8	0,12	4	0,5	1
98972	M	71	BY	no data	Blood	05.06.2022	G	*stC5345.0*	0,015	0,03	0,03	0,5	0,12	0,12	4	0,5	1
98963	F	86	RP	sept. Arthritis	Blood	02.06.2022	G	*stG6.1*	0,015	0,03	0,03	0,5	0,12	0,12	2	0,5	0,5
98960	F	73	SN	Infektion am Arm	Blood	08.06.2022	C	*stG62647.0*	0,015	0,03	0,03	0,5	0,12	0,12	4	0,5	1
98950	F	94	HE	Sepsis	Blood	04.06.2022	C	*stG62647.0*	0,015	0,03	0,03	0,5	0,12	0,12	4	0,5	1
98871	F	81	SN	Sepsis	Blood	02.06.2022	G	*stG6.1*	0,015	0,03	0,03	0,5	0,12	0,12	4	0,5	0,5
98870	F	78	SN	Sepsis	Blood	03.06.2022	G	*stG245.1*	0,015	0,03	0,03	0,5	0,12	0,12	4	0,5	0,5
98849	M	68	HE	unklarer Infekt	Blood	27.05.2022	G	*stG245.0*	0,03	0,06	0,06	0,5	256	256	4	0,5	0,5
98798	M	90	SL	Sepsis	Blood	18.06.2022	G	*stC74a.0*	0,015	0,03	0,03	0,5	0,12	0,12	4	0,5	1

98768	F	85	HE	no data	Gelenkpunktat	18.05.2022	C	*stG62647.0*	0,015	0,03	0,03	0,5	0,12	0,12	4	0,5	1
98764	M	78	NW	Phlegmone	Blood	17.05.2022	C	*stC839.0*	0,015	0,015	0,015	0,5	256	256	8	0,5	1
98752	M	76	HE	no data	Blood	15.05.2022	G	*stG652.24*	0,015	0,03	0,03	0,5	0,12	0,12	4	0,5	1
98735	M	79	NW	no data	Blood	18.05.2022	G	*stC74a.0*	0,015	0,03	0,03	0,5	0,12	0,12	4	0,5	1
98726	M	87	SH	Urosepsis	Blood	20.05.2022	G	*stG34.0*	0,015	0,03	0,015	0,5	0,12	0,12	2	0,5	1
98712	M	79	SH	Pneumonie	Blood	19.05.2022	C	*stG4545.0*	0,015	0,015	0,015	0,5	0,12	0,12	4	2	0,5
98698	M	68	NW	no data	Blood	17.05.2022	C	*stG62647.0*	0,015	0,03	0,03	0,5	0,12	0,12	4	0,5	0,5
98684	M	74	SH	Infekt Hüft TEP	Blood	16.05.2022	G	*stC74a.0*	0,015	0,03	0,03	0,5	8	0,12	4	1	1
98683	M	77	SH	Sepsis	Blood	17.05.2022	C	*stG62647.0*	0,015	0,03	0,03	0,5	0,12	0,12	4	0,5	1
98676	M	85	SN	Fieber	Blood	19.05.2022	C	*stG62647.0*	0,015	0,03	0,03	0,5	4	0,25	4	0,5	1
98674	M	78	SN	Schulterempyem	Gelenkpunktat	17.05.2022	C	*stG62647.0*	0,015	0,03	0,03	0,5	0,12	0,12	4	0,5	1
98607	M	48	HE	no data	Blood	09.05.2022	C	*stG62647.0*	0,015	0,06	0,06	0,5	0,12	0,12	4	0,5	1
98598	M	80	SN	Sepsis, Endokarditis	Blood	09.05.2022	G	*stG2078.0*	0,015	0,03	0,03	0,5	0,12	0,12	4	0,5	1
98594	M	83	NW	Sepsis, Erysipel	Blood	12.05.2022	C	*stG62647.0*	0,015	0,03	0,03	0,5	0,12	0,12	4	0,5	1
98588	M	35	NW	no data	Blood	09.05.2022	G	*stG2574.3*	0,015	0,015	0,03	0,5	64	256	8	64	2
98554	M	67	BW	Sepsis	Blood	29.04.2022	G	*stC74a.0*	0,015	0,03	0,03	0,5	4	0,12	4	0,5	0,5
98548	M	60	BW	Sepsis	Blood	28.04.2022	G	*stG485.0*	0,015	0,03	0,03	0,5	0,12	0,12	4	32	1
98527	M	50	SH	Wunde am Fuß	Blood	05.05.2022	G	*stC74a.11*	0,015	0,03	0,03	0,5	4	0,12	4	0,25	0,5
98526	M	59	SH	Erysipel	Blood	04.05.2022	C	*stG62647.0*	0,015	0,03	0,03	0,5	0,12	0,12	4	0,5	1
98506	M	78	BY	Sepsis	Blood	03.05.2022	L	n/a	0,015	0,015	0,015	0,5	0,12	0,12	2	0,25	1
98495	M	74	SN	Fieber unklarer Genese	Blood	03.05.2022	C	*stG62647.0*	0,015	0,03	0,03	0,5	0,12	0,12	2	0,25	1
98487	M	70	SH	Pneumonie	Blood	02.05.2022	G	*stG485.0*	0,015	0,03	0,03	0,5	0,12	0,12	4	4	0,5
98472	M	57	HE	Sepsis	Blood	28.04.2022	G	*stG6.0*	0,015	0,015	0,015	0,5	0,12	0,12	4	32	1
98471	M	72	HE	Sepsis	Blood	29.04.2022	G	*stG485.0*	0,015	0,03	0,03	0,5	0,12	0,12	4	4	1
98462	M	86	SH	Stauungspneumonie	Blood	30.04.2022	G	*stC74a.0*	0,015	0,03	0,03	0,5	0,12	0,12	4	0,5	1
98441	F	86	RP	Erysipel	Blood	06.05.2022	G	*stC74a.0*	0,015	0,03	0,03	0,5	0,12	0,12	4	8	1
98420	M	74	SH	Erysipel	Blood	29.04.2022	C	*stG62647.0*	0,015	0,015	0,015	0,5	0,12	0,12	2	0,25	2
98393	M	48	SN	Handgelenksempyem	Gelenkpunktat	24.04.2022	G	*stG485.0*	0,015	0,03	0,03	0,5	0,12	0,12	4	1	2
98379	F	79	SH	Pneumonie	Blood	24.04.2022	G	*stG2078.0*	0,015	0,03	0,03	0,5	0,12	0,12	4	0,5	1
98378	M	77	RP	Sepsis	Blood	21.04.2022	C	*stG62647.0*	0,015	0,03	0,03	0,5	0,12	0,12	4	0,5	1
98348	M	87	BY	Infekt unklarer Genese	Blood	19.04.2022	G	*stG485.0*	0,015	0,03	0,03	0,5	0,12	0,12	4	1	1
98337	M	82	SH	no data	Blood	20.04.2022	C	*stG62647.4*	0,015	0,03	0,03	0,5	0,12	0,12	4	0,5	1
98336	M	60	SH	no data	Puncture Ascites	19.04.2022	C	*stG62647.0*	0,015	0,03	0,03	0,5	0,12	0,12	2	0,5	1
98312	M	88	NW	no data	Blood	24.04.2022	C	*stG2078.15*	0,015	0,015	0,015	0,5	2	256	2	0,5	1
98302	M	85	NW	Sepsis	Blood	18.04.2022	G	*stG485.0*	0,015	0,12	0,03	0,5	0,12	0,12	2	0,5	1
98266	F	94	SN	no data	Blood	12.04.2022	G	*stC839.2*	0,015	0,03	0,06	0,5	0,12	0,12	4	64	1
98264	F	88	SH	Pneumonie	Blood	16.04.2022	G	*stG485.0*	0,015	0,015	0,03	0,5	256	0,12	4	0,5	2
98261	F	73	SH	Pneumonie	Blood	14.04.2022	G	*stG2078.0*	0,015	0,015	0,015	0,5	0,12	0,12	4	0,5	1
98204	F	75	NW	no data	Blood	11.04.2022	C	*stG62647.0*	0,015	0,015	0,03	0,5	0,12	0,12	4	0,5	0,5
98189	F	80	RP	Sepsis	Blood	07.04.2022	C	*stG2574.3*	0,015	0,03	0,03	0,5	0,12	0,12	4	0,5	1
98142	M	87	SH	no data	Blood	09.04.2022	C	*stG62647.0*	0,03	0,03	0,03	0,5	0,12	0,12	4	0,5	1
98141	M	50	BW	Sepsis, Wundinfektion	Blood	04.04.2022	C	*stG62647.0*	0,015	0,03	0,03	0,5	256	256	4	32	1
98132	M	59	NW	Sepsis	Blood	08.04.2022	G	*stC74a.0*	0,015	0,03	0,03	0,5	4	0,12	4	0,5	0,5
98125	F	72	NW	Sepsis	Blood	08.04.2022	C	*stG62647.0*	0,015	0,03	0,03	0,5	0,12	0,12	4	0,5	1
98081	F	75	SH	Meningitis	Blood	01.04.2022	C	*stG2574.3*	0,015	0,03	0,03	0,5	0,12	0,12	4	0,5	1
98074	M	71	NW	no data	Blood	03.04.2022	C	*stG62647.0*	0,015	0,03	0,03	0,5	0,12	0,12	4	0,5	1
98049	M	78	BY	no data	Blood	30.03.2022	C	*stG62647.0*	0,015	0,03	0,03	0,5	0,12	0,12	4	0,5	1
98048	M	62	BY	Erysipel	Blood	30.03.2022	C	*stG62647.0*	0,015	0,015	0,03	0,5	0,12	0,12	4	0,5	1
98007	M	55	SH	no data	Blood	27.03.2022	C	*stG62647.0*	0,015	0,015	0,03	0,5	0,12	0,12	2	0,5	1
98005	M	74	NW	Sepsis	Blood	27.03.2022	C	*stG62647.0*	0,015	0,03	0,03	0,5	0,12	0,12	4	0,5	0,5
97968	F	56	SN	Fieber unklarer Genese	Blood	23.03.2022	G	*stG245.0*	0,015	0,03	0,03	0,5	4	0,12	4	0,5	1
97965	M	80	NW	Pneumonie	Blood	26.03.2022	C	*stG62647.0*	0,015	0,03	0,03	0,5	0,12	0,12	4	0,5	1

97955	F	1	NW	Sepsis	Blood	23.03.2022	C	*stG62647.0*	0,015	0,03	0,03	0,5	0,12	0,12	2	0,5	0,5
97949	F	59	SH	no data	Blood	21.03.2022	C	*stG2574.3*	0,03	0,015	0,015	0,5	0,12	0,12	4	0,5	1
97935	F	61	SH	Sepsis	Blood	21.03.2022	G	*stC74a.0*	0,015	0,015	0,015	0,5	0,12	0,015	4	8	1
97924	F	50	NW	Sepsis	Blood	20.03.2022	G	*stG485.0*	0,015	0,03	0,03	1	0,12	0,12	4	0,5	2
97888	M	79	SH	Erysipel	Blood	17.03.2022	C	*stG62647.0*	0,015	0,03	0,03	0,5	0,12	0,12	4	0,5	0,5
97834	M	67	RP	Sepsis	Blood	10.03.2022	C	*stG62647.0*	0,015	0,03	0,03	0,5	2	1	4	0,5	1
97830	M	82	NW	Stauungsdermatitis	Blood	16.03.2022	G	*stG2078.0*	0,015	0,015	0,015	0,5	0,12	0,12	4	1	2
97797	M	58	SH	no data	Blood	08.03.2022	G	*stG652.0*	0,015	0,015	0,015	0,5	0,12	0,12	4	32	0,5
97794	F	88	SH	Erysipel	Blood	10.03.2022	G	*stG480.0*	0,015	0,015	0,015	0,5	0,12	0,12	4	0,5	1
97768	F	87	HE	no data	Blood	04.03.2022	C	*stC6979.0*	0,015	0,015	0,03	0,5	256	256	4	64	1
97748	F	87	NW	Fieber	Blood	08.03.2022	G	*stG2078.0*	0,015	0,03	0,03	0,5	0,12	0,12	4	0,5	0,5
97725	F	81	NW	no data	Blood	03.03.2022	C	*stG62647.0*	0,015	0,03	0,03	0,5	0,12	0,12	4	0,5	1
97724	M	80	NW	no data	Blood	03.03.2022	G	*stC5345.0*	0,015	0,03	0,03	0,5	0,12	0,12	4	0,5	1
97718	M	77	SN	no data	Gelenkpunktat	04.03.2022	G	*stG485.0*	0,015	0,03	0,03	0,5	4	0,12	4	4	1
97717	M	85	SN	FKO	Blood	01.03.2022	C	*stG62647.0*	0,015	0,03	0,03	0,5	0,12	0,12	4	0,5	1
97703	M	48	SN	Fieber	Blood	26.02.2022	C	*stG62647.0*	0,015	0,015	0,015	0,5	0,12	0,12	4	0,5	1
97680	M	87	SH	Sepsis	Blood	26.02.2022	G	*stG10.0*	0,015	0,03	0,03	0,5	0,12	0,12	4	8	1
97665	M	89	SN	no data	Blood	22.02.2022	G	*stG6.1*	0,015	0,03	0,03	0,5	0,12	0,12	4	0,5	0,5
97656	M	91	NW	Sepsis	Blood	23.02.2022	C	*stG62647.0*	0,015	0,015	0,03	0,5	8	0,5	4	0,25	1
97655	M	59	NW	Sepsis	Blood	25.02.2022	C	*stG62647.0*	0,015	0,03	0,03	0,5	0,12	0,12	4	0,5	1
97654	M	73	BY	Ulzerationen	Swab wound	16.02.2022	G	*stG10.0*	0,015	0,03	0,03	0,5	0,12	0,12	4	64	1
97578	M	55	NW	no data	Blood	18.02.2022	G	*stG485.0*	0,015	0,03	0,03	0,5	0,12	0,12	16	4	0,5
97549	F	94	HE	Erysipel	Blood	11.02.2022	G	*stC74a.0*	0,015	0,03	0,03	0,5	4	0,12	4	0,5	0,5
97457	F	90	BW	Erysipel	Blood	04.02.2022	C	*stG62647.0*	0,015	0,03	0,03	0,5	0,12	0,12	4	0,5	1
97450	M	58	SH	Phlegmone, chron. Unterschenkelulcus	Blood	01.02.2022	C	*stG62647.0*	0,015	0,03	0,03	0,5	0,12	0,12	4	0,5	1
97417	M	70	HE	Erysipel	Blood	30.01.2022	G	*stG2078.0*	0,015	0,03	0,03	0,5	0,12	0,12	4	1	1
97402	M	68	RP	Sepsis	Blood	29.01.2022	G	*stC74a.0*	0,015	0,03	0,03	0,5	0,12	0,12	4	8	1
97371	M	73	SH	Harnwegsinfekt, COVID-19	Blood	28.01.2022	G	*stG10.0*	0,015	0,03	0,03	0,5	0,12	0,12	4	64	1
97370	M	77	SH	Z.n. Zehenamputation	Blood	28.01.2022	G	*stG643.0*	0,015	0,03	0,03	0,5	16	0,12	4	64	1
97292	M	86	SH	Pneumonie	Blood	16.01.2022	C	*stG62647.0*	0,015	0,03	0,03	0,5	8	0,25	4	0,5	1
97235	M	87	RP	Sepsis	Blood	13.01.2022	G	*stG652.0*	0,015	0,03	0,03	0,5	0,12	0,12	8	1	1
97202	F	85	NW	Sepsis	Blood	08.01.2022	G	*stC74a.0*	0,015	0,03	0,03	0,5	4	0,12	4	1	0,5
97196	F	81	SH	Pneumonie, Erysipel	Blood	09.01.2022	G	*stG2078.0*	0,015	0,03	0,03	0,5	0,12	0,12	4	1	1
97140	M	60	SN	Fieber	Blood	06.01.2022	G	*stG11.0*	0,03	0,03	0,12	1	256	128	4	32	2
97079	M	44	RP	Erysipel, Sepsis	Blood	25.12.2021	G	*stG485.0*	0,015	0,03	0,06	0,5	0,12	0,12	4	8	0,5
97026	M	82	HE	sept. Arthritis	Puncture Gelenk	28.12.2021	C	*stG62647.0*	0,015	0,03	0,03	0,5	0,12	0,12	4	1	1
97023	F	62	BW	Fieber unklarer Ursache	Blood	29.12.2021	G	*stG11.0*	0,015	0,03	0,03	0,5	256	128	4	64	1
96994	M	69	NW	no data	Blood	26.12.2021	G	*stC74a.0*	0,06	0,03	0,03	0,5	8	32	4	2	2
96938	M	86	NW	Sepsis	Blood	24.12.2021	C	*stG62647.0*	0,03	0,03	0,03	0,5	0,12	0,12	4	1	0,25
96928	F	82	SH	chron. Wundinfekt	Blood	17.12.2021	C	*stG62647.0*	0,015	0,03	0,03	0,5	32	32	8	0,5	1
96906	F	69	RP	no data	Blood	12.12.2021	C	*stG62647.0*	0,015	0,03	0,03	0,5	0,12	0,12	4	0,5	1
96852	M	81	SN	Infekt Knie	Gelenkpunktat	16.12.2021	G	*stC74a.0*	0,015	0,03	0,03	0,5	0,12	0,12	4	0,5	1
96823	M	52	NI	Erysipel	Blood	12.12.2021	C	*stG62647.0*	0,015	0,12	0,03	0,5	0,12	0,25	4	0,5	1
96822	F	95	NW	no data	Blood	10.12.2021	G	*stG480.0*	0,015	0,015	0,015	0,5	2	0,12	4	32	0,5
96775	M	69	NW	no data	Blood	09.12.2021	G	*stGLP1.0*	0,015	0,015	0,015	0,5	0,12	0,12	4	64	1
96757	M	66	NW	no data	Blood	11.12.2021	C	*stG62647.0*	0,015	0,03	0,03	0,5	0,12	0,12	4	0,5	1
96734	F	75	NW	Erysipel	Blood	06.12.2021	C	*stG62647.0*	0,015	0,03	0,03	0,5	0,12	0,12	4	0,5	2
96704	F	83	SN	Sepsis	Blood	30.11.2021	G	*stC74a.0*	0,015	0,03	0,03	0,5	4	0,25	4	1	1
96683	M	79	SH	Stauungsdermatitis	Blood	30.11.2021	G	*stG652.5*	0,015	0,03	0,03	0,5	0,12	0,12	4	0,5	1
96658	F	48	BY	Wundinfektion	Swab wound	29.11.2021	G	*stC74a.12*	0,015	0,03	0,03	0,5	256	0,12	4	0,5	1
96644	F	76	SH	no data	Blood	29.11.2021	G	*stC74a.9*	0,015	0,03	0,03	0,5	0,12	0,12	4	4	1

96623	M	35	NI	no data	Blood	14.11.2021	A	*stG652.0*	0,015	0,03	0,03	0,5	0,12	0,12	4	8	1
96607	F	92	NW	Sepsis	Blood	30.11.2021	A	*stG652.0*	0,015	0,03	0,03	0,5	0,12	0,12	4	16	1
96604	M	77	SH	Harnwegsinfektion	Blood	25.11.2021	C	*stG62647.0*	0,015	0,03	0,03	0,5	0,12	0,12	4	1	0,5
96538	M	57	NW	no data	Blood	22.11.2021	C	*stG62647.0*	0,015	0,03	0,03	0,5	0,12	0,12	4	0,5	1
96525	M	63	NW	no data	Blood	21.11.2021	C	*stG2574.3*	0,015	0,03	0,03	0,5	0,12	0,12	4	1	1
96494	M	86	SH	Erysipel	Blood	15.11.2021	C	*stG62647.0*	0,015	0,03	0,06	0,5	0,12	0,12	4	0,5	0,5
96477	M	49	SN	Pneumonie	Blood	21.11.2021	G	*stG2078.0*	0,015	0,03	0,03	0,5	0,12	0,12	4	1	1
96460	M	59	NW	Sepsis	Blood	18.11.2021	G	*stGLP1.0*	0,015	0,015	0,015	0,5	0,12	0,12	4	64	2
96454	M	65	RP	Endokarditis	Blood	15.11.2021	C	*stG62647.0*	0,015	0,03	0,03	0,5	0,12	0,12	4	0,5	1
96435	M	72	RP	Erysipel	Blood	10.11.2021	G	*stG480.0*	0,015	0,03	0,03	0,5	0,12	0,12	4	0,5	2
96421	M	66	BE	Sepsis	Blood	12.11.2021	C	*stG62647.0*	0,015	0,03	0,03	0,5	0,12	0,12	4	0,5	1
96420	M	73	BE	COPD	Blood	08.11.2021	C	*stG62647.0*	0,015	0,03	0,03	0,5	0,12	0,12	4	0,5	1
96407	M	81	RP	sept. Arthritis	Blood	13.11.2021	C	*stG62647.0*	0,015	0,03	0,03	0,5	0,12	0,12	4	0,5	1
96401	M	50	SH	infizierte Wunde	Blood	15.11.2021	G	*stG6.3*	0,015	0,03	0,03	0,5	0,12	0,12	4	0,5	0,5
96394	F	54	SN	Sepsis	Blood	15.11.2021	C	*stG62647.0*	0,015	0,03	0,03	0,5	0,12	0,12	4	0,5	1
96371	F	87	NW	no data	Gelenkpunktat	11.11.2021	G	*stC74a.0*	0,015	0,03	0,03	0,5	4	0,12	4	0,5	2
96362	M	85	SH	Kardiogener Schock,<br>Herzinsuffizienz	Blood<br>Pleurapunktat	10.11.2021	C	*stG62647.0*	0,015	0,03	0,03	0,5	0,12	0,12	4	0,5	1
96312	M	69	RP	Sepsis, Erysipel	Blood	07.11.2021	G	*stC74a.0*	0,015	0,03	0,03	0,5	0,12	0,12	4	4	0,5
96302	F	83	SH	TVT	Blood	06.11.2021	G	*stG2078.1*	0,015	0,03	0,03	0,5	0,12	0,12	4	0,5	1
96276	M	84	NW	Sepsis	Blood	06.11.2021	G	*stG643.0*	0,015	0,03	0,03	0,5	16	256	4	16	1
96259	M	74	SH	sept. Arthritis	Gelenkpunktat	05.11.2021	G	*stC46.0*	0,015	0,03	0,03	0,5	0,12	0,12	4	0,5	1
96257	M	68	SN	acute exacerbation by<br>bronchitis, Sepsis	Blood	08.11.2021	C	*stG62647.0*	0,015	0,03	0,03	0,5	0,12	0,12	4	0,5	1
96231	M	77	NW	Sepsis	Blood	04.11.2021	G	*stG485.0*	0,015	0,03	0,03	0,5	0,12	0,12	4	64	1
96222	M	57	SH	Erysipel	Blood	02.01.2021	G	*stG652.0*	0,015	0,03	0,03	0,5	0,12	0,12	4	0,5	1
96216	M	76	SN	Bakteriämie	Blood	02.11.2021	C	*stG62647.0*	0,015	0,03	0,03	0,5	0,12	0,12	4	0,5	1
96166	M	82	SL	Erysipel	Blood	26.10.2021	G	*stG245.0*	0,015	0,03	0,03	0,5	0,12	0,12	4	16	0,5
96126	M	88	SN	Sepsis	Blood	29.10.2021	G	*stG485.0*	0,015	0,03	0,03	0,5	0,12	0,12	4	0,5	0,5
96125	M	84	SN	Sepsis	Blood	29.10.2021	C	*stG485.0*	0,015	0,03	0,03	0,5	0,12	0,12	4	64	1
96122	F	87	NW	no data	Blood	28.10.2021	G	*stC74a.0*	0,015	0,03	0,03	0,5	4	0,12	4	0,5	2
96115	M	62	SL	sept. Arthritis	Blood	23.10.2021	G	*stC74a.0*	0,015	0,03	0,03	0,5	0,12	0,12	4	16	1
96100	M	72	SH	Sepsis, Erysipel	Blood	27.10.2021	C	*stG62647.0*	0,015	0,03	0,03	0,5	0,12	0,12	2	0,5	1
96089	M	67	NW	no data	Blood	19.10.2021	G	*stG2078.0*	0,015	0,03	0,03	0,5	4	0,12	4	1	2
96083	M	67	NW	no data	Blood	19.10.2021	G	*stG2078.0*	0,015	0,03	0,03	0,5	4	0,12	4	1	2
95987	n/a	72	NW	no data	Blood	22.10.2021	C	*stG62647.0*	0,015	0,015	0,03	0,5	0,12	0,12	4	0,5	1
95986	M	83	NW	no data	Blood	19.10.2021	G	*stC74a.0*	0,015	0,03	0,03	0,5	0,12	0,12	4	4	1
95950	F	84	RP	Sepsis, Erysipel	Blood	16.10.2021	C	*stG6792.0*	0,015	0,03	0,03	0,5	0,12	0,12	4	0,5	0,5
95937	F	83	SN	no data	Blood	15.10.2021	C	*stG62647.0*	0,015	0,03	0,03	0,5	0,12	0,12	2	0,5	0,5
95917	M	0	SN	Bakteriämie	Blood	15.10.2021	C	*stG62647.0*	0,03	0,03	0,03	0,5	0,12	0,12	4	0,5	1
95893	F	51	RP	Sepsis, Fasciitis necroticans	Blood	07.10.2021	C	*stG62647.0*	0,015	0,03	0,03	0,5	0,12	0,12	4	0,5	1
95892	M	73	SL	Fasciitis necroticans,<br>Phlegmone	Blood	07.10.2021	G	*stG485.0*	0,015	0,03	0,03	0,5	0,12	0,12	4	1	1
95888	M	74	BY	Erysipel	Blood	09.10.2021	G	*stG2078.0*	0,015	0,03	0,03	0,5	0,12	0,12	4	1	1
95869	F	66	NW	no data	Blood	10.10.2021	C	*stG62647.0*	0,015	0,03	0,03	0,5	0,12	0,12	4	0,5	1
95824	M	78	NW	no data	Blood	04.10.2021	G	*stC5345.0*	0,015	0,03	0,03	0,5	0,12	0,12	2	0,5	1
95813	F	58	SH	Erysipel	Blood	03.10.2021	C	*stG62647.0*	0,015	0,03	0,03	0,5	0,12	0,12	4	0,5	1
95797	F	64	BE	Meningitis	CSF	23.09.2021	C	*stG62647.0*	0,015	0,03	0,03	0,5	0,12	0,12	4	0,5	0,5
95772	M	64	SN	Sepsis	Blood	24.09.2021	C	*stG2078.12*	0,015	0,03	0,03	0,5	0,12	0,12	4	0,5	0,5
95755	F	90	NW	Sepsis	Blood	29.09.2021	G	*stG485.0*	0,015	0,03	0,03	0,5	2	0,12	4	8	0,5
95748	M	85	NW	Pleuraempyem	Blood	24.09.2021	G	*stG2078.0*	0,015	0,03	0,03	0,5	0,12	0,12	2	0,5	1
95747	F	22	NW	Abszess	Gewebe	24.08.2021	G	*stG485.0*	0,015	0,015	0,015	0,5	0,12	0,12	4	0,5	1

95745	F	22	NW	Abszess	Gewebe	25.09.2021	G	*stC74a.0*	0,015	0,03	0,03	0,5	4	0,12	4	0,5	1
95740	F	76	NW	Sepsis, Pneumonie	Blood	26.09.2021	G	*stG652.0*	0,015	0,03	0,03	0,5	4	0,12	4	32	0,5
95667	F	82	SL	Erysipel	Blood	11.09.2021	G	*stC74a.0*	0,015	0,03	0,03	0,5	0,12	0,12	2	0,5	1
95664	M	83	NW	no data	Blood	08.09.2021	C	*stG62647.0*	0,015	0,03	0,03	0,5	0,12	0,12	4	0,5	1
95654	F	72	HE	no data	Blood	29.08.2021	G	*stG485.18*	0,015	0,015	0,015	0,5	0,12	0,12	2	0,5	1
95653	M	88	RP	sept. Arthritis	Blood	06.09.2021	G	*stC74a.0*	0,015	0,015	0,015	0,5	0,12	0,12	2	4	0,5
95641	M	93	NW	Sepsis	Blood	13.09.2021	C	*stG62647.0*	0,015	0,03	0,03	0,5	0,12	0,12	2	0,5	1
95636	F	66	HE	Erysipel	Blood	05.09.2021	G	*stC74a.0*	0,015	0,03	0,03	0,5	0,12	0,12	4	0,5	0,5
95630	F	66	BY	Knieschmerzen	Gelenkpunktat	08.09.2021	C	*stG62647.0*	0,015	0,03	0,03	0,5	0,12	0,12	2	0,5	1
95626	F	73	NW	no data	Blood	10.09.2021	G	*stGLP1.0*	0,015	0,03	0,03	0,5	0,12	0,12	4	64	1
95608	F	81	NW	no data	Blood	06.09.2021	C	*stG62647.0*	0,015	0,03	0,03	0,5	0,12	0,12	2	0,5	1
95606	M	82	HE	no data	Blood	03.09.2021	C	*stG62647.0*	0,015	0,03	0,03	0,5	0,12	0,12	4	0,5	1
95601	F	26	RP	Sepsis	Blood	02.09.2021	C	*stG62647.0*	0,015	0,03	0,03	0,5	0,12	0,12	2	0,5	1
95600	M	79	RP	Sepsis	Blood	02.09.2021	C	*stG62647.0*	0,015	0,015	0,015	0,5	0,12	0,12	2	0,25	1
95582	M	68	SH	Sepsis, sept. Arthritis	Blood	03.09.2021	G	*stC74a.0*	0,015	0,03	0,03	0,5	4	0,12	4	0,5	0,5
95571	F	82	SH	Erysipel	Blood	02.09.2021	G	*stG485.0*	0,015	0,03	0,03	0,5	0,12	0,12	4	4	1
95553	M	82	SH	no data	Blood	27.07.2021	C	*stG62647.0*	0,015	0,03	0,03	0,5	0,12	0,12	2	0,5	1
95552	M	57	BY	Ulzerationen an den Füßen	Swab wound	25.08.2021	C	*stG62647.0*	0,015	0,03	0,03	0,5	2	0,12	4	0,5	1
95542	F	84	SL	no data	Blood	21.08.2021	G	*stC74a.0*	0,015	0,03	0,03	0,5	8	0,12	4	0,5	1
95535	M	88	NW	no data	Blood	26.08.2021	C	*stG62647.11*	0,015	0,03	0,03	0,5	0,12	0,12	2	0,5	1
95531	F	90	SN	Arthritis	Gelenkpunktat	25.08.2021	C	*stG62647.0*	0,015	0,015	0,03	0,5	0,12	0,12	2	0,25	1
95530	F	78	SN	Bakteriämie	Blood	25.08.2021	C	*stG62647.0*	0,015	0,03	0,03	0,5	0,12	0,12	2	0,5	1
95502	M	85	NW	no data	Blood	18.08.2021	G	*stG2078.0*	0,015	0,03	0,03	0,5	0,12	0,12	4	1	2
95487	F	84	NW	no data	Blood	17.08.2021	G	*stG245.0*	0,015	0,03	0,03	0,5	256	256	4	8	0,5
95486	F	83	BY	Sepsis	Blood	18.08.2021	G	*stG245.0*	0,015	0,03	0,03	0,5	2	0,12	2	0,5	1
95463	M	93	BY	Erysipel Unterschenkel, Amputation Zeh	Swab wound	09.08.2021	C	*stG62647.0*	0,015	0,03	0,03	0,5	0,12	0,12	2	0,5	1
95438	F	77	NW	no data	Blood	10.08.2021	C	*stG62647.0*	0,03	0,06	0,03	0,5	0,12	0,12	2	0,5	1
95436	F	52	SH	Sepsis	Blood	12.08.2021	G	*stC74a.0*	0,015	0,03	0,015	0,5	256	256	2	8	0,5
95434	F	88	SH	Pneumonie	Blood	13.08.2021	C	*stG62647.0*	0,015	0,03	0,03	0,5	0,12	0,12	2	0,5	1
95433	M	63	BY	Erysipel	Blood	07.08.2021	C	*stG62647.0*	0,015	0,03	0,03	0,5	0,12	0,12	2	0,5	1
95432	M	38	NW	Sepsis	Blood	15.08.2021	C	*stG62647.0*	0,015	0,03	0,03	0,5	0,12	0,12	2	0,5	1
95429	M	41	SN	Bursitis	Gelenkpunktat	09.08.2021	C	*stG62647.0*	0,015	0,03	0,03	0,5	0,12	0,12	2	0,5	1
95407	M	68	NW	Sepsis	Blood	05.08.2021	C	*stG62647.0*	0,015	0,015	0,015	0,5	0,12	0,12	4	0,5	0,5
95406	M	72	NW	Sepsis	Blood	06.08.2021	G	*stG2078.0*	0,015	0,015	0,015	0,5	0,12	0,12	4	0,5	1
95399	M	59	BB	Knie-TEP-Infekt	Blood	03.08.2021	C	*stG62647.0*	0,015	0,03	0,03	0,5	0,12	0,12	4	0,5	1
95395	M	82	NW	Erysipel	Blood	04.08.2021	C	*stG62647.0*	0,015	0,015	0,03	0,5	0,12	0,12	4	0,5	1
95392	M	82	NW	no data	Blood	01.08.2021	G	*stC74a.0*	0,015	0,03	0,03	0,5	0,12	0,12	4	8	1
95381	M	39	BY	Thrombose Vena Iliaca, Spritzenabszess	Blood	02.08.2021	G	*stC74a.12*	0,015	0,03	0,03	0,5	4	0,12	4	0,5	1
95380	F	36	BY	Fasciitis necroticans Unterschenkel	Swab wound	30.07.2021	G	*stC74a.12*	0,015	0,03	0,03	0,5	4	0,12	4	0,5	1
95362	F	93	SL	Sepsis	Blood	28.07.2021	G	*stC74a.0*	0,015	0,03	0,03	0,5	0,12	0,12	4	4	0,5
95358	M	87	NW	no data	Blood	31.07.2021	C	*stG62647.0*	0,015	0,015	0,03	0,5	2	0,12	4	0,5	1
95351	M	77	NW	no data	Blood	30.07.2021	G	*stG485.0*	0,015	0,03	0,03	0,5	0,12	0,12	4	1	1
95349	M	72	SH	Schultersteife	Blood	29.07.2021	G	*stG485.0*	0,015	0,015	0,015	0,5	0,12	0,12	4	0,5	1
95348	F	84	SH	Erysipel	Blood	30.07.2021	C	*stG62647.0*	0,015	0,03	0,03	0,5	0,12	0,12	4	1	1
95345	M	66	NW	no data	Blood	28.07.2021	C	*stG62647.0*	0,015	0,03	0,03	0,5	0,12	0,12	4	0,5	1
95341	M	51	SL	Phlegmone	Blood	21.07.2021	C	*stG62647.0*	0,015	0,03	0,03	0,5	0,12	0,12	4	1	2
95338	M	72	SH	Erysipel	Blood	25.07.2021	C	*stG62647.0*	0,015	0,03	0,03	0,5	0,12	0,12	4	1	1
95337	F	81	NW	fieberhafter Infekt	Blood	27.07.2021	G	*stG245.0*	0,015	0,03	0,03	0,5	0,12	0,12	4	8	1
95326	M	46	NW	no data	Blood	23.07.2021	C	*stG62647.0*	0,015	0,03	0,03	0,5	0,12	0,12	4	0,5	1

95314	M	77	SH	Fokus unklar	Blood	20.07.2021	G	*stG480.12*	0,03	0,015	0,03	0,5	256	256	4	32	0,5
95288	F	84	HE	no data	Blood	13.07.2021	C	*stG62647.0*	0,015	0,03	0,03	0,5	0,12	0,12	4	0,5	1
95280	M	66	HE	no data	Blood	12.07.2021	G	*stG652.0*	0,015	0,03	0,03	0,5	0,12	0,12	4	64	1
95271	M	85	SH	no data	Blood	13.07.2021	G	*stC74a.0*	0,015	0,03	0,03	0,5	4	0,12	4	1	1
95251	M	83	HE	no data	Blood	04.07.2021	C	*stG62647.0*	0,015	0,03	0,03	0,5	0,12	0,12	4	0,5	1
95242	M	69	RP	no data	Blood	07.07.2021	C	*stG62647.0*	0,015	0,03	0,03	0,5	0,12	0,12	4	0,5	0,5
95237	F	93	NW	no data	Blood	13.07.2021	C	*stG62647.0*	0,015	0,03	0,03	0,5	0,12	0,12	4	0,5	1
95213	M	82	NW	no data	Blood	12.07.2021	C	*stG62647.0*	0,015	0,015	0,03	0,5	0,12	0,12	4	0,5	1
95212	M	71	NW	no data	Blood	12.07.2021	C	*stG62647.0*	0,015	0,03	0,03	0,5	0,12	0,12	4	0,5	1
95207	M	56	HE	no data	Blood	05.07.2021	G	*stC74a.0*	0,015	0,03	0,03	0,5	0,12	0,12	4	8	0,5
95199	F	69	SN	Kniegelenkinfektion	Gelenkpunktat	08.07.2021	G	*stC74a.0*	0,015	0,015	0,015	0,5	0,12	0,12	4	4	0,5
95188	M	74	SL	Phlegmone	Blood	03.07.2021	G	*stG485.0*	0,015	0,03	0,03	0,5	0,12	0,12	4	32	0,5
95184	M	89	SH	Sepsis	Blood	06.07.2021	C	*stG62647.0*	0,015	0,03	0,03	0,5	0,12	0,12	2	0,5	1
95161	F	95	SN	Sepsis	Blood	29.06.2021	C	*stG62647.0*	0,015	0,015	0,015	0,5	0,12	0,12	4	0,5	1
95158	M	83	SH	Pneumonie	Blood	29.06.2021	G	*stC74a.0*	0,015	0,03	0,03	0,5	4	0,12	4	0,5	0,5
95148	F	59	SH	Erysipel	Blood	25.06.2021	C	*stG62647.0*	0,015	0,03	0,03	0,5	0,12	0,12	4	0,5	1
95147	M	74	SH	Erysipel	Blood	26.06.2021	G	*stG485.0*	0,015	0,03	0,03	0,5	0,12	0,12	4	0,5	1
95141	M	79	NW	Fieber unklarer Genese	Blood	25.06.2021	G	*stC74a.0*	0,015	0,015	0,015	0,5	4	0,12	4	0,5	1
95133	M	63	NW	Sepsis	Blood	23.06.2021	G	*stG485.0*	0,015	0,015	0,03	0,5	0,12	0,12	4	2	1
95124	F	86	NW	Sepsis	Blood	25.06.2021	C	*stG62647.0*	0,015	0,03	0,03	0,5	0,12	0,12	2	0,5	1
95123	M	80	NW	no data	Blood	25.06.2021	G	*stG245.0*	0,015	0,03	0,03	0,5	256	256	4	16	0,5
95119	M	0	RP	Sepsis	Blood	22.06.2021	C	*stG62647.0*	0,015	0,015	0,03	0,5	0,12	0,12	2	0,5	0,5
95116	F	74	NW	Sepsis, Fieber	Blood	22.06.2021	A	*stG652.0*	0,015	0,03	0,03	0,5	0,12	0,12	2	16	1
95105	M	80	SN	Pneumonie	Blood	19.06.2021	C	*stG62647.0*	0,03	0,06	0,06	0,5	0,12	0,12	4	0,5	1
95099	M	70	NW	no data	Blood	18.06.2021	G	*stG6.3*	0,015	0,03	0,03	0,5	0,12	0,12	4	0,5	0,5
95079	F	91	SH	Bronchitis	Blood	15.06.2021	G	*stG485.0*	0,015	0,015	0,015	0,5	0,12	0,12	4	0,5	1
95069	F	24	RP	no data	Blood	09.06.2021	C	*stG62647.0*	0,015	0,03	0,03	0,5	0,12	0,12	4	0,5	1
95064	M	87	SH	Pneumonie	Blood	12.06.2021	C	*stG62647.0*	0,015	0,03	0,03	0,5	0,12	0,12	2	0,25	1
95054	F	51	NW	Sepsis	Blood	09.06.2021	C	*stG62647.0*	0,015	0,03	0,03	0,5	0,12	0,12	4	0,5	0,5
95050	M	73	NI	Sepsis	Blood	09.06.2021	A	*stG6.3*	0,015	0,03	0,03	0,5	0,12	0,12	4	8	0,5
95043	M	58	RP	Erysipel	Blood	09.06.2021	G	*stG2078.0*	0,015	0,03	0,03	0,5	0,12	0,12	2	0,25	1
95034	F	86	NW	no data	Blood	10.06.2021	C	*stG62647.0*	0,015	0,03	0,03	0,5	0,12	0,12	4	0,5	1
95028	M	49	SH	Sepsis, Endokarditis	Blood	07.06.2021	G	*stG485.0*	0,015	0,03	0,03	0,5	0,12	0,12	4	32	1
95021	M	57	SL	Erysipel	Blood	02.06.2021	C	*stG62647.0*	0,015	0,015	0,015	0,5	0,12	0,12	4	0,5	0,5
95003	F	83	NW	Sepsis	Blood	03.06.2021	C	*stG62647.0*	0,015	0,015	0,03	0,5	0,12	0,12	4	0,5	1
94980	M	97	SH	Sepsis	Blood	28.05.2021	G	*stG480.0*	0,015	0,03	0,03	0,5	2	0,12	4	0,5	2
94964	F	63	SN	sept. Arthritis	Gelenkpunktat	26.05.2021	C	*stG62647.8*	0,015	0,03	0,03	0,5	0,12	0,12	4	2	1
94963	M	35	SN	Sepsis	Blood	24.05.2021	G	*stG485.17*	0,015	0,03	0,03	0,5	0,12	0,12	4	8	1
94941	F	31	SH	Sepsis	Blood	21.05.2021	C	*stG62647.0*	0,015	0,03	0,03	0,5	1	0,12	4	1	1
94940	M	81	SH	Wundinfekt Leiste	Blood	25.05.2021	G	*stC74a.16*	0,03	0,12	0,06	0,5	4	0,12	4	1	1
94930	M	70	SH	no data	Blood	17.05.2021	G	*stG245.0*	0,015	0,03	0,03	0,5	0,12	0,12	4	0,5	1
94928	M	87	SH	Sepsis, Pneumonie	Blood	19.05.2021	G	*stC74a.0*	0,015	0,03	0,03	0,5	0,12	0,12	4	0,5	1
94906	F	79	NW	no data	Blood	15.05.2021	C	*stG62647.0*	0,015	0,03	0,03	0,5	0,12	0,12	4	0,5	0,5
94905	M	81	NW	no data	Blood	14.05.2021	G	*stG6.3*	0,015	0,015	0,015	0,5	8	0,12	4	1	1
94903	M	83	NW	Sepsis	Blood	19.05.2021	G	*stC74a.0*	0,015	0,015	0,015	0,5	0,12	0,12	4	8	0,5
94883	M	67	NW	no data	Blood	12.05.2021	G	*stG480.0*	0,015	0,03	0,03	0,5	64	256	8	0,5	0,5
94867	F	89	NW	Sepsis	Blood	07.05.2021	G	*stG2078.0*	0,015	0,03	0,03	0,5	0,12	0,12	2	0,5	1
94814	M	86	NW	no data	Blood	29.04.2021	G	*stC74a.0*	0,015	0,03	0,03	0,5	0,12	0,12	4	8	1
94796	M	65	NW	no data	Blood	28.04.2021	C	*stG62647.0*	0,015	0,03	0,03	0,5	0,12	0,12	4	0,5	1
94765	F	83	SH	Pneumonie, Wundinfektion	Blood	22.04.2021	C	*stG62647.0*	0,015	0,03	0,03	0,5	0,12	0,12	2	0,5	1
94753	M	0	RP	Sepsis	Blood	14.04.2021	C	*stG62647.0*	0,015	0,03	0,03	0,5	0,12	0,12	2	0,5	0,5
94711	M	89	SH	Erysipel	Blood	11.04.2021	C	*stG62647.0*	0,015	0,03	0,03	0,5	0,12	0,12	4	0,5	1

94701	F	90	SN	Bakteriämie	Blood	09.04.2021	C	*stG62647.0*	0,015	0,03	0,03	0,5	0,12	0,12	2	0,25	0,5
94700	M	83	SH	Pneumonie	Blood	10.04.2021	C	*stG62647.0*	0,015	0,03	0,03	0,5	0,12	0,12	4	0,5	1
94683	M	59	NW	no data	Blood	07.04.2021	C	*stG62647.0*	0,015	0,03	0,03	0,5	0,12	0,12	4	0,5	1
94666	M	64	RP	Fasciitis necroticans	Blood	30.03.2021	G	*stG485.0*	0,015	0,03	0,03	0,5	0,12	0,12	2	0,25	1
94658	M	81	SH	Erysipel	Blood	30.03.2021	G	*stG166b.0*	0,015	0,03	0,03	0,5	0,12	0,12	4	0,5	0,5
94655	F	85	NW	no data	Blood	01.04.2021	C	*stG62647.0*	0,015	0,015	0,015	0,5	0,12	0,12	2	0,25	2
94640	F	97	SN	Bakteriämie	Blood	27.03.2021	C	*stG62647.0*	0,015	0,015	0,015	0,5	0,12	0,12	2	0,25	1
94633	F	74	RP	no data	Blood	26.03.2021	C	*stG62647.0*	0,015	0,015	0,015	0,5	0,12	0,12	4	0,25	1
94616	M	60	SH	Erysipel	Blood	26.03.2021	G	*stG485.0*	0,03	0,03	0,03	0,5	0,12	0,12	4	8	1
94615	M	62	BY	Weichteilinfektion Fuß, Sepsis, Gangrän	Blood	24.03.2021	G	*stG6.0*	0,015	0,015	0,015	0,5	0,12	0,12	2	32	1
94562	M	66	SH	Wundinfekt nach Neck-Dissection	Blood	15.03.2021	G	*stG2078.0*	0,015	0,03	0,03	0,5	0,12	0,12	4	0,5	1
94560	F	77	BY	Wundgewebeentfernung Unterschenkel	Swab wound	12.03.2021	C	*stG62647.0*	0,015	0,03	0,03	0,5	0,12	0,12	4	0,5	1
94559	F	79	BY	Infektion Unterschenkel Ulzerationen, Sepsis	Swab wound	11.03.2021	C	*stG62647.0*	0,015	0,03	0,03	0,5	0,12	0,12	4	0,5	1
94526	F	17	NW	Erysipel	Blood	14.03.2021	G	*stC5345.0*	0,015	0,015	0,015	0,5	0,12	0,12	4	0,5	1
94509	F	83	BY	no data	Blood	13.03.2021	G	*stG245.0*	0,015	0,03	0,03	0,5	2	0,12	4	0,5	0,5
94481	F	88	NW	Sepsis	Blood	26.02.2021	C	*stG62647.0*	0,015	0,03	0,03	0,5	0,12	0,12	4	0,5	1
94480	F	92	NW	Sepsis	Blood	25.02.2021	G	*stG480.0*	0,015	0,03	0,03	0,5	0,12	0,12	4	0,5	0,5
94474	F	77	NW	Wundinfektion, Erysipel, Phlegmone	Swab Ferse	10.02.2021	G	*stG10.0*	0,015	0,015	0,03	0,5	0,12	0,12	4	0,5	1
94466	F	89	BY	Erysipel Unterschenkel, Urosepsis	Urin	27.02.2021	C	*stG62647.0*	0,015	0,015	0,015	0,5	0,12	0,12	4	0,5	1
94451	F	76	NW	Sepsis	Blood	26.02.2021	C	*stG62647.0*	0,015	0,03	0,03	0,5	0,12	0,12	4	0,5	0,5
94450	M	52	NW	Sepsis	Blood	24.02.2021	C	*stC74a.0*	0,015	0,03	0,03	0,5	4	0,12	4	0,5	1
94449	M	87	NW	Sepsis	Blood	24.02.2021	C	*stG62647.0*	0,015	0,03	0,03	0,5	0,12	0,12	4	0,5	1
94446	M	73	BY	Bursitis präpatellaris	Biopsie	21.02.2021	G	*stG480.0*	0,015	0,015	0,015	0,5	0,12	0,12	4	0,5	1
94445	F	70	BY	Wundinfektion	Swab wound	22.02.2021	C	*stG62647.0*	0,015	0,03	0,03	0,5	0,12	0,12	4	0,5	1
94439	M	81	NW	Fieber	Blood	24.02.2021	G	*stC839.2*	0,015	0,03	0,03	0,5	0,12	0,12	4	32	1
94436	M	72	NW	Sepsis	Blood	19.02.2021	G	*stC5345.0*	0,015	0,015	0,015	0,5	2	0,12	4	0,5	1
94433	M	62	NW	Sepsis	Blood	11.02.2021	G	*stC74a.0*	0,015	0,03	0,03	0,5	0,12	0,12	4	32	0,5
94430	M	67	SH	Erysipel	Blood	23.02.2021	G	*stG643.0*	0,015	0,015	0,015	0,5	2	0,12	4	16	1
94420	F	28	BY	Brustinfektion, Mastitis	Swab wound	17.02.2021	G	*stC74a.0*	0,015	0,03	0,03	0,5	0,12	0,12	4	0,5	1
94418	M	78	NW	Sepsis	Blood	18.02.2021	C	*stG62647.0*	0,015	0,015	0,015	0,5	0,12	0,12	4	0,5	1
94403	F	87	HE	no data	Blood	13.02.2021	G	*stG6.1*	0,015	0,03	0,03	0,5	16	0,12	4	0,5	0,5
94387	M	61	BY	Ulzerationen an den Füßen, Arteriovenous occlusive disease	Swab wound	10.02.2021	G	*stG480.0*	0,015	0,015	0,015	0,5	0,12	0,12	4	32	0,5
94380	M	62	NW	no data	Blood	14.02.2021	C	*stG62647.0*	0,015	0,015	0,015	0,5	0,12	0,12	4	16	0,5
94377	F	90	SH	Erysipel	Blood	09.02.2021	G	*stG10.0*	0,015	0,06	0,06	0,5	0,12	0,12	4	32	1
94376	M	86	SH	Pneumonie, Erysipel, Apoplex	Blood	08.02.2021	G	*stG485.0*	0,015	0,03	0,03	0,5	0,12	0,12	4	4	1
94371	F	74	HE	no data	Blood	08.02.2021	G	*stG10.0*	0,015	0,015	0,015	0,5	0,12	0,12	4	64	1
94352	M	86	SH	Urosepsis	Blood	05.02.2021	C	*stG62647.0*	0,015	0,03	0,03	0,5	0,12	0,12	4	0,5	1
94344	F	74	NW	Sepsis, Erysipel, Phlegmone	Blood	02.02.2021	G	*stG62647.0*	0,015	0,03	0,03	0,5	0,12	0,12	4	0,5	1
94329	M	83	NW	Sepsis	Blood	01.02.2021	G	*stG480.0*	0,015	0,015	0,015	0,5	0,12	0,12	4	0,5	1
94328	M	61	NW	Sepsis	Blood	31.01.2021	C	*stG2574.3*	0,015	0,015	0,015	0,5	0,12	0,12	4	0,5	1
94308	F	81	NW	Endokarditis	Blood	23.01.2021	G	*stG485.0*	0,015	0,015	0,015	0,5	0,12	0,12	4	0,5	1
94279	M	90	NW	Pneumonie, Pankreasraumforderung	Blood	17.01.2021	G	*stC74a.0*	0,015	0,03	0,03	0,5	4	0,12	4	0,5	1
94274	F	70	NW	Sepsis	Blood	16.01.2021	C	*stG62647.0*	0,015	0,03	0,03	0,5	0,12	0,12	4	0,5	1
94265	M	57	NW	Sepsis	Blood	17.01.2021	G	*stC839.2*	0,015	0,03	0,03	0,5	0,12	0,12	4	64	1

94264	F	52	NW	Sepsis, Pankreatitis	Blood	16.01.2021	C	*stG62647.0*	0,015	0,015	0,03	0,5	0,12	0,12	2	0,5	1
94250	F	36	NW	Sepsis	Blood	15.01.2021	G	*stG480.0*	0,015	0,015	0,015	0,5	0,12	0,12	4	0,5	1
94249	M	80	RP	Phlegmone	Blood	11.01.2021	C	*stG62647.0*	0,015	0,03	0,03	0,5	0,12	0,12	4	0,5	1
94189	F	70	NW	Sepsis, Fieber, HWI	Blood	11.01.2021	G	*stC5345.0*	0,015	0,03	0,03	0,5	4	8	4	0,5	0,5
94186	M	81	RP	sept. Arthritis	Blood	04.01.2021	G	*stG485.0*	0,015	0,03	0,03	0,5	0,12	0,12	4	0,5	1
94185	F	86	SL	Erysipel	Blood	09.01.2021	G	*stG2078.0*	0,015	0,015	0,015	0,5	0,12	0,12	4	0,5	1
94176	F	94	NW	Phlegmone	Blood	07.01.2021	C	*stG62647.0*	0,015	0,03	0,03	0,5	0,12	0,12	4	0,5	0,5
94172	M	54	SH	Sepsis, Pneumonie	Blood	06.01.2021	G	*stG643.0*	0,015	0,03	0,03	0,5	0,12	0,12	4	2	1
94159	F	52	BY	Weichgewebeinfektion	Blood	01.01.2021	C	*stG62647.0*	0,015	0,03	0,03	0,5	0,12	0,12	4	0,5	1
94157	M	52	NW	Sepsis	Blood	09.01.2021	C	*stG62647.0*	0,015	0,03	0,03	0,5	4	0,12	4	0,5	0,5
94151	F	78	RP	Phlegmone	Blood	01.01.2021	C	*stG62647.0*	0,015	0,03	0,03	0,5	0,12	0,12	4	0,5	1
94148	M	65	NW	no data	Blood	07.01.2021	C	*stG62647.0*	0,015	0,03	0,03	0,5	0,12	0,12	4	0,5	1
94138	F	72	BW	no data	Blood	02.01.2021	C	*stG62647.0*	0,015	0,03	0,03	0,5	0,12	0,12	4	0,5	1
94137	F	92	BW	no data	Blood	02.01.2021	C	*stG62647.0*	0,015	0,03	0,03	0,5	0,12	0,12	4	0,5	1
94119	M	70	NW	no data	Blood	02.01.2021	G	*stC74a.0*	0,015	0,03	0,03	0,5	8	0,12	4	1	1
94118	n/a	71	NW	no data	Blood	29.12.2020	C	*stG62647.0*	0,015	0,03	0,03	0,5	0,12	0,12	4	0,5	1
94109	M	45	SH	no data	Blood	29.12.2020	C	*stG653.2*	0,015	0,015	0,015	0,5	0,12	0,12	2	0,5	0,5
94102	M	78	SN	Bakteriämie	Blood	27.12.2020	G	*stG62647.0*	0,015	0,03	0,03	0,5	0,12	0,12	4	0,5	1
94100	F	93	SN	Bakteriämie	Blood	28.12.2020	C	*stG62647.0*	0,015	0,03	0,03	0,5	0,12	0,12	4	0,5	1
94088	M	85	NW	Sepsis, Fieber	Blood	21.12.2020	C	*stG62647.0*	0,015	0,03	0,03	0,5	0,12	0,12	4	0,5	1
94066	F	86	BY	Erysipel Unterschenkel, Sepsis	Blood	19.12.2020	G	*stC839.2*	0,015	0,015	0,015	0,5	0,12	0,12	4	64	1
94065	M	81	BW	Sepsis	Blood	22.12.2020	G	*stG840.0*	0,015	0,03	0,03	0,5	0,12	0,12	2	0,5	1
94056	M	72	RP	sept. Arthritis	Blood	19.12.2020	C	*stG62647.0*	0,015	0,03	0,03	0,5	0,12	0,12	2	0,5	1
94052	M	90	NW	no data	Blood	21.12.2020	C	*stG62647.0*	0,015	0,015	0,03	0,5	0,12	0,12	2	0,5	1
94035	M	60	NW	Sepsis	Blood	14.12.2020	G	*stG643.0*	0,03	0,06	0,06	0,5	256	0,12	4	32	1
94032	M	87	SH	Mazeration	Blood	14.12.2020	G	*stG10.0*	0,015	0,03	0,03	0,5	0,12	0,12	2	32	1
94009	M	79	NW	no data	Blood	08.12.2020	G	*stC74a.0*	0,015	0,03	0,03	0,5	0,12	0,12	2	64	0,5
93986	F	74	NW	Sepsis, sept. Arthritis	Blood	08.12.2020	G	*stC74a.0*	0,015	0,03	0,03	0,5	2	0,12	4	0,5	1
93978	M	77	BY	Bakteriämie	Blood	07.12.2020	C	*stG485.0*	0,015	0,015	0,015	0,5	0,12	0,12	4	32	0,5
93977	M	63	RP	Pneumonie	Blood	06.12.2020	G	*stG6.1*	0,015	0,015	0,015	0,5	0,12	0,12	4	0,5	0,5
93975	M	68	RP	Sepsis	Blood	06.12.2020	C	*stG62647.0*	0,015	0,03	0,03	0,5	0,12	0,12	4	0,5	1
93970	M	64	HE	no data	Blood	05.12.2020	G	*stG10.0*	0,015	0,03	0,03	0,5	0,12	0,12	4	32	1
93965	M	79	BY	Sepsis, Phlegmone, Abszess	Blood	07.12.2020	G	*stG643.0*	0,015	0,03	0,03	0,5	0,12	0,12	4	64	0,5
93961	n/a	74	HH	Protheseninfektion	Gewebe Knie	06.12.2020	G	*stC74a.0*	0,015	0,03	0,03	0,5	0,12	0,12	4	32	0,5
93697	M	83	SH	Sepsis	Blood	02.12.2020	G	*stG2078.0*	0,015	0,03	0,03	0,5	0,12	0,12	4	0,5	1
93581	F	85	RP	no data	Blood	29.11.2020	G	*stC74a.0*	0,015	0,015	0,03	0,5	0,12	0,12	4	4	1
93580	M	66	RP	Erysipel	Blood	29.11.2020	G	*stG6.3*	0,015	0,03	0,03	0,5	0,12	0,12	4	0,5	1
93550	F	56	BY	Nekrose am Zeh	Biopsie	23.11.2020	C	*stG62647.0*	0,015	0,03	0,03	0,5	0,12	0,12	2	0,5	1
93541	M	85	SN	Sepsis	Blood	29.11.2020	G	*stG653.0*	0,015	0,03	0,03	0,5	0,12	0,12	4	1	0,5
93527	F	77	NW	Erysipel	Blood	27.11.2020	C	*stG62647.4*	0,015	0,03	0,03	0,5	0,12	0,12	2	0,25	1
93521	M	82	NW	no data	Blood	27.11.2020	C	*stG62647.0*	0,015	0,03	0,03	0,5	0,12	0,12	4	1	1
93495	F	44	BY	Abszess, Krampfader nach Fußgelenkoperation	Swab	20.11.2020	C	*stG62647.0*	0,015	0,03	0,06	0,5	0,12	0,12	4	0,5	1
93490	M	60	NW	Sepsis	Blood	20.11.2020	G	*stG643.0*	0,015	0,03	0,03	0,5	0,12	0,12	4	64	1
93474	F	88	RP	Sepsis	Blood	16.11.2020	C	*stG62647.0*	0,015	0,03	0,03	0,5	0,12	0,12	2	0,5	1
93471	F	58	SN	Protheseninfektion	Gelenkpunktat	19.11.2020	G	*stG485.0*	0,015	0,03	0,03	0,5	0,12	0,12	4	8	1
93463	M	90	NW	Sepsis	Blood	15.11.2020	C	*stG62647.0*	0,015	0,03	0,03	0,5	0,12	0,12	4	0,5	1
93432	M	57	SN	no data	Blood	10.11.2020	C	*stG62647.0*	0,015	0,03	0,03	0,5	0,12	0,12	4	0,5	1
93419	M	89	SH	no data	Blood	02.11.2020	C	*stG62647.0*	0,015	0,03	0,03	0,5	0,12	0,12	4	0,5	1
93414	M	79	RP	Endokarditis	Blood	03.11.2020	G	*stG245.0*	0,015	0,015	0,015	0,5	4	0,12	4	0,5	0,5
93408	M	66	SN	Sepsis, Erysipel	Blood	09.11.2020	G	*stG6.1*	0,015	0,03	0,03	0,5	0,12	0,12	2	0,5	0,5
93399	M	59	RP	no data	Blood	07.11.2020	G	*stG10.0*	0,015	0,03	0,03	0,5	0,12	0,12	4	4	1

93383	M	79	BY	Empyem	Puncture Gelenk	04.11.2020	G	*stG652.1*	0,015	0,03	0,03	0,5	0,12	0,12	4	32	0,5
93382	M	55	NW	Sepsis	Blood	02.11.2020	G	*stG485.0*	0,015	0,015	0,015	0,5	0,12	0,12	4	32	1
93379	F	45	SN	eitrige Arthritis	Puncture Gelenk	04.11.2020	C	*stG62647.0*	0,015	0,03	0,03	0,5	0,12	0,12	2	0,5	1
93364	M	69	BY	Sepsis	Blood	29.10.2020	C	*stG62647.0*	0,015	0,03	0,03	0,5	0,12	0,12	4	0,5	1
93363	F	59	BY	Abszess linke Hüfte	Biopsie Unterschenkel	29.10.2020	C	*stG62647.0*	0,015	0,03	0,03	0,5	0,12	0,12	4	0,5	1
93331	M	69	NW	Erysipel	Blood	26.10.2020	G	*stG480.0*	0,015	0,015	0,015	0,5	0,12	0,12	4	0,5	1
93324	M	92	SN	Sepsis, Wundinfektion	Blood	29.10.2020	C	*stG62647.0*	0,015	0,03	0,03	0,5	0,12	0,12	4	0,5	1
93282	M	81	BY	Osteomyelitis rechter Zeigefinger	Swab itraoperativ	14.10.2020	C	*stG62647.0*	0,015	0,03	0,03	0,5	0,12	0,12	4	0,5	1
93266	M	76	NW	no data	Blood	19.10.2020	G	*stG5420.0*	0,015	0,015	0,015	0,5	0,12	0,12	4	0,5	0,5
93265	M	56	NW	no data	Blood	19.10.2020	C	*stG62647.0*	0,015	0,015	0,015	0,5	0,12	0,12	4	0,5	1
93259	F	70	RP	no data	Blood	13.10.2020	G	*stG485.14*	0,015	0,015	0,015	0,5	8	2	4	0,5	1
93244	F	36	BY	Sepsis	Urin	09.10.2020	G	*stG6.1*	0,015	0,03	0,03	0,5	0,12	0,12	2	0,25	0,5
93229	M	75	HE	Infekt, Fieber	Blood	12.10.2020	C	*stG62647.0*	0,015	0,015	0,03	0,5	0,12	0,12	2	0,5	1
93228	M	70	HE	no data	Blood	13.10.2020	C	*stG62647.0*	0,015	0,015	0,015	0,5	0,12	0,12	4	0,5	1
93203	F	65	HE	no data	Blood	08.10.2020	G	*stC74a.0*	0,015	0,03	0,03	0,5	0,12	0,12	4	0,5	1
93197	F	70	NW	Sepsis	Blood	10.10.2020	G	*stG2078.0*	0,015	0,015	0,015	0,5	0,12	0,12	4	0,5	1
93171	M	55	NW	no data	Blood	09.10.2020	C	*stG62647.0*	0,015	0,03	0,03	0,5	0,12	0,12	2	0,5	1
93162	M	84	SH	Weichteilinfekt Fuß	Blood	04.10.2020	C	*stG62647.0*	0,015	0,03	0,03	0,5	0,12	0,12	4	0,5	1
93159	M	70	SH	Pneumonie	Blood	03.10.2020	C	*stG62647.0*	0,015	0,015	0,015	0,5	0,12	0,12	4	0,5	1
93140	M	50	BY	Sepsis, Pneumonie	Blood	30.09.2020	C	*stG2574.3*	0,015	0,03	0,03	0,5	0,12	0,12	4	0,5	1
93117	M	91	SN	Verletzung der Hüfte	Blood	01.10.2020	C	*stG62647.0*	0,015	0,03	0,03	0,5	0,12	0,12	4	0,5	0,5
93054	F	64	NW	Sepsis	Blood	21.09.2020	A	*stG652.0*	0,015	0,03	0,03	0,5	0,12	0,12	4	16	1
93039	M	77	SH	Sepsis	Blood	16.09.2020	G	*stC74a.0*	0,015	0,03	0,03	0,5	0,12	0,12	4	0,5	1
93038	M	53	SN	Sepsis, Wundinfekt	Blood	14.09.2020	G	*stG485.0*	0,015	0,03	0,03	0,5	0,12	0,12	4	0,5	1
93023	M	58	NW	no data	Blood	11.09.2020	C	*stG62647.0*	0,015	0,015	0,015	0,5	0,12	0,12	4	0,5	1
93013	M	78	NW	Erysipel, Fieber	Blood	14.09.2020	G	*stC839.7*	0,015	0,015	0,03	0,5	0,12	0,12	4	32	1
93009	M	62	SN	Sepsis, STSS	Blood	14.09.2020	G	*stG6792.0*	0,015	0,015	0,03	0,5	0,12	0,12	4	0,5	0,5
92992	M	72	NW	Sepsis	Blood	12.09.2020	A	*stG485.0*	0,015	0,03	0,03	0,5	0,12	0,12	4	2	1
92978	M	82	HE	no data	Blood	04.09.2020	G	*stG480.0*	0,015	0,015	0,015	0,5	0,12	0,12	4	0,5	1
92951	M	72	HE	no data	Blood	31.08.2020	C	*stG62647.0*	0,015	0,015	0,03	0,5	0,12	0,12	4	0,5	0,5
92945	M	58	SH	Pneumonie	Blood	02.09.2020	C	*stG62647.0*	0,015	0,03	0,03	0,5	0,12	0,12	4	0,25	1
92930	F	59	RP	Sepsis, Erysipel	Blood	27.08.2020	C	*stG62647.0*	0,015	0,015	0,015	0,5	0,12	0,12	2	0,25	1
92929	M	41	SL	Sepsis, Erysipel	Blood	26.08.2020	G	*stC74a.0*	0,015	0,03	0,03	0,5	4	0,12	4	0,5	1
92925	F	86	SL	Erysipel	Blood	25.08.2020	C	*stG62647.0*	0,015	0,03	0,03	0,5	0,12	0,12	4	0,5	1
92906	M	83	TH	Fieber	Blood	27.08.2020	C	*stG62647.0*	0,015	0,03	0,03	0,5	0,12	0,12	4	0,5	1
92905	F	71	SN	Infekt	Puncture ascites	27.08.2020	G	*stG245.0*	0,015	0,03	0,03	0,5	4	0,12	4	32	0,5
92896	M	72	NW	Sepsis	Blood	19.08.2020	G	*stG30.0*	0,03	0,03	0,03	0,5	0,12	0,12	4	4	1
92895	M	79	NW	Sepsis	Blood	21.08.2020	G	*stC5345.0*	0,015	0,03	0,03	0,5	0,12	0,12	4	0,5	1
92894	M	90	NW	no data	Blood	16.08.2020	A	*stG485.0*	0,015	0,03	0,03	0,5	0,12	0,12	4	16	1
92890	M	64	NW	Sepsis, Pneumonie	Blood	24.08.2020	C	*stG62647.0*	0,015	0,03	0,03	0,5	0,12	0,12	4	0,5	1
92882	M	59	SH	Pneumonie, Erysipel	Blood	16.08.2020	G	*stC5345.0*	0,015	0,03	0,03	0,5	0,12	0,12	4	0,5	0,5
92876	M	82	SL	no data	Blood	08.08.2020	A	*stG652.0*	0,015	0,03	0,03	0,5	0,12	0,12	4	8	1
92869	M	70	BY	Wundinfektion	Swab wound	11.08.2020	G	*stC74a.0*	0,015	0,03	0,03	0,5	4	0,12	4	0,5	1
92868	M	65	BY	Wundinfektion	Swab wound	13.08.2020	G	*stG485.0*	0,015	0,015	0,015	0,5	0,12	0,12	4	0,5	1
92866	F	86	SL	Erysipel	Blood	13.08.2020	C	*stC9431.0*	0,015	0,03	0,03	0,5	0,12	0,12	4	2	0,5
92855	M	76	NI	Sepsis, Erysipel	Blood	11.08.2020	C	*stG62647.0*	0,015	0,015	0,03	0,5	0,12	0,12	4	0,5	1
92801	M	89	RP	Erysipel	Blood	02.08.2020	G	*stG2078.0*	0,015	0,03	0,06	0,5	0,12	0,12	4	0,5	0,5
92797	F	62	SN	Fieber	Blood	05.08.2020	G	*stG245.0*	0,015	0,03	0,03	0,5	0,12	0,12	4	1	0,5
92796	M	89	SH	Sepsis, Pneumonie	Blood	03.08.2020	G	*stG652.1*	0,015	0,03	0,03	0,5	0,12	0,12	4	32	0,5
92769	M	67	SH	Erysipel	Blood	30.07.2020	C	*stG62647.0*	0,015	0,03	0,03	0,5	0,12	0,12	4	0,5	0,5

92742	M	81	NW	no data	Blood	27.07.2020	G	*stC5345.0*	0,015	0,03	0,03	0,5	0,12	0,12	4	0,5	1
92727	M	64	NW	Erysipel, Infiltrat Lunge	Blood	23.07.2020	C	*stG62647.0*	0,015	0,015	0,015	0,5	0,12	0,12	4	0,5	1
92726	F	66	BY	Sepsis, Aszites	Puncture ascites	22.07.2020	G	*stG485.0*	0,015	0,03	0,03	0,5	0,12	0,12	4	0,5	1
92707	M	63	NW	Sepsis	Blood	21.07.2020	G	*stG480.0*	0,015	0,015	0,015	0,5	0,12	0,12	4	0,5	1
92702	F	88	BY	Erysipel Unterschenkel, Sepsis	Blood	19.07.2020	C	*stG62647.0*	0,015	0,03	0,03	0,5	0,12	0,12	2	0,5	1
92695	F	82	BY	Harnwegsinfekt, Sepsis	Urin	14.07.2020	C	*stG62647.0*	0,015	0,03	0,03	0,5	0,12	0,12	2	0,5	1
92684	M	72	BY	no data	Blood	16.07.2020	C	*stG62647.0*	0,015	0,015	0,015	0,5	0,12	0,12	4	0,5	1
92683	F	80	BY	Sepsis	Blood	18.07.2020	G	*stG652.0*	0,015	0,03	0,03	0,5	0,12	0,12	4	32	1
92674	F	81	SN	Erysipel	Blood	11.07.2020	G	*stC74a.0*	0,015	0,015	0,03	0,5	2	0,12	4	0,25	1
92673	F	33	SN	Protheseninfektion	Prothesenmaterial	10.07.2020	G	*stG485.0*	0,015	0,015	0,03	0,5	0,12	0,12	4	0,25	1
92648	F	82	RP	no data	Blood	05.07.2020	C	*stG62647.0*	0,015	0,03	0,03	0,5	0,12	0,12	2	0,5	1
92647	M	87	RP	no data	Blood	03.07.2020	C	*stG62647.0*	0,015	0,015	0,03	0,5	0,12	0,12	2	0,5	1
92645	F	57	RP	Erysipel	Blood	02.07.2020	C	*stG62647.0*	0,015	0,03	0,03	0,5	0,12	0,12	4	0,5	1
92640	M	85	SN	Sepsis	Blood	04.07.2020	G	*stC5345.0*	0,015	0,03	0,03	0,5	0,12	0,12	4	0,5	1
92639	M	87	SN	Sepsis	Blood	03.07.2020	G	*stG485.0*	0,015	0,03	0,015	0,5	0,12	0,12	4	0,5	1
92624	M	68	SH	no data	Blood	01.07.2020	G	*stG652.7*	0,015	0,06	0,06	0,5	0,12	0,12	4	16	4
92601	M	45	RP	Meningitis	Blood	27.06.2020	C	*stG62647.0*	0,015	0,03	0,03	0,5	0,12	0,12	4	0,5	0,5
92600	F	87	SN	Arthritis	Gelenkpunktat	29.06.2020	G	*stGLP1.2*	0,015	0,015	0,015	0,5	256	128	4	64	0,5
92579	F	59	SN	no data	Blood	23.06.2020	C	*stG62647.0*	0,015	0,03	0,03	0,5	0,12	0,12	2	0,25	1
92578	M	44	SN	no data	Biopsie Kniegelenk	21.06.2020	G	*stG480.0*	0,015	0,03	0,03	0,5	0,12	0,12	4	0,5	0,5
92577	F	82	SN	no data	Blood	19.06.2020	C	*stG62647.0*	0,015	0,03	0,03	0,5	0,12	0,12	2	0,5	1
92576	M	26	SN	Erguss Hüftgelenk	Gelenkpunktat Hüfte	17.06.2020	C	*stG62647.0*	0,015	0,03	0,03	0,5	0,12	0,12	4	0,25	1
92575	M	44	SN	Bursitis praepatellaris	Swab intraoperativ	21.06.2020	G	*stG480.0*	0,015	0,03	0,03	0,5	0,12	0,12	4	0,5	0,5
92572	M	65	SH	Erysipel	Blood	22.06.2020	G	*stG245.0*	0,015	0,03	0,03	0,5	256	256	4	8	0,5
92566	M	60	SL	Sepsis, Pneumonie	Blood	17.06.2020	G	*stG643.0*	0,015	0,015	0,015	0,5	0,12	0,12	4	1	0,5
92555	M	73	RP	Erysipel	Blood	15.06.2020	C	*stG62647.0*	0,015	0,015	0,03	0,5	1	0,12	4	0,5	1
92538	M	77	NW	Sepsis	Blood	16.06.2020	C	*stG62647.0*	0,015	0,03	0,03	0,5	0,12	0,12	4	0,5	1
92536	F	84	NW	no data	Blood	15.06.2020	C	*stG62647.3*	0,015	0,03	0,03	0,5	16	256	4	0,5	1
92494	M	81	SH	Sepsis, unklarer Infekt	Blood	03.06.2020	G	*stC74a.0*	0,015	0,03	0,03	0,5	4	0,12	4	0,5	1
92488	F	81	NW	Sepsis	Blood	04.06.2020	G	*stG840.0*	0,015	0,03	0,03	0,5	0,12	0,12	4	8	1
92484	M	76	SN	Erysipel, V.a. Endokarditis	Blood	04.06.2020	C	*stG62647.0*	0,015	0,015	0,015	0,5	0,12	0,12	2	0,25	1
92448	M	43	RP	no data	Blood	02.06.2020	G	*stG485.0*	0,015	0,03	0,03	0,5	0,12	0,12	4	4	0,5
92406	M	84	SL	no data	Blood	17.05.2020	C	*stG62647.0*	0,015	0,015	0,015	0,5	0,12	0,12	4	0,25	1
92400	n/a	95	HE	fieberhafter Infekt	Blood	20.05.2020	G	*stG245.0*	0,015	0,03	0,03	0,5	0,12	0,12	4	0,5	0,5
92394	M	72	HE	no data	Blood	16.05.2020	G	*stG652.17*	0,015	0,015	0,03	0,5	0,12	0,12	4	0,5	0,5
92391	M	86	SH	no data	Blood	18.05.2020	C	*stG62647.0*	0,015	0,03	0,03	0,5	0,12	0,12	4	0,5	1
92362	F	45	BY	no data	Blood	13.05.2020	G	*stG6.3*	0,015	0,03	0,03	0,5	256	256	4	64	1
92361	M	84	RP	Erysipel	Blood	10.05.2020	G	*stG2078.0*	0,015	0,03	0,03	0,5	0,12	0,12	4	0,5	1
92348	M	67	NW	no data	Blood	10.05.2020	C	*stG62647.0*	0,015	0,03	0,03	0,5	0,12	0,12	4	0,5	1
92240	M	65	SL	no data	Blood	25.04.2020	C	*stG62647.0*	0,015	0,03	0,03	0,5	2	0,12	4	0,5	1
92221	M	60	SH	Phlegmone	Blood	23.04.2020	C	*stG62647.0*	0,015	0,03	0,03	0,5	128	128	4	64	0,5
92211	F	87	NW	Sepsis	Blood	25.04.2020	C	*stG62647.0*	0,015	0,03	0,03	0,5	0,12	0,12	4	0,5	1
92199	n/a	72	RP	Phlegmone	Blood	15.04.2020	G	*stG3251.2*	0,015	0,015	0,015	0,5	0,12	0,12	4	0,5	0,5
92187	M	75	BY	Sepsis	Blood	20.04.2020	C	*stG62647.0*	0,015	0,015	0,03	0,5	0,12	0,12	4	0,5	0,5
92109	M	83	SN	no data	Blood	06.04.2020	G	*stG643.0*	0,015	0,03	0,03	0,5	0,12	0,12	4	0,5	0,5
92108	M	61	SN	sept. Arthritis, Komplikation d. Endoprothese	Gelenkpunktat	04.04.2020	G	*stG2078.10*	0,015	0,015	0,015	0,5	0,12	0,12	4	0,5	1
92107	M	88	RP	Sepsis	Blood	01.04.2020	C	*stG62647.0*	0,015	0,03	0,03	0,5	0,12	0,12	4	1	1
92087	M	84	SH	Pneumonie	Blood	03.04.2020	G	*stG6.1*	0,015	0,015	0,015	0,5	8	128	4	64	1
92075	F	90	RP	no data	Blood	03.04.2020	C	*stG62647.0*	0,015	0,03	0,03	0,5	0,12	0,12	4	0,5	1
92074	F	82	SL	no data	Blood	03.04.2020	G	*stG480.0*	0,015	0,015	0,015	1	0,12	0,12	4	32	1

92026	M	86	RP	Sepsis	Blood	25.03.2020	G	*stG485.0*	0,015	0,03	0,03	0,5	0,12	0,25	4	1	1
91963	M	81	RP	Sepsis	Blood	21.03.2020	C	*stG62647.0*	0,015	0,03	0,03	0,5	0,12	0,12	4	0,5	1
91958	F	81	NI	no data	Blood	19.03.2020	C	*stG62647.0*	0,015	0,015	0,015	0,5	0,12	0,12	4	0,5	1
91938	F	38	HE	no data	Blood	18.03.2020	C	*stG62647.0*	0,015	0,03	0,03	0,5	0,12	0,12	2	0,5	1
91915	M	83	NW	Sepsis	Blood	20.03.2020	G	*stG166b.0*	0,015	0,03	0,03	0,5	0,12	0,12	4	0,5	1
91914	M	78	NW	Sepsis	Blood	20.03.2020	G	*stG480.0*	0,015	0,03	0,03	0,5	0,12	0,12	4	0,5	1
91908	M	75	TH	Sepsis, Abszess	Blood	17.03.2020	G	*stC74a.0*	0,015	0,03	0,03	0,5	2	0,12	4	0,5	1
91829	M	62	RP	no data	Blood	11.03.2020	C	*stG62647.0*	0,015	0,03	0,03	0,5	0,12	0,12	2	0,25	1
91808	M	76	NW	no data	Blood	13.03.2020	C	*stG62647.0*	0,015	0,03	0,03	0,5	0,12	0,12	2	0,5	1
91787	M	82	NW	no data	Blood	11.03.2020	C	*stG62647.0*	0,015	0,03	0,03	0,5	0,12	0,12	4	1	1
91779	F	70	SN	Blutstrominfektion bei Dyspnoe	Blood	12.03.2020	G	*stG480.0*	0,015	0,015	0,015	0,5	0,12	0,12	4	0,5	1
91764	F	74	RP	no data	Gewebe Wirbelsäule	05.03.2020	C	*stG62647.0*	0,015	0,03	0,03	0,5	0,12	0,12	4	0,5	1
91732	F	83	SN	Sepsis	Blood	09.03.2020	C	*stG62647.0*	0,015	0,03	0,03	0,5	0,12	0,12	4	0,5	1
91731	M	66	HE	no data	Blood	03.03.2020	G	*stG245.0*	0,015	0,03	0,03	0,5	256	256	4	0,5	0,5
91611	M	66	BY	Osteomyelitis	Gewebe	03.03.2020	G	*stG485.0*	0,015	0,03	0,03	0,5	2	2	2	32	0,5
91610	F	67	BY	Sepsis, Phlegmone	Blood	04.03.2020	C	*stG62647.0*	0,015	0,03	0,03	0,5	0,12	0,12	2	0,5	1
91608	M	66	BE	Sepsis, Erysipel	Blood	28.02.2020	G	*stG2078.0*	0,015	0,03	0,03	0,5	0,12	0,12	4	0,5	1
91596	M	75	SH	acute exacerbation by bronchitis	Blood	29.02.2020	G	*stG6.1*	0,015	0,015	0,015	0,5	0,12	0,12	4	0,25	1
91526	M	73	SH	Psoas-Abszess	Blood	23.02.2020	G	*stG2078.0*	0,015	0,03	0,03	0,5	256	64	4	0,5	1
91487	M	64	SN	Fieber	Blood	27.02.2020	G	*stC74a.0*	0,015	0,03	0,03	0,5	0,12	0,12	4	4	0,5
91364	M	79	NW	Sepsis	Blood	21.02.2020	G	*stC5345.0*	0,015	0,03	0,03	0,5	0,12	0,12	4	0,5	0,5
91361	M	72	SN	Abszess Hüfte	Gelenkpunktat	22.02.2020	C	*stG62647.0*	0,015	0,015	0,03	0,5	0,12	0,12	4	0,5	1
91326	M	95	NW	no data	Blood	17.02.2020	C	*stG62647.0*	0,015	0,015	0,015	0,5	0,12	0,12	4	0,5	1
91309	M	84	NW	no data	Blood	16.02.2020	G	*stG485.0*	0,015	0,015	0,015	0,5	4	256	8	0,5	1
91300	M	86	HE	no data	Herzklappe	11.02.2020	C	*stG62647.0*	0,015	0,015	0,015	0,5	0,12	0,12	4	0,5	0,5
91287	M	60	SH	Sepsis, Pneumonie	Blood	12.02.2020	C	*stG62647.0*	0,015	0,015	0,015	0,5	0,12	0,12	2	0,5	0,5
91110	F	81	BW	Sepsis	Blood	07.02.2020	C	*stG62647.0*	0,015	0,015	0,015	0,5	2	0,12	4	1	1
91044	M	88	BY	infizierte Gangrän	Blood	02.02.2020	C	*stG485.0*	0,015	0,03	0,03	0,5	0,12	0,12	4	64	0,5
90987	M	66	SH	Pneumonie	Blood	29.01.2020	C	*stG62647.0*	0,015	0,03	0,03	0,5	0,12	0,12	4	0,5	1
90975	M	84	BY	Abszess Vorfuß	Swab wound	28.01.2020	G	*stG11.0*	0,015	0,015	0,03	0,5	0,12	0,12	4	0,5	1
90943	M	55	NW	no data	Blood	25.01.2020	C	*stG62647.0*	0,015	0,03	0,03	0,5	0,12	0,12	4	0,5	1
90925	M	66	BY	Nekrose am Fuß, Stauungsdermatitis	Swab wound	25.01.2020	C	*stG62647.0*	0,015	0,015	0,03	0,5	0,12	0,12	2	0,25	1
90892	M	76	BY	Ulzeration an den Füßen, arterielle Verschlusskrankheit	Swab wound	23.01.2020	G	*stG643.0*	0,015	0,015	0,03	0,5	0,12	0,12	2	32	1
90890	F	28	BY	Kaiserschnitt wegen Frühgeburt, Infektion	Swab intraoperativ	23.01.2020	C	*stG62647.0*	0,03	0,06	0,06	0,5	0,12	0,12	4	0,5	1
90887	F	69	SH	Ulcera beide Füße	Blood	23.01.2020	C	*stG62647.0*	0,015	0,03	0,03	0,5	0,12	0,12	4	0,5	1
90876	F	66	SN	Meningitis	Blood	24.01.2020	G	*stC74a.0*	0,015	0,015	0,015	0,5	0,12	0,12	2	0,5	1
90875	M	85	SN	Bakteriämie	Blood	22.01.2020	C	*stG62647.0*	0,015	0,015	0,03	0,5	0,12	0,12	4	0,5	1
90829	M	89	NW	no data	Blood	21.01.2020	G	*stC5345.0*	0,015	0,015	0,015	0,5	0,12	0,12	4	0,5	1
90726	M	46	SL	Erysipel	Blood	08.01.2020	G	*stC74a.0*	0,015	0,03	0,03	0,5	0,12	0,12	4	4	1
90674	F	69	NW	Sepsis	Blood	13.01.2020	G	*stG6.1*	0,015	0,015	0,015	0,5	0,12	0,12	4	0,5	0,5
90670	M	58	BY	unklarer Infekt	Blood	10.01.2020	G	*stG10.0*	0,015	0,015	0,03	0,5	0,12	0,12	4	64	1
90667	M	69	BY	Wundinfektion, Hautnekrose	Swab wound	05.01.2020	G	*stC74a.0*	0,015	0,015	0,015	0,5	0,12	0,12	2	4	1
90660	M	45	SN	Wundinfektion nach Kalotte-Implantat	Swab Kalotte	11.01.2020	G	*stC74a.0*	0,015	0,03	0,03	0,5	0,12	0,12	2	4	1
90654	M	80	NW	Sepsis	Blood	10.01.2020	G	*stG652.0*	0,015	0,015	0,03	0,5	0,12	0,12	4	0,25	0,5
90591	F	90	SN	Protheseninfektion	Gelenkpunktat	07.01.2020	C	*stG62647.0*	0,015	0,015	0,015	0,5	0,12	0,12	4	0,5	1
90474	F	80	SL	Sepsis	Blood	19.12.2019	G	*stG485.0*	0,015	0,015	0,015	0,5	0,12	0,12	4	0,5	1

90473	F	79	SL	Sepsis	Blood	19.12.2019	G	*stC74a.0*	0,015	0,015	0,015	0,5	0,12	0,12	4	4	0,5
90449	M	80	NW	no data	Blood	18.12.2019	G	*stG6.4*	0,015	0,015	0,015	0,5	0,25	0,12	4	32	1
90447	M	85	NW	no data	Blood	16.12.2019	G	*stC74a.0*	0,015	0,03	0,03	0,5	8	0,12	4	0,5	0,5
90424	F	85	BY	Erysipel am Auge, anaphylaktischer Schock	Blood	25.12.2019	C	*stG62647.0*	0,015	0,03	0,03	0,5	0,12	0,12	2	0,5	1
90410	M	67	NW	no data	Blood	28.12.2019	C	*stG62647.0*	0,015	0,015	0,015	0,5	0,12	0,12	4	0,5	1
90406	M	85	NW	no data	Blood	31.12.2019	C	*stG62647.0*	0,015	0,03	0,03	0,5	0,12	0,12	4	0,5	1
90363	M	89	SN	Sepsis	Blood	17.12.2019	C	*stG62647.0*	0,015	0,015	0,015	0,5	0,12	0,12	4	0,5	1
90362	F	57	SN	Arthritis	Gelenkpunktat	19.12.2019	G	*stG485.0*	0,015	0,015	0,03	0,5	0,12	0,12	2	8	1
90361	F	77	SN	Fieber	Blood	20.12.2019	G	*stG485.0*	0,015	0,015	0,03	0,5	0,12	0,12	2	0,5	1
90306	F	91	NW	no data	Blood	23.12.2019	G	*stG62647.0*	0,015	0,015	0,015	0,5	0,12	0,12	4	0,5	0,5
90220	F	78	SL	no data	Blood	11.12.2019	G	*stG643.0*	0,015	0,03	0,03	0,5	0,12	0,12	4	32	0,5
90197	M	58	BY	Wundinfektion, Abszess Unterarm	Swab intraoperativ	08.12.2019	C	*stG62647.0*	0,015	0,03	0,03	0,5	0,12	0,12	4	0,5	1
90194	F	65	BY	Sepsis	Swab wound	12.12.2019	G	*stG2078.0*	0,015	0,03	0,03	0,5	0,12	0,12	4	0,5	1
90148	M	63	SN	Sepsis	Blood	12.12.2019	G	*stG840.0*	0,015	0,03	0,03	0,5	0,5	0,12	4	8	0,5
90147	M	62	SN	Sepsis, Endokarditis	Blood	12.12.2019	C	*stG62647.0*	0,015	0,03	0,03	0,5	0,12	0,12	4	0,5	1
90102	M	81	SH	Sepsis	Blood	09.12.2019	C	*stG62647.0*	0,015	0,015	0,015	0,5	0,12	0,12	4	0,5	0,5
90041	F	83	BW	no data	Blood	04.12.2019	G	*stG652.1*	0,015	0,015	0,03	0,5	0,12	0,12	4	64	1
90003	F	73	SH	Infekt, COPD	Blood	02.12.2019	G	*stG485.0*	0,015	0,03	0,03	0,5	0,12	0,12	4	4	0,5
90001	F	85	BY	no data	Blood	07.12.2019	G	*stG6.1*	0,015	0,03	0,03	0,5	0,12	0,12	4	0,5	0,5
89986	M	59	NW	no data	Blood	05.12.2019	G	*stC5345.0*	0,015	0,015	0,015	0,5	0,12	0,12	4	0,5	1
89983	F	80	SN	no data	Blood	02.12.2019	G	*stG643.0*	0,015	0,03	0,03	0,5	0,12	0,12	4	32	1
89888	M	46	NW	Sepsis	Blood	01.12.2019	C	*stC1400.0*	0,015	0,015	0,015	0,5	256	0,12	4	8	1
89861	M	46	NW	no data	Blood	01.12.2019	C	*stC1400.0*	0,015	0,015	0,015	0,5	256	0,12	4	4	0,5
89859	M	34	NW	no data	Blood	28.11.2019	G	*stG485.0*	0,015	0,015	0,015	0,5	0,12	0,12	2	0,25	1
89851	M	50	BY	Sepsis, Unterschenkel Phlegmone	Blood	25.11.2019	C	*stG62647.0*	0,015	0,03	0,03	0,5	0,12	0,12	2	0,25	1
89814	F	41	SH	Sepsis, akutes Nierenversagen	Blood	24.11.2019	C	*stG62647.0*	0,015	0,015	0,015	0,5	0,12	0,12	4	0,5	1
89796	M	57	BY	Wundinfektion, Amputation des mittleren Zehs	Biopsie	20.11.2019	G	*stG245.0*	0,015	0,03	0,03	0,5	4	0,12	4	0,5	1
89749	F	78	RP	no data	Blood	17.11.2019	C	*stC839.0*	0,015	0,015	0,015	0,5	0,12	0,12	4	0,5	1
89741	M	67	BY	Wundinfektion, Amputation des großen Zehs	Swab wound	17.11.2019	C	*stG62647.0*	0,015	0,03	0,03	0,5	0,12	0,12	4	0,5	1
89727	M	72	NI	Sepsis, Erysipel	Blood	16.11.2019	G	*stG6.1*	0,03	0,03	0,03	0,5	0,12	0,12	2	0,25	2
89723	M	85	NW	no data	Blood	19.11.2019	C	*stG62647.0*	0,015	0,03	0,03	0,5	0,12	0,12	4	0,25	1
89700	F	40	BY	Aszites	Puncture Leber	12.11.2019	G	*stC74a.0*	0,015	0,015	0,015	0,5	0,12	0,12	4	0,5	1
89683	M	71	NW	Sepsis	Blood	14.11.2019	G	*stG2078.0*	0,015	0,015	0,015	0,5	0,25	0,12	4	0,5	1
89666	M	67	SN	Infekt Tep	Gelenkpunktat	09.11.2019	C	*stG62647.0*	0,015	0,03	0,03	0,5	0,25	0,12	4	0,5	1
89652	F	81	NW	no data	Blood	14.11.2019	G	*stG2078.0*	0,015	0,015	0,015	0,5	0,12	0,12	4	0,25	1
89651	M	63	NW	no data	Blood	14.11.2019	C	*stG62647.0*	0,015	0,03	0,03	0,5	0,25	0,12	4	0,5	1
89631	F	87	BY	no data	Blood	10.11.2019	G	*stG245.0*	0,015	0,03	0,03	0,5	16	0,12	4	32	0,5
89624	M	39	BY	Sepsis	Blood	05.11.2019	G	*stG166b.0*	0,015	0,03	0,03	0,5	0,12	0,12	4	0,5	1
89610	M	31	BW	Erysipel, Endokarditis	Blood	05.11.2019	G	*stC74a.0*	0,015	0,03	0,015	0,5	8	0,12	4	1	1
89587	F	84	RP	Sepsis	Blood	31.10.2019	G	*stC839.2*	0,015	0,03	0,015	0,5	0,12	0,12	2	32	1
89565	M	66	BB	eitrige Arthritis, Emypem	Gelenkpunktat	03.11.2019	C	*stG62647.0*	0,015	0,015	0,015	0,5	4	0,12	4	0,5	1
89558	M	31	BY	no data	Gelenkpunktat	31.10.2019	C	*stG62647.6*	0,015	0,015	0,015	0,5	0,12	0,12	4	0,5	0,5
89532	M	75	SH	Pneumonie, sept. Arthritis	Blood	30.10.2019	G	*stG6.0*	0,015	0,03	0,03	0,5	0,12	0,12	4	0,25	1
89497	M	78	SL	Wundinfekt	Blood	25.10.2019	G	*stG485.0*	0,015	0,03	0,03	0,5	0,12	0,12	4	2	1
89462	M	45	BB	Fieber unklare Ursache	Blood	24.10.2019	G	*stG480.0*	0,015	0,015	0,015	0,5	0,12	0,12	4	0,5	2
89461	F	78	BB	Erysipel	Blood	24.10.2019	G	*stG652.1*	0,015	0,015	0,015	0,5	0,12	0,12	4	64	1
89449	F	77	HE	no data	Blood	15.10.2019	C	*stG62647.0*	0,015	0,03	0,03	0,5	8	0,25	4	0,25	1
89448	M	65	HE	no data	Blood	14.10.2019	G	*stC6979.0*	0,015	0,03	0,03	0,5	0,12	0,12	4	0,25	1

89400	M	86	BY	Sepsis, Pneumonie	Blood	13.10.2019	C	*stG62647.0*	0,015	0,015	0,015	0,5	0,12	0,12	4	0,5	1
89387	F	90	NW	no data	Blood	15.10.2019	G	*stG6.3*	0,015	0,03	0,03	0,5	0,12	0,12	4	0,25	0,5
89373	F	94	SH	Erysipel	Blood	14.10.2019	C	*stG62647.0*	0,015	0,03	0,03	0,5	0,12	0,12	4	0,5	1
89362	M	81	SN	Erysipel	Blood	14.10.2019	C	*stG62647.0*	0,015	0,03	0,03	0,5	0,12	0,12	4	0,5	1
89271	M	46	NW	Phlegmone	Blood	06.10.2019	A	*stG652.0*	0,015	0,03	0,03	0,5	0,12	0,12	4	4	0,5
89250	M	93	SL	no data	Blood	01.10.2019	G	*stG245.0*	0,015	0,015	0,015	0,5	256	256	4	16	0,5
89249	M	71	RP	Endokarditis	Blood	30.09.2019	C	*stG62647.0*	0,015	0,015	0,015	0,5	0,12	0,12	4	0,5	1
89229	M	75	NW	fiberhafter Infekt	Blood	05.10.2019	C	*stG62647.0*	0,015	0,03	0,03	0,5	0,12	0,12	4	0,5	1
89226	M	91	SN	Fieber unbekannter Ursache	Blood	29.09.2019	G	*stG480.0*	0,015	0,015	0,015	0,5	0,12	0,12	2	0,5	1
89217	M	48	BY	Weichgewebeinfektion	Biopsie	28.09.2019	G	*stC839.2*	0,015	0,015	0,015	0,5	0,12	0,12	4	32	1
89214	F	52	BY	Sepsis	Blood	03.10.2019	G	*stC74a.0*	0,015	0,015	0,03	0,5	0,12	0,12	4	8	1
89184	M	57	BY	Erysipel Unterschenkel, Malum perforans Infektion Zeh	Biopsie wound	24.09.2019	C	*stG62647.0*	0,015	0,03	0,03	1	0,25	0,12	4	0,5	1
89175	M	70	NW	Fieber, multiple Wunden	Blood	30.09.2019	C	*stG62647.0*	0,015	0,03	0,03	0,5	0,25	0,12	4	0,5	1
89160	F	89	SN	others	Blood	25.09.2019	C	*stG62647.0*	0,015	0,015	0,015	0,5	0,12	0,12	4	0,5	1
89159	M	56	SH	no data	Blood	21.09.2019	G	*stG485.0*	0,015	0,015	0,015	0,5	0,12	0,12	4	0,5	1
89114	M	67	BY	Offene nässende Wunden	Swab wound	18.09.2019	G	*stG485.0*	0,015	0,03	0,015	0,5	0,12	0,12	4	16	4
89109	M	54	NW	no data	Blood	21.09.2019	G	*stG6.3*	0,015	0,015	0,015	0,5	256	0,12	4	32	1
89098	M	64	NW	no data	Blood	21.09.2019	G	*stG485.0*	0,015	0,03	0,03	0,5	0,12	0,12	4	64	1
89049	M	0	SN	no data	Blood	13.09.2019	C	*stG62647.0*	0,015	0,015	0,015	0,5	0,12	0,12	4	0,5	1
89022	M	84	SH	Sepsis	Blood	05.09.2019	G	*stG2078.0*	0,015	0,015	0,015	0,5	0,12	0,12	4	0,5	0,5
88866	M	76	SH	Sepsis, Endokarditis	Blood	28.08.2019	G	*stC5345.0*	0,015	0,03	0,03	0,5	0,12	0,12	4	0,5	1
88862	F	68	NW	no data	Blood	30.08.2019	C	*stG62647.0*	0,015	0,015	0,015	0,5	0,12	0,12	4	0,5	1
88856	F	59	SN	Sepsis	Blood	26.08.2019	G	*stC839.2*	0,015	0,015	0,015	0,5	0,12	0,12	4	0,5	1
88851	M	52	SH	Sepsis	Blood	26.08.2019	G	*stC839.2*	0,015	0,03	0,03	0,5	0,12	0,12	4	16	1
88850	M	58	SH	Sepsis, Pneumonie	Blood	27.08.2019	C	*stG62647.0*	0,015	0,015	0,015	0,5	8	0,25	4	0,25	1
88842	M	72	NW	Sepsis	Blood	24.08.2019	G	*stG6.1*	0,015	0,03	0,03	0,5	0,12	0,12	4	0,25	1
88840	M	79	NW	no data	Blood	28.08.2019	C	*stG62647.0*	0,015	0,03	0,03	0,5	0,12	0,12	4	0,5	1
88838	F	67	HE	Erysipel	Blood	21.08.2019	G	*stC74a.0*	0,015	0,015	0,015	0,5	256	256	4	64	0,5
88830	F	58	NW	no data	Blood	23.08.2019	G	*stC839.2*	0,015	0,03	0,03	0,5	0,12	0,12	4	32	1
88823	F	88	HB	Sepsis	Blood	22.08.2019	C	*stG62647.0*	0,015	0,015	0,03	0,5	0,12	0,12	4	0,25	2
88784	F	81	NW	no data	Blood	16.08.2019	G	*stC74a.0*	0,015	0,03	0,03	0,5	16	0,12	4	0,5	0,5
88783	M	85	NW	Sepsis	Blood	15.08.2019	G	*stG485.0*	0,015	0,015	0,03	0,5	0,12	0,12	4	0,5	1
88771	M	66	SH	Pneumonie	Blood	15.08.2019	C	*stG62647.0*	0,015	0,03	0,03	0,5	8	4	4	0,25	0,5
88764	F	56	BY	Fettgewebsnekrose	Biopsie	08.08.2019	G	*stG485.0*	0,015	0,03	0,03	0,5	0,12	0,12	4	0,25	1
88763	M	64	RP	Erysipel	Blood	11.08.2019	G	*stG485.0*	0,015	0,03	0,03	0,5	0,12	0,12	4	2	1
88760	M	73	NW	no data	Blood	15.08.2019	G	*stG6.0*	0,015	0,015	0,015	0,5	0,12	0,12	4	16	1
88753	M	55	SH	Sepsis, Erysipel	Blood	11.08.2019	C	*stG62647.0*	0,015	0,03	0,03	0,5	0,12	0,12	4	0,25	1
88746	M	86	BY	unklarer Infekt	Blood	08.08.2019	G	*stG166b.0*	0,03	0,06	0,03	0,5	0,12	0,12	2	0,5	0,5
88745	M	43	SH	Sepsis, Pneumonie	Blood	09.08.2019	C	*stG62647.0*	0,015	0,015	0,015	0,5	0,12	0,12	4	0,25	1
88744	M	58	NW	Sepsis	Blood	11.08.2019	G	*stG840.0*	0,015	0,03	0,03	0,5	0,12	0,12	4	0,5	1
88720	F	83	NW	Sepsis	Blood	21.07.2019	G	*stG485.0*	0,015	0,03	0,03	0,5	0,25	0,12	4	4	1
88702	F	90	SH	Sepsis, Harnwegsinfektion	Blood	03.08.2019	G	*stC839.2*	0,015	0,015	0,015	0,5	0,12	0,12	4	64	1
88696	F	83	NW	no data	Blood	03.08.2019	C	*stG62647.0*	0,015	0,03	0,03	0,5	0,12	0,12	4	32	1
88685	M	78	BY	Sepsis, Erysipel Unterschenkel, Pneumonie, Wundinfektion	Blood	29.07.2019	C	*stG62647.0*	0,015	0,03	0,03	0,5	0,12	0,12	4	0,5	1
88683	M	57	SN	Sepsis	Blood	30.07.2019	G	*stC46.0*	0,015	0,03	0,03	0,5	0,12	0,12	4	0,5	0,5
88660	M	88	SL	Sepsis	Blood	25.07.2019	C	*stG62647.0*	0,015	0,015	0,015	0,5	0,12	0,12	2	0,25	1
88640	F	80	NW	Erysipel	Blood	30.07.2019	C	*stG62647.0*	0,015	0,03	0,03	0,5	1	0,12	4	0,5	1
88635	M	69	NW	Sepsis	Blood	29.07.2019	A	*stG485.0*	0,015	0,03	0,03	0,5	0,12	0,12	4	4	1
88625	F	83	BY	no data	Blood	22.07.2019	G	*stG6.0*	0,015	0,015	0,015	0,5	0,12	0,12	4	32	1
88619	M	68	SH	Sepsis, Erysipel	Blood	24.07.2019	C	*stG62647.0*	0,015	0,015	0,015	0,5	0,12	0,12	4	0,5	0,5

88612	F	42	BY	Weichgewebsinfektion, Pyoderma gangrenosum	Swab wound	21.07.2019	G	*stG6.1*	0,015	0,015	0,015	0,5	0,12	0,12	4	0,25	1
88607	F	83	BY	Dekubitus Gesäß	Swab wound	17.07.2019	C	*stG62647.0*	0,015	0,015	0,015	0,5	0,12	0,12	4	0,5	1
88589	M	74	RP	Sepsis	Blood	18.07.2019	C	*stG62647.0*	0,015	0,015	0,015	0,5	0,12	0,12	4	0,5	1
88562	M	83	NW	no data	Blood	15.07.2019	G	*stG643.0*	0,015	0,015	0,015	0,5	0,12	0,12	4	0,5	1
88546	M	92	NW	no data	Blood	13.07.2019	C	*stG62647.0*	0,015	0,03	0,015	0,5	0,12	0,12	2	0,5	1
88537	M	64	BY	Erysipel Unterschenkel	Blood	14.07.2019	C	*stG62647.0*	0,015	0,03	0,03	0,5	0,12	0,12	4	0,5	1
88505	F	85	SH	Sepsis	Blood	11.07.2019	G	*stG10.0*	0,015	0,015	0,015	0,5	2	0,12	4	32	1
88491	M	70	RP	Phlegmone	Blood	05.07.2019	G	*stC839.2*	0,015	0,015	0,015	0,5	0,12	0,12	4	32	1
88250	M	77	SH	Sepsis, Erysipel	Blood	06.07.2019	C	*stG62647.0*	0,015	0,015	0,03	0,5	0,12	0,12	2	0,5	1
87911	M	53	SN	sept. Arthritis	Gelenkpunktat	26.06.2019	C	*stG62647.0*	0,015	0,015	0,015	0,5	0,12	0,12	2	0,5	1
87908	M	74	HE	no data	Blood	27.06.2019	G	*stC839.2*	0,015	0,03	0,03	0,5	0,12	0,12	4	64	1
87877	M	84	NW	no data	Blood	25.06.2019	G	*stG480.0*	0,015	0,015	0,015	0,5	0,12	0,12	4	0,5	0,5
87808	M	70	SH	Sepsis	Blood	24.06.2019	G	*stC74a.0*	0,015	0,015	0,015	0,5	0,12	0,12	4	4	0,5
87790	F	82	BY	no data	Blood	21.06.2019	A	*stG485.0*	0,015	0,03	0,03	0,5	0,12	0,12	4	8	0,5
87784	F	81	SH	no data	Blood	24.06.2019	G	*stG485.0*	0,015	0,015	0,015	0,5	0,12	0,12	4	0,5	1
87782	M	14	NW	no data	Blood	24.06.2019	A	*stG485.0*	0,015	0,03	0,03	0,5	0,12	0,12	4	8	1
87751	M	78	BY	Erysipel Unterschenkel	Blood	17.06.2019	G	*stG485.0*	0,015	0,015	0,015	0,5	0,12	0,12	4	0,5	1
87746	M	82	NW	Sepsis	Blood	18.06.2019	G	*stG652.18*	0,015	0,015	0,015	0,5	0,12	0,12	4	16	0,5
87707	M	61	NW	COPD	Blood	17.06.2019	C	*stG62647.0*	0,015	0,015	0,015	0,5	0,12	0,12	4	0,25	1
87691	F	81	NW	Sepsis	Blood	15.06.2019	G	*stG840.0*	0,015	0,03	0,03	0,5	0,12	0,12	4	4	1
87690	M	70	NW	Sepsis	Blood	14.06.2019	G	*stC74a.0*	0,015	0,03	0,03	0,5	0,12	0,12	4	8	1
87680	M	78	BY	Erysipel	Kniefragment	10.06.2019	G	*stG245.0*	0,015	0,03	0,03	0,5	256	256	4	16	1
87678	F	69	NW	Pneumonie	Blood	14.06.2019	C	*stG62647.0*	0,015	0,015	0,015	0,5	0,12	0,12	2	0,5	0,5
87651	M	75	RP	Erysipel	Blood	07.06.2019	G	*stG2078.0*	0,015	0,015	0,015	0,5	256	256	4	0,5	1
87649	M	89	RP	Sepsis, Erysipel	Blood	07.06.2019	G	*stC74a.0*	0,015	0,015	0,015	0,5	8	0,12	4	1	1
87581	M	64	NW	Sepsis	Blood	02.06.2019	C	*stG62647.4*	0,015	0,03	0,03	0,5	0,12	0,12	4	0,5	1
87569	M	80	NW	Sepsis	Blood	05.06.2019	G	*stG6.1*	0,015	0,03	0,03	0,5	0,12	0,12	4	0,5	0,5
87559	F	46	RP	no data	Blood	28.05.2019	C	*stG62647.5*	0,015	0,03	0,03	0,5	0,12	0,12	4	0,5	1
87558	F	50	RP	no data	Blood	29.05.2019	G	*stG245.0*	0,015	0,03	0,03	0,5	4	1	4	8	0,5
87557	F	89	RP	Pneumonie	Blood	29.05.2019	C	*stG62647.0*	0,015	0,03	0,03	0,5	8	0,25	4	0,5	1
87527	M	87	NW	no data	Blood	26.05.2019	C	*stG62647.0*	0,015	0,03	0,03	0,5	0,12	0,12	4	0,5	1
87469	M	80	RP	Phlegmone	Blood	21.05.2019	G	*stC74a.0*	0,015	0,015	0,015	0,5	8	0,12	4	0,5	1
87454	F	70	BB	no data	Blood	22.05.2019	C	*stG62647.0*	0,015	0,03	0,03	0,5	0,12	0,12	4	0,5	1
87442	M	76	SH	Sepsis, Pneumonie	Blood	25.05.2019	C	*stG62647.0*	0,015	0,015	0,015	0,5	0,12	0,12	4	0,5	1
87425	F	64	BY	Sepsis	Swab wound	19.05.2019	C	*stG62647.0*	0,015	0,015	0,015	0,5	0,12	0,12	4	0,5	1
87410	M	58	NI	Sepsis	Blood	20.05.2019	G	*stG485.0*	0,015	0,015	0,015	0,5	0,12	0,12	4	0,5	1
87389	F	79	SH	Sepsis, Erysipel	Blood	19.05.2019	G	*stG485.0*	0,015	0,015	0,015	0,5	0,12	0,12	2	0,5	1
87376	F	32	BY	Mastitis puerperalis	Swab wound	14.05.2019	C	*stG62647.0*	0,015	0,015	0,015	0,5	0,12	0,12	4	0,5	1
87374	M	59	SL	Sepsis, Erysipel	Blood	15.05.2019	C	*stG62647.0*	0,015	0,015	0,015	0,5	0,12	0,12	4	0,5	1
87325	M	70	BY	Erysipel	Blood	15.05.2019	G	*stG6.0*	0,015	0,015	0,015	1	0,12	0,12	4	0,5	0,5
87324	M	62	BY	Abszess	Swab wound	11.05.2019	G	*stG480.0*	0,015	0,015	0,015	0,5	0,12	0,12	4	64	0,5
87305	F	79	NW	Sepsis	Blood	13.05.2019	G	*stC839.2*	0,015	0,015	0,03	0,5	0,12	0,12	4	32	1
87192	M	77	HE	no data	Blood	18.04.2019	A	*stG652.0*	0,015	0,03	0,03	0,5	0,12	0,12	4	16	1
87180	F	88	NW	no data	Blood	05.05.2019	G	*stG6.1*	0,015	0,015	0,015	0,5	0,12	0,12	8	0,25	0,5
87138	M	75	NW	Sepsis	Blood	04.05.2019	G	*stG485.0*	0,015	0,03	0,03	0,5	0,12	0,12	4	8	0,5
87133	F	70	NW	Fieber	Blood	29.04.2019	C	*stG62647.0*	0,015	0,03	0,03	0,5	0,12	0,12	4	1	1
87132	M	86	NW	Pneumonie	Blood	29.04.2019	G	*stG2078.0*	0,015	0,015	0,03	0,5	0,12	0,12	4	1	1
87089	F	89	SN	sept. Arthritis, Exanthem	Blood	25.04.2019	C	*stG62647.0*	0,015	0,015	0,015	0,5	0,12	0,12	4	16	1
87087	M	87	SN	Fieber	Blood	27.04.2019	C	*stG62647.0*	0,015	0,015	0,03	0,5	0,12	0,12	4	0,5	1
87086	F	65	NW	Sepsis	Blood	01.09.2019	C	*stG62647.0*	0,015	0,015	0,015	0,5	0,12	0,12	4	0,25	1
87041	M	71	NW	Fieber	Blood	26.04.2019	G	*stG6.1*	0,015	0,015	0,015	0,5	0,12	0,12	4	0,5	0,5

87029	M	81	NW	Sepsis	Blood	26.04.2019	G	*stG643.0*	0,015	0,015	0,015	0,5	256	256	4	0,5	1
87023	F	88	NW	Sepsis	Blood	28.04.2019	C	*stG62647.0*	0,015	0,015	0,015	0,5	0,12	0,12	4	0,5	0,5
86814	F	88	NW	no data	Blood	15.04.2019	G	*stG10.0*	0,015	0,015	0,03	0,5	0,12	0,12	4	64	1
86778	M	67	HE	Erysipel, Infekt unklarer Genese	Blood	07.04.2019	G	*stC46.0*	0,015	0,03	0,03	0,5	0,12	0,12	4	0,5	0,5
86737	M	58	NW	Sepsis	Blood	11.04.2019	C	*stG62647.0*	0,015	0,03	0,03	0,5	0,12	0,12	4	0,5	1
86616	F	63	SH	Sepsis	Blood	01.04.2019	C	*stG62647.0*	0,015	0,015	0,015	0,5	4	0,12	4	0,5	0,5
86615	F	67	SH	Mastitis	Blood	03.04.2019	G	*stG2078.0*	0,015	0,03	0,03	0,5	0,12	0,12	4	1	1
86614	F	74	SH	Sepsis	Blood	04.04.2019	G	*stG6.8*	0,015	0,015	0,03	0,5	0,12	0,12	4	32	1
86539	M	58	SL	Sepsis	Blood	26.03.2019	C	*stG62647.0*	0,015	0,03	0,03	0,5	8	0,12	4	0,5	1
86529	F	89	SH	Sepsis	Blood	27.03.2019	G	*stG2078.0*	0,015	0,015	0,015	0,5	0,12	0,12	4	0,5	0,5
86488	M	70	SH	Sepsis	Blood	25.03.2019	C	*stG62647.0*	0,015	0,015	0,015	0,5	0,12	0,12	4	0,5	1
86410	M	84	NW	Sepsis	Blood	25.03.2019	G	*stC74a.0*	0,015	0,06	0,03	0,5	0,12	0,12	4	4	1
86399	M	75	BB	sept. Arthritis	Blood	21.03.2019	G	*stG6.1*	0,015	0,015	0,015	0,5	0,12	0,12	4	0,5	0,5
86397	M	83	NW	Sepsis	Blood	21.03.2019	G	*stG245.0*	0,015	0,03	0,03	0,5	0,12	0,12	4	16	0,5
86349	M	88	NW	Fieber	Blood	18.03.2019	G	*stG6.1*	0,015	0,03	0,03	0,5	0,12	0,12	4	0,25	0,5
86344	F	83	RP	Erysipel	Blood	13.03.2019	C	*stG62647.0*	0,015	0,015	0,015	0,5	0,12	0,12	4	0,25	1
86196	M	71	SH	Sepsis, Harnwegsinfektion	Blood	12.03.2019	C	*stG62647.0*	0,015	0,06	0,06	0,5	0,12	0,12	4	0,5	1
86182	M	84	HE	no data	Blood	07.03.2019	C	*stG485.0*	0,015	0,015	0,015	0,5	0,12	0,12	4	32	0,5
86148	F	55	NW	Fieber	Blood	10.03.2019	G	*stC74a.0*	0,015	0,015	0,03	0,5	0,12	0,12	4	4	0,5
86072	F	83	RP	Sepsis, Erysipel	Blood	05.03.2019	C	*stG62647.0*	0,015	0,015	0,015	0,5	0,12	0,12	4	0,5	1
86065	M	78	RP	Erysipel	Blood	03.03.2019	C	*stG62647.0*	0,015	0,015	0,015	0,5	0,12	0,12	2	0,5	1
85819	M	36	BY	no data	Blood	23.02.2019	G	*stG6.4*	0,015	0,015	0,015	0,5	0,12	0,12	4	0,25	0,5
85697	F	33	BY	Mastitis puerperalis	Swab Brust	11.02.2019	C	*stG62647.0*	0,015	0,015	0,015	0,5	0,12	0,12	4	0,5	0,5
85659	F	71	NW	unklares Fieber	Blood	18.02.2019	G	*stG2078.0*	0,015	0,015	0,015	0,5	0,12	0,12	4	0,5	0,5
85626	M	81	SH	Kniegelenkempyem	Blood	13.02.2019	C	*stG62647.0*	0,015	0,015	0,03	0,5	0,12	0,12	4	0,5	1
85625	M	90	SH	Sepsis, Pneumonie	Blood	14.02.2019	C	*stG62647.0*	0,015	0,015	0,015	0,5	0,12	0,12	4	0,5	0,5
85619	M	50	NW	Fieber	Blood	12.02.2019	C	*stG62647.0*	0,015	0,015	0,03	0,5	0,12	0,12	4	1	1
85609	F	62	BY	no data	Gelenkpunktat	14.02.2019	C	*stG62647.0*	0,015	0,015	0,03	0,5	0,12	0,12	4	0,5	1
85525	F	23	SN	Sepsis, STSS	Blood	13.02.2019	G	*stG840.0*	0,015	0,03	0,03	0,5	2	0,12	4	8	1
85473	F	73	NW	Sepsis	Blood	12.02.2019	G	*stG652.0*	0,015	0,03	0,03	0,5	0,12	0,12	4	128	0,5
85424	M	59	NW	no data	Blood	02.02.2019	G	*stG485.0*	0,015	0,03	0,03	0,5	0,12	0,12	2	8	0,5
85336	F	72	SH	Erysipel	Blood	01.02.2019	G	*stG480.0*	0,015	0,015	0,03	0,5	0,12	0,12	4	0,5	0,5
85334	F	87	SH	no data	Blood	03.02.2019	C	*stG62647.0*	0,015	0,03	0,03	0,5	0,12	0,12	4	0,5	1
85290	F	86	SH	Sepsis	Blood	29.01.2019	C	*stG643.1*	0,015	0,015	0,015	0,5	0,12	0,12	2	0,5	1
85264	M	79	NW	no data	Blood	28.01.2019	G	*stG6.1*	0,015	0,03	0,03	0,5	0,12	0,12	4	0,5	0,5
85245	M	80	BY	Fasciitis necroticans	Swab Achillessehne	24.01.2019	C	*stG62647.0*	0,015	0,03	0,03	0,5	0,12	0,12	2	0,5	1
85196	F	50	BY	Sepsis, Unterschenkel Phlegmone	Swab wound	25.01.2019	G	*stG480.0*	0,015	0,03	0,03	0,5	0,12	0,12	4	1	1
85175	F	78	SH	Harnwegsinfektion	Blood	23.01.2019	G	*stG6.1*	0,015	0,03	0,03	0,5	0,12	0,12	4	0,5	0,5
85167	M	84	SH	Sepsis, Erysipel	Blood	19.01.2019	C	*stG62647.0*	0,015	0,015	0,015	0,5	0,12	0,12	2	0,5	1
85166	M	87	SH	Sepsis, Erysipel	Blood	20.01.2019	C	*stG62647.0*	0,015	0,015	0,015	0,5	0,12	0,12	4	0,5	1
85164	M	52	BE	no data	Blood	31.10.2018	C	*stG643.0*	0,015	0,03	0,03	0,5	0,12	0,12	4	64	0,5
85154	M	73	NW	no data	Blood	24.01.2019	C	*stC74a.0*	0,015	0,03	0,03	0,5	4	0,12	4	0,5	1
85125	F	90	NW	Fieber	Blood	20.01.2019	C	*stG62647.4*	0,015	0,015	0,015	0,5	0,12	0,12	2	0,5	1
85122	F	90	NW	Pneumonie	Blood	17.01.2019	C	*stG485.0*	0,015	0,03	0,03	0,5	0,12	0,12	4	64	0,5
85087	F	68	BY	no data	Blood	18.01.2019	G	*stG485.0*	0,015	0,03	0,03	0,5	0,12	0,12	4	8	1
85069	F	81	BY	sept. Arthritis, Empyem linke Hüfte	Gelenkpunktat	15.01.2019	C	*stG62647.0*	0,015	0,015	0,015	0,5	0,12	0,12	4	0,5	1
85055	M	65	SN	Harnwegsinfekt	Blood	17.01.2019	G	*stG485.0*	0,015	0,015	0,03	0,5	0,12	0,12	4	2	0,5
85044	F	86	NW	Sepsis	Blood	13.01.2019	C	*stG62647.0*	0,015	0,03	0,03	0,5	0,12	0,12	2	0,5	1
85023	M	81	NW	no data	Blood	12.01.2019	G	*stG652.0*	0,015	0,015	0,03	0,5	0,12	0,12	4	64	1

85018	M	83	SN	Sepsis	Blood	12.01.2019	C	*stG62647.0*	0,015	0,015	0,015	0,5	4	0,12	4	0,5	1
84968	M	53	SN	Sepsis	Blood	07.01.2019	G	*stG485.0*	0,015	0,015	0,015	0,5	4	0,12	4	0,5	1
84933	M	75	NW	Sepsis	Blood	07.01.2019	G	*stG245.0*	0,015	0,03	0,03	0,5	0,12	0,12	2	0,5	0,5
84892	n/a	67	SN	Infektion Fuß	Gewebe foot	04.01.2019	G	*stG2078.11*	0,015	0,03	0,03	0,5	8	256	2	0,5	1
84889	M	84	SN	Fieber	Blood	06.01.2019	G	*stG10.0*	0,015	0,03	0,03	0,5	0,12	0,12	2	64	1
84885	M	73	NW	no data	Blood	08.01.2019	G	*stG480.0*	0,015	0,015	0,015	0,5	0,12	0,12	2	1	1
84773	M	78	SH	unklarer Infekt	Blood	02.01.2019	C	*stG62647.0*	0,015	0,03	0,03	0,5	0,12	0,12	4	1	1
84715	M	74	NW	no data	Blood	02.01.2019	G	*stG485.0*	0,015	0,03	0,03	0,5	0,12	0,12	4	0,5	1
84712	M	84	NW	no data	Blood	28.12.2018	G	*stG485.0*	0,015	0,03	0,015	0,5	0,12	0,12	4	8	0,5
84697	F	79	SH	Sepsis, Erysipel	Blood	25.12.2018	G	*stC74a.0*	0,015	0,03	0,03	0,5	0,12	0,12	4	8	0,5
84688	M	68	SH	no data	Blood	30.12.2018	G	*stC74a.0*	0,015	0,03	0,03	0,5	4	256	4	0,5	0,5
84657	F	50	NW	Sepsis	Blood	31.12.2018	C	*stG62647.0*	0,015	0,03	0,03	0,5	0,12	0,12	2	0,5	1
84616	M	71	RP	Phlegmone	Blood	22.12.2018	C	*stG62647.0*	0,015	0,015	0,03	0,5	0,12	0,12	4	0,5	0,5
84577	F	85	SN	Fieber	Blood	22.12.2018	C	*stG62647.0*	0,015	0,015	0,015	0,5	2	0,12	4	0,5	2
84576	F	88	SN	Fieber	Blood	25.12.2018	C	*stG62647.0*	0,015	0,015	0,03	0,5	0,12	0,12	4	0,5	1
84558	M	84	NW	no data	Blood	23.12.2018	C	*stG62647.0*	0,015	0,015	0,015	0,5	0,12	0,12	4	0,5	1
84494	M	89	HE	Sepsis	Blood	11.12.2018	G	*stG643.0*	0,015	0,03	0,03	0,5	0,12	0,12	4	64	0,5
84486	F	64	SH	Erysipel	Blood	18.12.2018	G	*stC839.2*	0,015	0,03	0,03	0,5	0,12	0,12	4	32	1
84333	M	53	SH	Sepsis	Blood	07.12.2018	G	*stG6792.16*	0,015	0,015	0,015	0,5	0,12	0,12	4	0,5	1
84277	M	69	BY	Sepsis, Ulcus am Fußballen	Swab wound	26.11.2018	C	*stG62647.0*	0,015	0,03	0,03	0,5	0,12	0,12	2	0,5	1
84255	M	81	BY	no data	Blood	28.11.2018	C	*stG62647.0*	0,015	0,03	0,03	0,5	0,12	0,12	4	0,5	1
84226	M	87	NW	no data	Blood	28.11.2018	C	*stG62647.0*	0,015	0,015	0,015	0,5	0,12	0,12	4	64	1
84173	F	87	BY	Sepsis	Implantat	26.11.2018	C	*stG62647.0*	0,015	0,015	0,03	0,5	0,12	0,12	2	0,5	1
84167	F	77	SH	Erysipel	Blood	24.11.2018	C	*stG62647.0*	0,015	0,015	0,015	0,5	0,12	0,12	4	1	1
84150	M	74	NW	Sepsis	Blood	23.11.2018	G	*stG166b.0*	0,015	0,03	0,03	0,5	0,12	0,12	4	1	0,5
84141	F	78	SN	Infekt	Blood	20.11.2018	G	*stG6.1*	0,015	0,03	0,03	0,5	0,12	0,12	2	32	0,5
84126	M	79	NW	Fieber	Blood	24.11.2018	G	*stC74a.0*	0,015	0,015	0,015	0,5	8	0,12	2	1	1
84113	F	46	BY	Sepsis, Fasciitis necroticans	Implantat	19.11.2018	G	*stG5420.0*	0,03	0,12	0,06	0,5	0,12	0,12	4	128	1
84051	M	31	NW	Sepsis	Blood	19.11.2018	C	*stG62647.0*	0,015	0,03	0,03	0,5	0,12	0,12	2	0,5	1
84021	F	93	SH	no data	Blood	11.11.2018	G	*stG485.0*	0,015	0,015	0,015	0,5	0,12	0,12	4	8	1
84016	M	83	HE	no data	Blood	30.10.2018	C	*stG62647.0*	0,015	0,015	0,015	0,5	0,12	0,12	4	0,5	1
84015	M	76	HE	no data	Blood	09.11.2018	C	*stG62647.0*	0,015	0,015	0,015	0,5	0,12	0,12	2	0,5	1
83994	F	83	NW	no data	Blood	09.11.2018	C	*stG62647.0*	0,015	0,015	0,015	0,5	0,12	0,12	2	0,5	1
83930	M	70	BY	no data	Blood	06.11.2018	G	*stG6.1*	0,015	0,015	0,03	0,5	8	256	4	64	1
83926	M	76	SH	Erysipel	Blood	06.11.2018	G	*stG480.0*	0,015	0,015	0,015	0,5	0,12	0,12	4	0,5	0,5
83925	M	80	SH	Erysipel	Blood	08.11.2018	C	*stG62647.0*	0,015	0,015	0,015	0,5	256	256	4	0,5	1
83857	F	79	SN	no data	Blood	20.10.2018	G	*stG485.0*	0,015	0,03	0,03	0,5	0,12	0,12	4	128	1
83742	F	88	HE	no data	Blood	15.10.2018	G	*stG485.0*	0,015	0,015	0,03	0,5	4	0,12	4	0,5	1
83729	F	89	NW	no data	Blood	21.10.2018	G	*stC839.2*	0,015	0,015	0,03	0,5	0,12	0,12	2	4	1
83668	M	43	NW	no data	Blood	16.10.2018	G	*stG2078.0*	0,015	0,015	0,015	0,5	0,12	0,12	4	0,5	1
83654	M	80	SH	Sepsis, Pneumonie	Blood	12.10.2018	G	*stG2078.0*	0,015	0,03	0,03	0,5	0,12	0,12	2	0,5	0,5
83638	M	69	NW	Sepsis	Blood	11.10.2018	C	*stG62647.0*	0,015	0,015	0,015	0,5	0,12	0,12	2	0,5	1
83576	M	78	SH	Sepsis, Erysipel	Blood	11.10.2018	C	*stG62647.0*	0,015	0,015	0,015	0,5	0,12	0,12	2	0,25	1
83516	F	65	NW	no data	Blood	06.10.2018	C	*stG62647.0*	0,015	0,015	0,015	0,5	0,12	0,12	4	0,25	1
83478	M	78	NW	Sepsis, Erysipel	Blood	28.09.2018	C	*stG62647.0*	0,015	0,03	0,03	0,5	0,12	0,12	4	0,25	1
83468	F	73	NW	no data	Blood	01.10.2018	G	*stG6.0*	0,015	0,015	0,015	0,5	0,12	0,12	2	64	0,5
83454	F	81	BY	Sepsis	Blood	22.09.2018	G	*stC74a.0*	0,015	0,015	0,015	0,5	0,12	0,12	4	64	0,5
83436	M	69	NW	no data	Blood	21.09.2018	C	*stC839.0*	0,015	0,015	0,015	0,5	0,12	0,12	4	0,25	0,5
83416	F	86	NW	Sepsis, Pneumonie	Blood	01.10.2018	C	*stG62647.0*	0,015	0,03	0,03	0,5	0,12	0,12	4	0,5	1
83412	M	74	SN	Fieber	Blood	24.09.2018	C	*stG62647.0*	0,015	0,015	0,03	0,5	0,12	0,12	4	0,5	0,5
83338	F	88	SN	no data	Blood	18.09.2018	G	*stG643.0*	0,015	0,015	0,03	0,5	0,12	0,12	2	32	1
83305	F	80	NW	Sepsis	Blood	15.09.2018	C	*stG62647.0*	0,015	0,015	0,015	0,5	0,12	0,12	4	0,25	2

83300	M	63	SH	Erysipel	Blood	12.09.2018	C	*stG62647.0*	0,015	0,03	0,03	0,5	0,12	0,12	4	0,5	1
83292	F	83	NW	no data	Blood	11.09.2018	G	*stG480.0*	0,015	0,03	0,03	0,5	0,12	0,12	4	0,5	1
83285	M	78	BY	Sepsis	Blood	10.09.2018	C	*stG62647.0*	0,015	0,03	0,03	0,5	0,12	0,12	4	0,5	1
83236	M	58	SN	Myopathie	Blood	04.09.2018	G	*stC74a.0*	0,015	0,015	0,015	0,5	2	0,12	4	0,5	1
83234	M	84	SH	no data	Blood	31.08.2018	C	*stG62647.0*	0,015	0,015	0,03	0,5	0,12	0,12	2	0,5	1
83222	F	84	BY	Gastroenteritis	Blood	08.09.2018	C	*stG62647.0*	0,015	0,03	0,03	0,5	0,12	0,12	4	0,5	1
83210	M	63	BY	Sepsis	Gelenkpunktat	25.08.2018	C	*stG62647.0*	0,015	0,03	0,03	0,5	0,12	0,12	4	0,5	1
83209	M	63	BY	Sepsis	Swab wound	28.08.2018	G	*stC839.8*	0,015	0,03	0,03	0,5	0,12	0,12	4	0,5	1
83186	M	50	NW	Sepsis	Blood	22.08.2018	C	*stG652.0*	0,015	0,03	0,03	0,5	0,12	0,12	4	1	0,5
83177	F	88	HE	Sepsis	Blood	27.08.2018	G	*stG245.7*	0,015	0,03	0,03	0,5	0,12	0,12	4	32	0,5
83168	F	86	NW	Sepsis	Blood	27.08.2018	G	*stG2078.0*	0,015	0,015	0,015	0,5	0,12	0,12	4	0,5	1
83152	M	84	NW	no data	Blood	26.08.2018	C	*stG62647.0*	0,015	0,03	0,03	0,5	0,12	0,12	4	0,5	1
83147	M	62	SN	Sepsis	Blood	23.08.2018	C	*stG62647.0*	0,015	0,015	0,015	0,5	0,12	0,12	4	0,5	1
83073	n/a	63	RP	Erysipel	Blood	11.08.2018	C	*stG62647.0*	0,015	0,03	0,03	0,5	0,12	0,12	2	0,25	1
83057	F	84	SH	Sepsis	Blood	08.08.2018	G	*stG485.0*	0,015	0,03	0,03	0,5	0,12	0,12	4	1	1
83005	F	71	SL	Erysipel	Blood	28.07.2018	G	*stC839.2*	0,015	0,015	0,03	0,5	0,12	0,12	4	32	1
83000	M	92	NW	no data	Blood	01.08.2018	C	*stG62647.0*	0,03	0,03	0,03	0,5	0,12	0,12	2	0,5	1
82993	F	82	SN	Fieber	Blood	30.07.2018	G	*stC74a.0*	0,015	0,015	0,015	0,5	8	0,12	2	0,5	0,5
82992	M	62	SN	Fieber	Blood	28.07.2018	G	*stC74a.0*	0,015	0,015	0,015	0,5	0,12	0,12	4	4	0,5
82991	M	56	SN	Endophtalmitis	Puncture	30.07.2018	G	*stG166b.0*	0,015	0,03	0,03	0,5	0,12	0,12	4	0,5	0,5
82978	F	69	BY	Phlegmone	Blood	27.07.2018	G	*stG2078.0*	0,015	0,03	0,03	0,5	2	0,12	2	0,5	0,5
82953	F	72	NW	no data	Blood	24.07.2018	C	*stG62647.0*	0,015	0,03	0,03	0,5	0,12	0,12	2	0,5	1
82943	M	51	SN	Erguss	Gelenkpunktat	24.07.2018	G	*stC74a.0*	0,015	0,03	0,03	0,5	0,12	0,12	4	64	1
82940	M	73	RP	Sepsis, Erysipel	Blood	24.07.2018	G	*stG6.0*	0,015	0,03	0,03	0,5	0,12	0,12	4	0,5	1
82928	F	80	SN	Sepsis	Blood	22.07.2018	C	*stG62647.0*	0,015	0,03	0,03	0,5	0,12	0,12	4	0,5	1
82913	F	66	HE	Erysipel	Blood	19.07.2018	G	*stC74a.0*	0,015	0,03	0,03	0,5	256	256	4	64	0,5
82909	M	72	HE	no data	Blood	18.07.2018	G	*stG10.0*	0,015	0,03	0,03	0,5	0,12	0,12	2	64	1
82858	M	83	SH	Fieber	Blood	15.07.2018	C	*stG62647.0*	0,015	0,015	0,03	0,5	0,12	0,12	2	0,5	1
82857	F	73	SH	Erysipel	Blood	14.07.2018	C	*stG62647.0*	0,015	0,03	0,03	0,5	256	256	4	0,5	1
82852	F	89	HE	Sepsis	Blood	15.07.2018	C	*stG62647.0*	0,015	0,03	0,03	0,5	0,12	0,12	2	0,5	1
82851	F	38	BY	no data	Blood	15.07.2018	G	*stG840.0*	0,015	0,03	0,03	0,5	0,12	0,12	4	8	1
82843	M	82	NW	Sepsis	Blood	17.07.2018	G	*stG2078.0*	0,015	0,015	0,015	0,5	0,12	0,12	2	0,5	1
82841	M	73	SH	Phlegmone	Blood	11.07.2018	C	*stG62647.0*	0,015	0,03	0,03	0,5	0,12	0,12	4	0,5	1
82840	M	79	SH	no data	Blood	12.07.2018	G	*stC74a.0*	0,015	0,03	0,03	0,5	0,12	0,12	4	64	1
82833	M	46	SN	Infekt	Blood	13.07.2018	C	*stG62647.0*	0,015	0,06	0,06	0,5	0,12	0,12	2	0,5	1
82820	F	80	NW	Sepsis	Blood	11.07.2018	C	*stG62647.0*	0,015	0,03	0,03	0,5	0,12	0,12	4	0,5	0,5
82789	F	38	SH	Phlegmone	Blood	07.07.2018	G	*stG480.0*	0,015	0,03	0,03	0,5	0,12	0,12	2	1	1
82766	M	74	HE	Erysipel	Blood	01.07.2018	G	*stC74a.0*	0,015	0,03	0,03	0,5	0,12	0,12	4	0,5	1
82764	M	74	NW	Fieber	Blood	04.07.2018	G	*stG245.0*	0,015	0,03	0,03	0,5	0,12	0,12	4	8	1
82747	M	67	BY	Erysipel	Swab wound	21.06.2018	G	*stC74a.0*	0,015	0,03	0,03	0,5	4	0,12	2	0,5	1
82725	F	81	NW	no data	Blood	26.06.2018	G	*stG480.0*	0,015	0,03	0,03	0,5	0,12	0,12	4	0,5	1
82724	M	73	NW	Sepsis	Blood	26.06.2018	C	*stC36.0*	0,015	0,015	0,015	0,5	0,12	0,12	2	0,5	1
82687	M	84	SH	Erysipel	Blood	19.06.2018	G	*stG480.6*	0,015	0,03	0,03	0,5	0,12	0,12	2	0,5	0,5
82671	M	62	SH	infiziertes Hämatom	Blood	18.06.2018	G	*stG10.0*	0,015	0,03	0,03	0,5	0,12	0,12	4	32	1
82654	M	82	RP	no data	Blood	13.06.2018	G	*stC5345.0*	0,015	0,015	0,015	0,5	0,12	0,12	2	0,5	1
82606	M	86	NW	Erysipel	Blood	14.06.2018	C	*stG62647.0*	0,015	0,03	0,03	0,5	0,12	0,12	2	0,5	1
82581	M	83	NW	no data	Blood	06.06.2018	G	*stC74a.0*	0,015	0,03	0,03	0,5	0,12	0,12	2	0,25	0,5
82578	M	58	RP	no data	Blood	03.06.2018	C	*stG62647.0*	0,015	0,03	0,03	0,5	0,12	0,12	2	0,25	1
82548	M	81	NW	Erysipel	Blood	01.06.2018	G	*stG485.0*	0,015	0,03	0,03	0,5	0,12	0,12	4	0,5	1
82511	M	83	HE	Sepsis	Blood	22.05.2018	G	*stG485.0*	0,015	0,015	0,015	0,5	0,12	0,12	4	0,5	1
82495	M	81	SH	Erysipel	Blood	31.05.2018	C	*stG62647.0*	0,06	0,06	0,06	0,5	0,12	0,12	4	16	0,5
82467	M	70	SH	Erysipel	Blood	26.05.2018	G	*stC74a.0*	0,015	0,03	0,03	0,5	0,12	0,12	4	4	0,5

82457	M	81	NW	no data	Blood	25.05.2018	G	*stG245.0*	0,015	0,015	0,03	0,5	256	256	4	16	0,5
82441	M	67	NW	Fieber	Blood	17.05.2018	A	*stG485.0*	0,015	0,03	0,03	0,5	0,12	0,12	4	16	1
82351	F	27	BY	Endometritis	Swab vagina	13.05.2018	C	*stG62647.0*	0,015	0,03	0,03	0,5	0,12	0,12	4	0,5	1
82336	F	43	SN	Infekt	Swab wound	14.05.2018	C	*stG62647.0*	0,015	0,03	0,03	0,5	0,12	0,12	4	0,5	0,5
82327	M	52	NW	no data	Blood	17.05.2018	G	*stG652.19*	0,015	0,015	0,015	0,5	0,12	0,12	4	32	0,5
82313	F	89	SH	Pneumonie	Blood	15.05.2018	G	*stG10.0*	0,015	0,015	0,03	0,5	0,12	0,12	4	64	1
82298	F	43	SN	sept. Arthritis	Swab Knie	14.05.2018	C	*stG62647.0*	0,015	0,03	0,03	0,5	0,12	0,12	4	0,5	0,5
82297	F	10	SN	sept. Arthritis	Puncture Kniegelenk	14.05.2018	G	*stG2078.0*	0,015	0,03	0,03	0,5	0,12	0,12	4	0,5	1
82289	M	73	SH	Sepsis	Blood	13.05.2018	C	*stG62647.0*	0,015	0,03	0,03	0,5	0,12	0,12	4	0,5	1
82254	M	42	HE	no data	Gewebe Aortenklappe	11.05.2018	G	*stG2078.0*	0,015	0,015	0,015	0,5	0,12	0,12	4	0,5	0,5
82193	M	59	RP	Endokarditis	Blood	06.05.2018	C	*stC74a.0*	0,015	0,03	0,03	0,5	2	0,5	4	64	0,5
82139	M	83	HE	Sepsis	Blood	25.04.2018	G	*stC74a.0*	0,015	0,03	0,03	0,5	0,12	0,12	4	4	0,5
82135	M	75	BY	Sepsis	Blood	29.04.2018	C	*stG62647.0*	0,015	0,015	0,015	0,5	0,12	0,12	4	0,5	2
82120	M	88	SN	no data	Blood	27.04.2018	G	*stG10.0*	0,015	0,03	0,03	0,5	0,12	0,12	2	64	1
82084	F	55	HE	Abszess	Swab wound	19.04.2018	G	*stC839.2*	0,015	0,015	0,03	0,5	0,12	0,12	2	64	1
82062	M	72	NW	no data	Blood	30.04.2018	C	*stG62647.0*	0,015	0,03	0,03	0,5	0,12	0,12	2	0,5	1
81996	M	78	BY	no data	Blood	19.04.2018	C	*stG62647.0*	0,015	0,03	0,03	0,5	0,12	0,12	2	0,5	1
81980	M	65	NW	no data	Blood	21.04.2018	C	*stG62647.0*	0,015	0,015	0,015	0,5	0,12	0,12	2	0,25	1
81975	F	74	RP	Sepsis	Blood	19.04.2018	C	*stG62647.0*	0,015	0,03	0,03	0,5	2	0,12	2	0,5	2
81954	M	47	BY	Phlegmone am Penis	Swab Harnröhre	13.04.2018	G	*stG5420.0*	0,015	0,03	0,03	0,5	0,12	0,12	2	32	1
81745	F	89	NW	Fieber	Blood	06.04.2018	G	*stG643.0*	0,03	0,03	0,03	0,5	0,12	0,12	4	64	0,5
81707	F	79	RP	Endokarditis	Blood	05.04.2018	C	*stG62647.0*	0,015	0,03	0,03	0,5	0,12	0,12	2	0,5	1
81587	F	74	SN	Sepsis, Phlegmone	Blood	01.04.2018	C	*stC1400.0*	0,015	0,03	0,03	0,5	0,12	0,12	4	8	1
81566	M	75	SH	Erysipel	Blood	30.03.2018	C	*stG62647.0*	0,015	0,03	0,03	0,5	0,12	0,12	2	0,5	1
81565	M	71	SH	sept. Arthritis	Blood	24.03.2018	C	*stG62647.0*	0,015	0,015	0,03	0,5	0,12	0,12	2	0,5	1
81564	F	79	SH	Sepsis	Blood	24.03.2018	G	*stG485.0*	0,015	0,03	0,03	0,5	0,12	0,12	2	0,5	1
81537	M	62	NW	Sepsis	Blood	31.03.2018	C	*stG62647.0*	0,015	0,03	0,03	0,5	256	256	2	64	1
81315	M	73	NW	Pneumonie	Blood	13.03.2018	G	*stG2078.0*	0,015	0,03	0,03	0,5	0,12	0,12	2	0,5	0,5
81295	M	73	NW	Spondilodiszitis	Swab intraoperativ	14.03.2018	A	*stG485.0*	0,015	0,03	0,03	0,5	0,12	0,12	4	4	1
81260	M	83	NW	no data	Blood	17.03.2018	G	*stG6.1*	0,015	0,03	0,03	0,5	0,12	0,12	2	1	0,5
81093	F	77	BY	no data	Blood	12.03.2018	G	*stG6.1*	0,015	0,03	0,03	0,5	16	16	4	1	0,5
81082	M	64	BY	sept. Schock, diab. Ulcera beider Füße	Swab wound	07.03.2018	C	*stG643.1*	0,015	0,03	0,03	0,5	0,12	0,12	4	0,5	1
81060	M	84	RP	Erysipel	Blood	06.03.2018	C	*stG643.1*	0,015	0,03	0,03	0,5	0,12	0,12	4	1	1
81059	F	80	BY	sept. Arthritis, Knieverletzung	Blood	09.03.2018	C	*stG62647.0*	0,015	0,03	0,03	0,5	0,12	0,12	2	1	0,5
80968	F	74	SN	no data	Blood	04.03.2018	C	*stC1400.0*	0,015	0,03	0,03	0,5	0,12	0,12	4	8	1
80909	M	45	NW	no data	Blood	06.03.2018	G	*stC5345.0*	0,015	0,03	0,03	0,5	0,12	0,12	4	1	1
80886	M	85	HE	no data	Blood	22.02.2018	A	*stG485.0*	0,015	0,03	0,03	0,5	0,12	0,12	4	16	1
80880	M	79	BY	no data	Blood	04.03.2018	G	*stG485.0*	0,015	0,03	0,03	0,5	0,12	0,12	2	2	1
80794	F	92	NW	Fieber	Blood	27.02.2018	G	*stG485.0*	0,015	0,015	0,03	0,5	0,12	0,12	4	1	1
80720	M	72	SN	no data	Blood	25.02.2018	G	*stG485.0*	0,015	0,03	0,03	0,5	0,12	0,12	4	8	1
80645	F	77	SL		Blood	21.02.2018	G	*stG485.0*	0,015	0,015	0,03	0,5	0,12	0,12	4	1	1
80624	M	52	NW	Meningitis	Blood	19.02.2018	C	*stG62647.0*	0,015	0,03	0,03	0,5	0,12	1	4	0,5	1
80596	M	60	SH	Erysipel	Blood	19.02.2018	G	*stC5345.0*	0,015	0,03	0,03	0,5	0,12	0,12	4	0,5	1
80558	F	88	SN	Sepsis, Erysipel	Blood	19.02.2018	C	*stG62647.0*	0,015	0,03	0,03	0,5	256	0,12	4	0,5	1
80547	F	92	NW	no data	Blood	19.02.2018	G	*stG2078.0*	0,015	0,03	0,03	0,5	0,12	0,12	4	0,5	0,5
80469	M	56	SH	Erysipel	Blood	15.02.2018	C	*stG62647.0*	0,015	0,015	0,015	0,5	0,12	0,12	4	0,5	1
80468	F	77	SH	Erysipel	Blood	15.02.2018	G	*stG480.0*	0,015	0,015	0,015	0,5	0,12	0,12	2	0,5	0,5
80453	F	73	SN	sept. Arthritis	Gelenkpunktat	14.02.2018	C	*stG62647.0*	0,015	0,015	0,015	0,5	0,12	0,12	2	0,5	1
80241	M	67	NW	no data	Blood	09.02.2018	C	*stG62647.0*	0,015	0,015	0,015	0,5	0,12	0,12	2	0,5	1

80235	M	87	NW	Sepsis, Wundinfekt	Blood	03.02.2018	C	*stG62647.0*	0,015	0,03	0,03	0,5	0,12	0,12	4	0,5	1
80191	M	67	BY	no data	Blood	04.02.2018	G	*stG480.0*	0,015	0,015	0,015	0,5	0,12	0,12	4	0,5	1
80075	M	59	SH	Erysipel	Blood	23.01.2018	G	*stC74a.0*	0,015	0,06	0,06	0,5	0,12	0,25	4	1	2
80063	F	80	NW	no data	Blood	29.01.2018	G	*stC74a.0*	0,015	0,03	0,03	1	0,12	0,12	4	64	1
80025	M	74	HE	no data	Blood	17.01.2018	C	*stG62647.0*	0,015	0,03	0,03	0,5	0,12	0,12	2	0,5	1
79932	M	75	SH	Pneumonie	Blood	16.01.2018	C	*stG62647.0*	0,015	0,03	0,03	0,5	256	256	4	0,5	1
79905	F	72	BY	no data	Blood	15.01.2018	C	*stG62647.0*	0,015	0,015	0,015	0,5	0,12	0,12	4	0,5	1
79886	F	79	SN	no data	Blood	12.01.2018	C	*stG62647.0*	0,015	0,015	0,015	0,5	0,12	0,12	4	0,5	1
79864	M	84	NW	no data	Gewebe Hüfte	12.01.2018	G	*stG2078.0*	0,015	0,03	0,03	0,5	0,12	0,12	4	128	1
79859	F	92	NW	no data	Blood	15.01.2018	C	*stG62647.0*	0,015	0,03	0,03	0,5	0,12	0,12	2	0,5	1
79832	M	69	SH	Erysipel	Blood	08.01.2018	G	*stG485.0*	0,015	0,03	0,03	0,5	0,12	0,12	4	2	1
79795	F	95	SN	Erysipel	Blood	08.01.2018	C	*stG62647.0*	0,015	0,015	0,03	0,5	0,12	0,12	2	0,5	1
79794	F	88	BY	Erysipel	Blood	08.01.2018	C	*stG62647.0*	0,015	0,03	0,03	0,5	0,12	0,12	4	0,5	1
79780	M	77	NW	Fieber	Blood	04.01.2018	C	*stG62647.0*	0,015	0,03	0,03	0,5	0,12	0,12	4	0,5	1
79779	M	91	NW	Fieber	Blood	03.01.2018	G	*stG245.0*	0,015	0,03	0,03	0,5	0,12	0,12	4	8	0,5
79778	F	86	NW	Fieber	Blood	03.01.2018	G	*stC74a.11*	0,015	0,015	0,015	0,5	0,12	0,12	2	8	0,5
79745	M	73	SH	Sepsis	Blood	03.01.2018	C	*stG62647.0*	0,015	0,03	0,03	0,5	0,12	0,12	4	0,5	1
79667	M	53	RP	Sepsis	Blood	02.01.2018	C	*stG62647.0*	0,015	0,015	0,015	0,5	0,12	0,12	2	0,5	8
79543	M	73	SN	sept. Arthritis	Blood	19.12.2017	G	*stG10.0*	0,015	0,015	0,015	0,5	0,12	0,12	4	64	0,5
79526	M	89	HE	Sepsis	Blood	15.12.2017	G	*stG652.1*	0,015	0,03	0,03	0,5	0,12	0,12	2	1	0,5
79515	M	70	SH	Sepsis	Blood	23.12.2017	G	*stC74a.0*	0,015	0,03	0,03	0,5	0,12	0,12	4	64	1
79486	F	79	NW	no data	Blood	29.12.2017	G	*stC74a.0*	0,015	0,03	0,03	0,5	0,12	0,12	2	1	1
79485	M	83	NW	no data	Blood	22.12.2017	G	*stC74a.0*	0,015	0,03	0,03	0,5	0,12	0,12	4	0,5	0,5
79430	M	57	NW	Sepsis	Blood	18.12.2017	G	*stG485.0*	0,015	0,03	0,03	0,5	4	0,12	4	64	0,5
79382	M	64	BY	Ischämie	Blood	17.12.2017	C	*stG62647.0*	0,015	0,03	0,03	0,5	0,12	0,12	4	0,5	1
79342	M	58	BY	no data	Blood	10.12.2017	G	*stG643.0*	0,015	0,03	0,03	0,5	0,12	0,12	4	64	0,5
79310	F	35	BY	no data	Swab wound	07.12.2017	G	*stG485.0*	0,015	0,03	0,03	0,5	0,12	0,12	4	2	1
79301	M	58	BY	no data	Blood	09.12.2017	G	*stG643.0*	0,015	0,03	0,03	0,5	0,12	0,12	4	64	0,5
79288	F	71	BY	Sepsis	Puncture Knie	05.12.2017	C	*stG62647.0*	0,015	0,03	0,03	0,5	0,12	0,12	2	0,5	1
79287	F	33	BY	Erysipel	Secretion Mamille	05.12.2017	C	*stG62647.0*	0,015	0,03	0,03	0,5	0,12	0,12	2	0,5	1
79258	F	79	SN	Sepsis	Blood	02.12.2017	G	*stC74a.0*	0,015	0,03	0,03	0,5	0,12	0,12	2	16	0,5
79225	M	68	SH	Fieber	Blood	01.12.2017	G	*stG485.0*	0,015	0,03	0,03	0,5	0,12	0,12	4	64	1
79220	F	79	NW	no data	Blood	05.12.2017	G	*stC74a.0*	0,015	0,03	0,03	0,5	0,12	0,12	4	0,5	1
79191	F	49	RP	Erysipel	Blood	29.11.2017	C	*stG62647.0*	0,015	0,03	0,03	0,5	0,12	0,12	4	0,5	1
79141	M	79	SH	Fieber unklarer Fokus	Blood	19.11.2017	G	*stG643.0*	0,015	0,03	0,03	0,5	0,12	0,12	4	64	1
79133	M	90	NW	Erysipel	Blood	25.11.2017	C	*stG62647.0*	0,015	0,03	0,03	0,5	0,12	0,12	4	1	1
79125	F	78	NW	no data	Blood	22.11.2017	G	*stG485.0*	0,015	0,03	0,03	0,5	0,12	0,12	4	0,5	1
79033	M	49	NW	Fieber	Blood	17.11.2017	G	*stG840.5*	0,015	0,03	0,03	0,5	0,12	0,12	4	8	0,5
78998	M	55	SN	Sepsis	Blood	15.11.2017	C	*stG62647.0*	0,015	0,03	0,03	0,5	0,12	0,12	4	0,5	1
78982	M	79	NW	Pneumonie	Blood	13.11.2017	C	*stG643.0*	0,015	0,03	0,03	0,5	8	1	2	0,5	1
78975	M	76	RP	Erysipel	Blood	11.11.2017	G	*stG643.0*	0,015	0,03	0,03	0,5	2	0,12	4	64	1
78971	M	74	BY	Erysipel	Blood	11.11.2017	C	*stG62647.0*	0,015	0,03	0,03	0,5	0,12	0,12	4	0,5	1
78958	M	59	NW	no data	Blood	13.11.2017	G	*stG652.0*	0,015	0,03	0,03	0,5	0,12	0,12	4	0,5	0,5
78920	M	77	SN	sept. Arthritis	Gelenkpunktat	09.11.2017	G	*stG10.10*	0,015	0,03	0,03	0,5	0,12	0,12	4	64	1
78902	M	76	SN	sept. Arthritis	Gelenkpunktat	06.11.2017	C	*stG62647.0*	0,015	0,03	0,03	0,5	0,12	0,12	4	0,5	1
78877	F	76	RP	no data	Blood	01.11.2017	C	*stG62647.0*	0,015	0,03	0,03		0,12	0,12	4	0,5	1
78769	n/a	69	NW	Sepsis	Blood	27.10.2017	C	*stG62647.0*	0,015	0,03	0,03	0,5	4	0,12	4	0,5	1
78683	M	75	NW	no data	Blood	18.10.2017	C	*stG62647.0*	0,015	0,03	0,03	0,5	0,12	0,12	4	0,5	1
78665	F	82	NW	no data	Blood	12.10.2017	G	*stG480.0*	0,015	0,015	0,015	0,5	0,12	0,12	4	0,5	1
78638	F	81	NW	Meningitis	Blood	11.10.2017	G	*stG485.0*	0,015	0,03	0,03	0,5	0,12	0,12	4	0,5	1
78635	M	62	SH	Phlegmone	Blood	09.10.2017	G	*stC74a.0*	0,015	0,03	0,03	0,5	0,12	0,12	2	0,5	1
78634	F	81	RP	Erysipel	Blood	10.10.2017	C	*stG62647.0*	0,015	0,015	0,015	0,5	0,12	0,12	4	0,5	1

78495	M	97	NW	Sepsis	Blood	01.10.2017	G	*stG245.0*	0,015	0,03	0,03	0,5	4	0,12	4	0,5	1
78487	M	57	BY	Sepsis, sept. Arthritis	Blood	01.10.2017	G	*stG643.1*	0,015	0,015	0,03	0,5	0,5	0,12	4	4	1
78452	M	76	NW	no data	Blood	28.09.2017	G	*stG480.0*	0,015	0,03	0,03	0,5	0,12	0,12	2	1	1
78364	M	76	BY	no data	Blood	14.09.2017	C	*stG485.0*	0,015	0,03	0,03	0,5	0,12	0,12	4	64	0,5
78346	M	73	NW	no data	Blood	12.09.2017	G	*stG485.0*	0,015	0,06	0,03	0,5	0,12	0,12	4	4	1
78340	F	61	NW	Sepsis, Pneumonie	Blood	14.09.2017	G	*stG2078.0*	0,015	0,03	0,03	0,5	0,12	0,12	4	1	1
78273	M	73	NW	no data	Blood	30.08.2017	G	*stC74a.0*	0,015	0,03	0,03	0,5	2	0,12	4	8	1
78270	M	79	SH	no data	Blood	01.09.2017	C	*stG62647.0*	0,015	0,03	0,03	0,5	0,12	0,12	4	0,5	1
78269	M	90	RP	Phlegmone	Blood	25.08.2017	G	*stC74a.0*	0,015	0,03	0,03	0,5	4	0,12	4	0,5	0,5
78249	M	88	NW	no data	Blood	26.08.2017	G	*stG2078.0*	0,015	0,03	0,03	0,5	0,12	0,12	4	0,5	1
78190	F	66	HE	Sepsis, Erysipel	Blood	21.08.2017	C	*stGM220.0*	0,015	0,03	0,03	0,5	0,12	0,12	4	8	1
77993	M	56	RP	Erysipel	Blood	12.08.2017	C	*stG62647.0*	0,015	0,015	0,03	0,5	0,12	0,12	2	0,5	1
77988	F	78	BY	Sepsis	Blood	16.08.2017	G	*stG485.0*	0,015	0,03	0,5	0,5	0,12	0,12	2	32	0,25
77944	F	46	BY	Erysipel	Blood	06.08.2017	C	*stG62647.0*	0,015	0,03	0,03	0,5	0,12	0,12	4	0,5	1
77915	F	73	NW	no data	Blood	02.08.2017	G	*stC5345.0*	0,015	0,03	0,03	0,5	0,12	0,12	4	1	2
77914	M	48	NW	Fieber	Blood	03.08.2017	G	*stC74a.0*	0,015	0,03	0,03	0,5	16	0,12	4	1	1
77913	M	70	NW	Fieber	Blood	02.08.2017	C	*stG62647.0*	0,015	0,03	0,03	0,5	16	256	4	1	2
77892	F	73	NW	Nierenversagen	Blood	26.07.2017	G	*stG62647.0*	0,015	0,03	0,03	0,5	0,12	0,12	2	1	1
77879	M	69	SN	no data	Puncture abcess	29.07.2017	A	*stG485.0*	0,015	0,03	0,03	0,5	0,12	0,12	4	16	1
77829	F	56	NW	no data	Blood	16.07.2017	G	*stC74a.0*	0,015	0,03	0,03	0,5	0,12	0,12	4	2	1
77826	F	83	SN	Sepsis, Erysipel	Blood	22.07.2017	C	*stG62647.0*	0,015	0,03	0,03	0,5	0,12	0,12	2	0,5	1
77814	F	86	BY	Erysipel	Blood	21.07.2017	G	*stG652.0*	0,015	0,03	0,03	0,5	0,12	0,12	4	128	1
77778	M	87	NW	Pneumonie	Blood	18.07.2017	C	*stG2574.3*	0,015	0,03	0,03	0,5	8	0,12	4	64	0,5
77777	M	76	NW	Erysipel	Blood	17.07.2017	C	*stG62647.0*	0,015	0,03	0,03	0,5	0,12	0,12	4	0,5	1
77775	M	37	SN	sept. Arthritis	Gewebe	19.07.2017	G	*stG10.0*	0,015	0,03	0,03	0,5	0,12	0,12	4	0,5	1
77765	M	79	NW	no data	Blood	22.07.2017	G	*stG2078.0*	0,015	0,03	0,03	0,5	0,25	0,25	2	0,5	1
77764	M	95	NW	Sepsis, Erysipel	Blood	21.07.2017	C	*stG62647.0*	0,015	0,03	0,03	0,5	0,12	0,12	4	0,5	1
77761	F	71	SH	no data	Blood	10.07.2017	G	*stG166b.0*	0,015	0,03	0,03	0,5	0,12	0,12	4	4	1
77760	M	80	RP	Erysipel	Blood	12.07.2017	G	*stG62647.0*	0,015	0,03	0,03	0,5	8	0,12	4	0,5	1
77755	M	83	NW	no data	Blood	14.07.2017	G	*stG480.0*	0,015	0,03	0,03	0,5	128	256	8	0,5	1
77720	F	82	SH	no data	Blood	07.07.2017	C	*stG62647.0*	0,015	0,03	0,03	0,5	0,12	0,12	4	0,5	1
77716	F	71	SH	no data	Blood	10.07.2017	G	*stG166b.0*	0,015	0,03	0,03	0,5	0,12	0,12	2	4	1
77711	M	91	NW	no data	Blood	06.07.2017	G	*stG245.0*	0,015	0,015	0,03	0,5	0,12	0,12	4	32	0,5
77672	M	78	SN	Sepsis	Blood	05.07.2017	G	*stG485.0*	0,015	0,03	0,03	0,5	0,12	0,12	4	0,5	1
77668	M	87	SH	Sepsis, Endokarditis	Blood	02.07.2017	G	*stG2078.0*	0,015	0,03	0,03	0,5	0,12	0,12	4	0,5	1
77647	M	84	SN	Fieber	Blood	28.06.2017	G	*stG62647.0*	0,015	0,03	0,03	0,5	0,12	0,12	4	1	1
77629	M	63	BY	no data	Blood	29.06.2017	G	*stG2078.0*	0,015	0,03	0,03	0,5	0,12	0,12	4	1	1
77627	F	79	NW	no data	Blood	01.07.2017	G	*stG6792.7*	0,015	0,03	0,03	0,5	256	0,12	4	0,25	1
77591	F	81	NW	no data	Blood	22.06.2017	G	*stG62647.0*	0,015	0,03	0,03	0,5	0,12	0,12	4	1	1
77578	F	58	SN	no data	Blood	20.06.2017	G	*stG2078.0*	0,015	0,03	0,03	0,5	0,12	0,12	2	0,5	0,5
77556	M	79	SH	Sepsis	Blood	17.06.2017	G	*stG485.0*	0,015	0,03	0,03	0,5	0,12	0,12	4	0,5	1
77543	M	88	NW	no data	Blood	20.06.2017	G	*stC5345.0*	0,015	0,03	0,03	0,5	0,12	0,12	4	0,5	1
77540	M	67	NW	no data	Blood	19.06.2017	C	*stG62647.0*	0,015	0,03	0,03	0,5	0,12	0,12	4	1	1
77493	M	73	HH	sept. Arthritis	Blood	09.06.2017	G	*stG166b.0*	0,015	0,03	0,03	0,5	0,12	0,12	4	0,5	1
77464	M	74	RP	Fieber	Blood	02.06.2017	G	*stC74a.0*	0,015	0,015	0,015	0,5	0,12	0,12	4	64	0,5
77462	M	50	SN	sept. Arthritis	Blood	09.06.2017	C	*stG62647.0*	0,015	0,03	0,03	0,5	0,12	0,12	4	0,5	1
77461	F	69	SN	Sepsis	Blood	08.06.2017	G	*stG485.0*	0,015	0,03	0,03	0,5	0,12	0,12	4	4	1
77414	M	85	SN	no data	Blood	29.05.2017	G	*stC74a.0*	0,015	0,03	0,03	0,5	0,12	0,12	4	0,5	8
77410	M	50	SN	no data	Gelenkpunktat	06.06.2017	C	*stG62647.0*	0,015	0,03	0,03	0,5	0,12	0,12	4	0,5	1
77366	M	60	NW	no data	Blood	28.05.2017	C	*stG62647.0*	0,015	0,03	0,03	0,5	0,12	0,12	4	32	1
77299	M	48	NW	Pneumonie	Blood	22.05.2017	G	*stG485.0*	0,015	0,015	0,015	0,5	4	0,12	4	32	0,5
77298	M	91	NW	no data	Blood	22.05.2017	G	*stC74a.0*	0,015	0,03	0,03	0,5	0,12	0,12	4	4	0,5

77256	M	68	HE	Sepsis	Blood	16.05.2017	G	*stG652.0*	0,015	0,015	0,015	0,5	256	256	4	32	1
77223	M	66	BY	Erysipel	Blood	15.05.2017	G	*stC74a.0*	0,015	0,03	0,03	0,5	0,12	0,12	4	0,5	0,5
77187	F	97	SH	Fasciitis necroticans	Swab wound	12.05.2017	G	*stG643.0*	0,015	0,03	0,03	0,5	0,12	0,12	4	64	1
77173	M	47	SH	Erysipel	Blood	13.05.2017	C	*stG62647.0*	0,015	0,03	0,03	0,5	0,12	0,12	4	1	1
77129	M	72	SH	Sepsis	Blood	09.05.2017	G	*stG6.1*	0,015	0,015	0,015	0,5	0,12	0,12	4	0,5	0,5
77094	M	18	SH	infiziertes Lymphangiom	Blood	07.05.2017	G	*stG643.0*	0,015	0,03	0,03	0,5	0,12	0,12	4	0,5	1
77093	M	82	SH	no data	Blood	05.05.2017	C	*stG62647.0*	0,015	0,03	0,03	0,5	0,12	0,12	4	0,5	1
77092	F	74	SN	Sepsis	Blood	08.05.2017	C	*stG62647.0*	0,015	0,015	0,03	0,5	0,12	0,12	2	0,5	1
76911	M	72	SL	no data	Blood	15.04.2017	G	*stC74a.0*	0,015	0,015	0,015	0,5	0,12	0,12	4	64	1
76892	M	45	ST	no data	Blood	05.04.2017	G	*stG485.0*	0,015	0,015	0,015	0,5	0,12	0,12	4	0,5	1
76749	F	89	NW	no data	Blood	19.04.2017	G	*stG480.0*	0,015	0,015	0,015	0,5	0,12	0,12	2	0,5	2
76721	M	63	SH	Sepsis, Erysipel	Blood	09.04.2017	G	*stG485.0*	0,015	0,03	0,03	0,5	4	0,12	4	8	0,5
76704	F	63	SH	Meningitis	Blood	06.04.2017	C	*stG62647.0*	0,015	0,03	0,03	0,5	0,12	0,12	4	0,5	1
76685	F	73	NW	no data	Blood	10.04.2017	C	*stG62647.0*	0,015	0,015	0,015	0,5	4	0,12	4	0,5	1
76583	M	70	NW	Pneumonie	Blood	04.04.2017	C	*stL1929.1*	0,015	0,015	0,015	0,5	0,12	0,12	4	4	1
76434	M	67	NW	no data	Blood	25.03.2017	G	*stG643.0*	0,015	0,03	0,03	0,5	0,12	0,12	4	0,5	1
76351	F	94	SN	Sepsis	Blood	25.03.2017	C	*stG62647.0*	0,015	0,015	0,015	0,5	0,12	0,12	4	1	0,5
76323	M	86	RP	Sepsis	Blood	24.03.2017	G	*stG643.0*	0,015	0,03	0,03	0,5	0,12	0,12	4	32	0,5
76252	F	73	HE	no data	Blood	16.03.2017	G	*stG10.0*	0,015	0,03	0,03	0,5	2	0,12	4	64	1
76238	M	80	SH	Sepsis	Blood	19.03.2017	G	*stG6.1*	0,015	0,015	0,015	0,5	0,12	0,12	4	0,5	0,5
76059	F	80	SN	sept. Arthritis, Endokarditis	Puncture knee	08.03.2017	G	*stG245.0*	0,015	0,03	0,03	0,5	0,12	0,12	4	64	1
75824	M	76	SL	Sepsis	Blood	24.02.2017	G	*stG6.1*	0,03	0,03	0,03	0,5	0,12	0,12	4	0,5	0,5
75654	M	60	HE	Sepsis	Blood	19.02.2017	C	*stG62647.0*	0,015	0,03	0,03	0,5	0,12	0,12	4	0,5	1
75632	M	80	HE	no data	Blood	15.02.2017	G	*stC74a.0*	0,015	0,015	0,015	0,5	0,12	0,12	4	8	1
75354	M	82	SN	no data	Blood	07.02.2017	G	*stC74a.15*	0,015	0,03	0,03	0,5	0,12	0,12	4	1	1
75241	F	79	SH	Erysipel	Blood	31.01.2017	C	*stG62647.0*	0,015	0,03	0,03	0,5	0,12	0,12	4	0,5	1
75213	F	69	NW	Infektion	Blood	01.02.2017	C	*stG62647.0*	0,015	0,015	0,015	0,5	0,12	0,12	4	1	2
75122	M	77	NW	no data	Blood	27.01.2017	C	*stG62647.0*	0,015	0,03	0,03	0,5	0,12	0,12	2	0,5	1
75058	F	83	SN	Sepsis, Pneumonie	Blood	25.01.2017	C	*stG62647.0*	0,015	0,03	0,03	0,5	0,12	0,12	4	0,5	0,5
75054	M	77	RP	Endokarditis, Sepsis	Blood	24.01.2017	G	*stG6792.0*	0,015	0,03	0,03	0,5	0,12	0,12	4	0,5	1
74960	M	62	SH	Sepsis	Blood	16.01.2017	C	*stG62647.0*	0,015	0,03	0,03	0,5	0,12	0,12	4	0,5	0,5
74955	M	71	NW	no data	Blood	21.01.2017	G	*stG62647.0*	0,015	0,03	0,03	0,5	0,12	0,12	2	0,5	1
74952	F	51	SN	Sepsis	Blood	16.01.2017	G	*stG2078.0*	0,015	0,03	0,03	0,5	0,12	0,12	4	0,5	1
74901	M	55	SH	Sepsis	Blood	13.01.2017	C	*stG62647.0*	0,015	0,03	0,03	0,5	0,12	0,12	4	0,5	1
74882	M	79	NW	Sepsis	Blood	16.01.2017	G	*stG2078.0*	0,015	0,03	0,03	0,5	0,12	0,12	4	1	1
74767	F	69	NW	no data	Blood	08.01.2017	G	*stG485.0*	0,015	0,03	0,03	0,5	0,12	0,12	4	8	1
74710	F	91	NW	Sepsis	Blood	08.01.2017	G	*stG2078.0*	0,015	0,03	0,03	0,5	0,12	0,12	4	0,5	1
74652	F	83	NW	no data	Blood	03.01.2017	C	*stG62647.0*	0,015	0,03	0,03	0,5	0,12	0,12	4	0,5	1
74651	M	66	NW	Sepsis, Pneumonie	Blood	25.12.2016	C	*stG62647.0*	0,015	0,03	0,03	0,5	0,12	0,12	4	0,5	1
74630	F	72	NW	no data	Blood	04.01.2017	C	*stG62647.0*	0,015	0,03	0,03	0,5	0,12	0,12	4	0,5	1
74610	F	73	BY	no data	Blood	31.12.2016	C	*stG62647.0*	0,015	0,03	0,03	0,5	0,12	0,12	4	0,5	1
74575	F	71	SH	Sepsis, Pneumonie	Blood	27.12.2016	G	*stG643.0*	0,015	0,03	0,03	0,5	0,12	0,12	4	32	1
74574	M	62	SH	Sepsis, Pneumonie	Blood	29.12.2016	C	*stG62647.0*	0,015	0,03	0,03	0,5	0,12	0,12	4	0,5	1
74573	M	74	SH	Sepsis	Blood	27.12.2016	C	*stG485.0*	0,015	0,015	0,015	0,5	0,12	0,12	4	64	1
74571	M	64	SH	Fieber	Blood	31.12.2016	C	*stG62647.0*	0,015	0,03	0,03	0,5	0,12	0,12	4	0,5	1
74495	M	70	BW	Sepsis	Blood	24.12.2016	C	*stG62647.0*	0,015	0,03	0,03	0,5	0,12	0,12	2	1	1
74449	M	43	BE	no data	Puncture Knie	23.12.2016	G	*stC74a.0*	0,015	0,03	0,03	0,5	0,12	0,12	4	0,5	1
74427	M	79	HE	Erysipel	Blood	18.12.2016	G	*stG480.0*	0,015	0,03	0,03	0,5	0,12	0,12	4	1	1
74346	M	78	NW	no data	Blood	20.12.2016	G	*stG245.0*	0,015	0,03	0,03	0,5	0,12	0,12	2	8	0,5
74104	M	62	SH	Pneumonie	Blood	28.11.2016	C	*stG62647.0*	0,015	0,03	0,03	0,5	0,12	0,12	4	0,5	1
74086	M	61	NW	Spondylochiditis, Epiderale Abszeß	Blood	02.12.2016	G	*stC5345.0*	0,015	0,015	0,015	0,5	0,12	0,12	4	1	1

74054	M	49	BY	Fasciitis necroticans	Swab wound	28.11.2016	C	*stG62647.0*	0,015	0,015	0,03	0,5	0,12	0,12	4	1	1
74051	M	64	ST	Sepsis	Blood	27.11.2016	C	*stG354.2*	0,015	0,015	0,015	0,5	0,12	0,12	2	0,5	1
74027	M	51	SH	acute exacerbation by bronchitis, Erysipel	Blood	21.11.2016	G	*stG2078.0*	0,015	0,03	0,03	0,5	0,12	0,12	4	1	1
74006	M	77	NW	no data	Blood	22.11.2016	G	*stG485.0*	0,015	0,03	0,03	0,5	4	0,12	4	1	1
74000	M	78	RP	sept. Arthritis	Blood	21.11.2016	C	*stG62647.0*	0,015	0,03	0,03	0,5	0,12	0,12	4	0,5	1
73937	M	78	SL	no data	Blood	01.11.2016	G	*stG6.1*	0,015	0,03	0,03	0,5	0,12	0,12	4	0,5	0,5
73871	M	57	RP	Erysipel	Blood	11.11.2016	C	*stG652.0*	0,015	0,03	0,03	0,5	0,12	0,12	4	2	0,5
73846	F	71	SN	Sepsis	Blood	11.11.2016	C	*stG62647.0*	0,015	0,03	0,03	0,5	0,12	0,12	4	0,5	1
73826	M	89	SN	no data	Blood	07.11.2016	G	*stC74a.0*	0,015	0,03	0,03	0,5	4	0,12	4	0,5	0,5
73819	M	53	SH	Infektion, Ekzem	Blood	03.11.2016	G	*stG652.18*	0,015	0,03	0,03	0,5	0,12	0,12	4	32	1
73742	M	61	SL	no data	Blood	29.10.2016	C	*stG62647.0*	0,015	0,015	0,015	0,5	0,12	0,12	4	1	2
73735	M	64	BY	Sepsis, Erysipel	Blood	30.10.2016	C	*stG62647.0*	0,015	0,015	0,03	0,5	0,12	0,12	4	0,5	1
73726	F	67	SN	no data	Blood	30.10.2016	C	*stG62647.0*	0,015	0,015	0,03	0,5	0,12	0,12	4	0,5	1
73637	M	84	SL	no data	Blood	24.10.2016	G	*stG485.0*	0,015	0,015	0,015	0,5	0,12	0,12	4	64	2
73631	M	70	NW	no data	Blood	25.10.2016	C	*stG62647.0*	0,015	0,03	0,03	0,5	0,12	0,12	4	1	1
73585	F	78	SL	Sepsis	Blood	17.10.2016	C	*stG62647.0*	0,015	0,03	0,03	0,5	2	0,12	2	0,5	1
73557	F	78	SL	no data	Blood	15.10.2016	C	*stG62647.0*	0,015	0,03	0,03	0,5	0,12	0,12	2	0,5	1
73494	F	87	SN	sept. Arthritis	Gelenkpunktat	10.10.2016	G	*stC74a.0*	0,015	0,03	0,03	0,5	0,12	0,12	4	4	1
73491	M	75	NW	no data	Blood	10.10.2016	G	*stG485.0*	0,015	0,03	0,03	0,5	0,12	0,12	4	0,5	1
73444	M	76	BW	Sepsis	Blood	02.10.2016	A	*stG485.0*	0,015	0,03	0,03	0,5	0,12	0,12	4	4	1
73434	F	60	HE	no data	Blood	30.09.2016	G	*stG2078.0*	0,03	0,03	0,03	0,5	0,12	0,12	4	0,5	0,5
73420	F	55	NW	no data	Blood	28.09.2016	C	*stG62647.0*	0,015	0,03	0,03	0,5	0,12	0,12	4	0,5	1
73314	M	78	BY	no data	Blood	23.09.2016	C	*stG62647.0*	0,015	0,03	0,03	0,5	0,12	0,12	4	0,5	1
73309	F	92	SH	Sepsis	Blood	20.09.2016	C	*stG62647.0*	0,015	0,015	0,015	0,5	0,12	0,12	4	0,5	1
73291	F	53	NW	no data	Blood	24.09.2016	G	*stG485.0*	0,015	0,015	0,015	0,5	2	0,12	4	32	0,5
73286	F	79	NW	Sepsis	Blood	20.03.2016	G	*stC74a.0*	0,015	0,015	0,015	0,5	0,12	0,12	4	8	0,5
73274	F	38	RP	V.a. Fasciitis necroticans	Blood	12.09.2016	G	*stG2078.0*	0,015	0,03	0,03	0,5	256	32	4	0,5	0,5
73258	F	90	NW	Sepsis	Blood	17.09.2016	G	*stC5345.0*	0,015	0,03	0,03	0,5	0,12	0,12	4	1	1
73251	M	76	HE	Sepsis, Fieber unklarer Genese	Blood	12.09.2016	G	*stC74a.0*	0,015	0,015	0,03	0,5	0,12	0,12	4	8	1
73225	F	75	HE	Sepsis	Blood	08.09.2016	C	*stG62647.0*	0,015	0,015	0,03	0,5	0,12	0,12	4	0,5	1
73220	M	89	NW	no data	Blood	09.09.2016	G	*stG485.0*	0,015	0,03	0,03	0,5	0,12	0,12	4	64	1
73216	F	71	NW	Sepsis	Blood	04.09.2016	G	*stG643.0*	0,015	0,015	0,015	0,5	0,12	0,12	4	0,5	0,5
73191	F	84	NW	Sepsis	Blood	02.09.2016	G	*stG6.1*	0,015	0,015	0,015	0,5	0,12	0,12	4	0,5	0,5
73188	M	67	SN	no data	Blood	02.09.2016	C	*stG2574*	0,015	0,015	0,015	0,5	0,12	0,12	4	0,5	1
73182	M	83	RP	Erysipel	Blood	29.08.2016	G	*stC74a.0*	0,015	0,015	0,015	0,5	0,12	0,12	2	0,5	1
73166	M	86	SN	no data	Blood	28.08.2016	C	*stG62647.0*	0,015	0,015	0,015	0,5	0,12	0,12	2	0,5	1
73151	n/a	45	HE	no data	Blood	26.08.2016	G	*stC74a.0*	0,015	0,03	0,03	0,5	4	0,12	4	0,5	0,5
73076	M	82	HE	no data	Blood	09.08.2016	G	*stG6.1*	0,015	0,03	0,03	0,5	0,12	0,12	4	0,5	0,5
72999	F	77	SL	no data	Blood	25.07.2016	G	*stC839.2*	0,015	0,03	0,03	0,5	0,12	0,12	4	64	2
72996	M	65	SN	sept. Arthritis	Gelenkpunktat	27.07.2016	G	*stC74a.0*	0,015	0,03	0,03	0,5	0,12	0,12	4	8	1
72985	M	79	HE	no data	Blood	20.07.2016	G	*stG480.0*	0,015	0,015	0,015	0,5	0,5	0,12	4	0,5	0,5
72952	F	48	NW	no data	Blood	20.07.2016	G	*stG840.0*	0,015	0,03	0,03	0,5	4	0,12	4	32	1
72945	M	57	NW	Sepsis	Blood	18.07.2016	G	*stG2078.0*	0,015	0,03	0,03	0,5	256	16	4	64	1
72940	M	56	RP	Mukositis	Blood	17.07.2016	G	*stC74a.0*	0,015	0,03	0,03	0,5	8	0,12	4	8	1
72936	F	79	SL	Sepsis	Blood	16.07.2016	G	*stG245.0*	0,015	0,015	0,015	0,5	0,12	0,12	4	16	1
72935	F	60	SL	Sepsis	Blood	20.07.2016	C	*stG62647.0*	0,015	0,03	0,03	0,5	0,12	0,12	4	0,5	1
72897	M	76	NW	no data	Blood	10.07.2016	G	*stG245.0*	0,015	0,03	0,03	0,5	0,12	0,12	4	32	0,5
72834	n/a	51	SN	Spandylodizitis	Swab intraoperativ	08.07.2016	C	*stG62647.0*	0,015	0,03	0,03	0,5	0,12	0,12	4	0,5	1
72796	F	68	NW	no data	Blood	23.06.2016	C	*stG62647.0*	0,015	0,03	0,03	0,5	0,12	0,12	4	0,5	1
72778	M	62	SN	Sepsis	Blood	25.06.2016	G	*stG10.0*	0,015	0,03	0,03	0,5	0,12	0,12	4	1	1
72698	M	89	RP	Erysipel	Blood	15.06.2016	G	*stC74a.0*	0,015	0,015	0,015	0,5	4	0,12	4	1	1

72696	M	93	NW	no data	Blood	22.06.2016	C	*stG62647.0*	0,015	0,015	0,015	0,5	0,12	0,12	2	0,5	1
72676	F	81	NW	no data	Blood	11.06.2016	C	*stG62647.0*	0,015	0,03	0,03	0,5	0,12	0,12	2	0,5	1
72644	M	77	NW	no data	Blood	08.06.2016	G	*stC74a.0*	0,015	0,03	0,03	0,5	0,12	0,12	4	0,5	0,5
72642	F	67	NW	no data	Blood	10.06.2016	G	*stG652.0*	0,015	0,03	0,03	0,5	0,12	0,12	4	0,5	0,5
72591	F	49	NW	no data	Blood	03.06.2016	G	*stC74a.0*	0,015	0,03	0,03	0,5	8	0,12	4	1	1
72578	F	72	NW	Erysipel	Blood	31.05.2016	G	*stG485.0*	0,015	0,015	0,015	0,5	0,12	0,12	4	1	1
72574	F	78	RP	Erysipel	Blood	28.05.2016	G	*stG6.1*	0,015	0,015	0,015	0,5	0,12	0,12	2	0,5	0,5
72532	F	85	HE	Sepsis	Blood	28.05.2016	C	*stG62647.0*	0,015	0,06	0,06	0,5	0,12	0,12	4	0,5	1
72461	M	49	BY	Fieber, atypische Blutung	Blood	24.05.2016	G	*stC74a.0*	0,015	0,03	0,03	0,5	4	0,12	4	0,5	1
72380	F	56	BY	Erysipel	Blood	17.05.2016	G	*stG6.1*	0,015	0,015	0,015	0,5	0,12	0,12	4	0,5	0,5
72379	M	74	BY	no data	Blood	18.05.2016	C	*stG62647.0*	0,015	0,015	0,015	0,5	0,12	0,12	2	0,5	0,5
72378		73	BY	no data	Blood	19.05.2016	G	*stG6.3*	0,015	0,03	0,03	0,5	0,12	0,12	4	0,5	0,5
72375	M	77	RP	Endokarditis	Blood	13.05.2016	G	*stG6.0*	0,015	0,015	0,015	0,5	8	0,12	4	128	1
72373	M	73	SH	Sepsis, Erysipel	Blood	19.05.2016	C	*stG62647.0*	0,015	0,03	0,03	0,5	0,12	0,12	4	1	1
72295	F	68	SH	Pneumonie	Blood	09.05.2016	C	*stG62647.0*	0,015	0,03	0,03	0,5	0,12	0,12	4	0,5	1
72268	M	83	SN	Sepsis, Phlegmone	Blood	09.05.2016	C	*stG62647.0*	0,015	0,015	0,015	0,5	0,12	0,12	4	0,5	1
72203	M	56	SN	H-TEP-Infektion	Puncture Gelenk	03.05.2016	C	*stG62647.0*	0,015	0,03	0,03	0,5	0,12	0,12	4	0,5	0,5
72169	F	51	NW	no data	Puncture Hüfte	03.05.2016	G	*stG2078.0*	0,015	0,015	0,015	0,5	0,12	0,12	4	0,5	1
72109	F	32	NW	no data	Blood	28.04.2016	G	*stC74a.0*	0,015	0,03	0,03	0,5	8	0,12	4	0,5	0,5
72037	F	85	NW	no data	Blood	23.04.2016	C	*stG62647.0*	0,015	0,03	0,03	0,5	0,12	0,12	4	0,5	1
71980	F	33	SN	Sepsis	Blood	12.04.2016	C	*stG62647.0*	0,015	0,03	0,03	0,5	0,12	0,12	4	0,5	1
71974	M	81	NW	no data	Blood	13.04.2016	G	*stG840.0*	0,015	0,03	0,03	0,5	0,12	0,12	4	0,5	0,5
71875	M	75	SN	sept. Arthritis	Puncture Gelenk	09.04.2016	G	*stG10.10*	0,015	0,03	0,03	0,5	0,12	0,12	4	64	1
71818	M	59	SH	Sepsis	Blood	30.03.2016	G	*stC74a.0*	0,015	0,015	0,015	0,5	0,12	0,12	4	32	8
71600	M	87	RP	Sepsis	Blood	16.03.2016	G	*stG480.0*	0,015	0,015	0,015	0,5	0,12	0,12	4	0,5	0,5
71564	M	49	SH	sept. Arthritis	Blood	17.03.2016	G	*stG10.4*	0,015	0,03	0,03	0,5	0,12	0,12	4	0,5	1
71356	F	58	BW	Sepsis, Phlegmone	Blood	07.03.2016	C	*stG62647.0*	0,015	0,03	0,03	0,5	0,12	0,12	2	0,5	1
71341	M	88	RP	no data	Blood	04.03.2016	C	*stG62647.0*	0,015	0,03	0,03	0,5	0,12	0,12	4	0,5	1
70821	M	65	SH	Sepsis	Blood	23.02.2016	C	*stG62647.0*	0,015	0,03	0,03	0,5	0,12	0,12	4	0,5	2
70805	M	87	NW	Sepsis	Blood	24.02.2016	G	*stC5345.0*	0,015	0,03	0,03	0,5	0,12	0,12	4	1	1
70595	F	75	SL		Blood	06.02.2016	G	*stG245.0*	0,015	0,03	0,03	0,5	0,12	0,12	4	32	0,5
70485	M	81	NW	no data	Blood	31.01.2016	G	*stG652.0*	0,015	0,03	0,03	0,5	0,12	0,12	4	32	0,5
70480	M	73	NW	no data, FUO	Blood	07.02.2016	G	*stC74a.0*	0,015	0,03	0,03	0,5	0,12	0,12	4	64	0,5
70479	F	89	NW	Sepsis	Blood	07.02.2016	C	*stG62647.0*	0,015	0,015	0,015	0,5	0,12	0,12	4	0,5	1
70474	M	83	HE	Sepsis	Blood	31.01.2016	G	*stG480.0*	0,015	0,03	0,03	0,5	0,12	0,12	4	0,5	0,5
70471	M	92	NW	Sepsis	Blood	27.01.2016	G	*stG643.0*	0,015	0,03	0,03	0,5	0,12	0,12	4	32	1
70433	F	20	HE	Sepsis	Blood	25.01.2016	G	*stG480.0*	0,015	0,03	0,03	0,5	0,25	0,12	4	0,5	2
70392	M	73	HE	Sepsis	Blood	24.01.2016	G	*stC74a.0*	0,015	0,03	0,015	0,5	16	0,12	4	0,5	1
70384	M	74	SN	Sepsis, sept. Arthritis	Blood	22.01.2016	G	*stG840.0*	0,015	0,03	0,03	0,5	16	256	8	8	0,5
70363	F	79	SL	no data	Blood	24.01.2016	G	*stG245.0*	0,015	0,03	0,03	0,5	0,25	0,12	4	64	1
70320	M	85	SN	no data	Puncture knee	22.01.2016	C	*stG62647.0*	0,015	0,03	0,03	0,5	0,12	0,12	4	0,5	1
70188	M	75	RP	Erysipel	Blood	10.11.2016	G	*stC839.2*	0,015	0,03	0,03	0,5	0,12	0,12	4	128	1
70061	M	60	BW	no data	Blood	04.01.2016	A	*stG485.0*	0,015	0,03	0,03	0,5	0,12	0,12	4	8	1
69979	M	59	HE	Sepsis	Blood	28.12.2015	C	*stG62647.0*	0,015	0,03	0,03	0,5	0,12	0,12	2	0,5	1
69773	F	53	BY	no data	Blood	17.12.2015	G	*stC74a.0*	0,015	0,03	0,03	0,5	4	0,12	4	8	0,5
69733	M	30	NW	Sepsis	Blood	18.12.2015	C	*stG485.0*	0,015	0,03	0,03	0,5	0,12	0,12	4	64	0,5
69706	M	75	SL	no data	Blood	11.12.2015	C	*stG62647.7*	0,015	0,03	0,03	0,5	0,12	0,12	4	1	1
69625	F	47	NW	no data	Blood	02.12.2015	C	*stG62647.0*	0,015	0,03	0,03	0,5	0,12	0,12	4	0,5	1
69622	M	75	HE	Erysipel	Blood	29.11.2015	G	*stG652.0*	0,015	0,03	0,03	0,5	0,12	0,12	4	0,5	0,5
69593	F	64	HE	Erysipel, Sepsis	Blood	26.11.2015	C	*stG62647.0*	0,015	0,03	0,03	0,5	0,12	0,12	4	0,5	1
69579	F	50	BY	Sepsis	Blood	25.11.2015	G	*stG245.0*	0,015	0,03	0,03	0,5	0,12	0,12	2	64	1
69578	F	61	BY	no data, Fieber	Blood	24.11.2015	C	*stG62647.0*	0,015	0,03	0,03	0,5	0,12	0,12	4	0,5	1

69549	F	90	NW	no data	Blood	25.11.2015	G	*stG2078.0*	0,015	0,03	0,03	0,5	0,12	0,12	2	1	1
69522	M	89	NW	no data	Blood	16.11.2015	G	*stG10.0*	0,015	0,03	0,03	0,5	0,12	0,12	2	64	1
69479	M	59	NW	no data	Blood	16.11.2015	C	*stG62647.0*	0,015	0,03	0,03	0,5	0,12	0,12	2	1	1
69450	M	47	BY	Sepsis, Erysipel	Blood	10.11.2015	G	*stG10.0*	0,015	0,03	0,03	0,5	0,12	0,12	4	1	1
69439	F	66	HE	no data	Blood	09.11.2015	G	*stG507.1*	0,015	0,03	0,03	0,5	0,12	0,12	4	0,5	0,5
69434	M	74	BY	Sepsis	Blood	11.11.2015	A	*stG652.0*	0,015	0,03	0,03	0,5	0,12	0,12	4	8	0,5
69410	n/a	88	NW	no data	Blood	10.11.2015	G	*stG10.11*	0,015	0,015	0,03	0,5	0,12	0,12	4	0,5	1
69375	M	81	NW	no data	Blood	08.11.2015	G	*stG6.0*	0,015	0,015	0,015	0,5	256	0,12	4	64	1
69324	M	75	NW	no data	Blood	03.11.2015	C	*stG62647.0*	0,015	0,03	0,03	0,5	0,12	0,12	4	1	1
69322	M	75	NW	Meningitis	CSF	03.11.2015	G	*stG245.0*	0,015	0,03	0,03	0,5	4	0,12	4	0,5	1
69285	F	83	NW	no data	Blood	28.10.2015	G	*stG10.0*	0,015	0,03	0,03	0,5	0,12	0,12	4	64	1
69282	F	54	NW	no data, Fieber	Blood	02.11.2015	G	*stG10.4*	0,015	0,03	0,03	0,5	0,12	0,12	4	64	1
69266	F	76	SL	no data	Blood	27.10.2015	G	*stG480.0*	0,015	0,03	0,03	0,5	128	256	8	1	1
69202	M	81	NW	pneumonia	Blood	25.10.2015	C	*stG62647.0*	0,015	0,03	0,03	0,5	0,12	0,12	2	0,5	1
69036	M	69	BY	Sepsis, gangän	Blood	05.12.2015	G	*stG643.0*	0,015	0,015	0,015	0,5	0,12	0,12	2	64	0,5
69029	M	85	NW	Sepsis	Blood	04.10.2015	G	*stG2078.0*	0,015	0,03	0,03	0,5	256	64	4	64	1
69002	M	73	RP	Erysipel	Blood	27.09.2015	G	*stC74a.0*	0,015	0,03	0,03	0,5	0,12	0,12	4	64	1
68916	F	54	NW	Sepsis	Blood	29.09.2015	G	*stC46.0*	0,015	0,03	0,03	0,5	8	0,12	4	8	1
68915	F	80	NW	Sepsis, Thoraxschmerzen	Blood	23.09.2015	C	*stG62647.0*	0,015	0,03	0,03	0,5	0,12	0,12	4	32	1
68867	F	62	RP	Sepsis	Blood	19.09.2015	G	*stG10.0*	0,015	0,03	0,03	0,5	0,12	0,12	2	64	1
68866	M	87	NW	Sepsis	Blood	17.09.2015	C	*stG62647.0*	0,015	0,03	0,03	0,5	0,12	0,12	4	16	1
68827	M	81	BY	no data	Blood	14.09.2015	G	*stG62647.0*	0,015	0,03	0,03	0,5	0,12	0,12	2	0,5	0,5
68814	M	80		Sepsis	Blood	02.09.2015	G	*stC74a.0*	0,015	0,015	0,015	0,5	0,25	0,12	4	64	0,5
68800	F	53	NW	no data	Blood	13.09.2015	G	*stG485.0*	0,015	0,03	0,03	0,5	0,12	0,12	2	64	1
68796		70	NW	Sepsis	Blood	05.09.2015	G	*stG643.0*	0,015	0,03	0,03	0,5	0,12	0,12	2	64	1
68785	M	36	NW	no data	Blood	03.09.2015	G	*stG2078.0*	0,015	0,03	0,03	0,5	0,12	0,12	4	0,5	1
68784	M	61	SN	sept. Arthritis, Bursitis	Gelenkpunktat	03.09.2015	G	*stG6.0*	0,015	0,015	0,015	0,5	16	256	2	32	0,5
68783	F	71	SN	Sepsis	Blood	07.09.2015	G	*stG10.0*	0,015	0,03	0,03	0,5	0,12	0,12	4	64	1
68770	M	65	NW	no data	Blood	02.09.2015	G	*stC74a.0*	0,015	0,03	0,03	0,5	0,12	0,12	4	8	0,5
68763	M	57	BW	Fasciitis necroticans, Sepsis, sept. Arthritis	Blood	31.08.2015	G	*stG245.0*	0,015	0,03	0,03	0,5	256	256	4	16	0,5
68717	M	31	SN	no data	Blood	25.08.2015	G	*stG6.4*	0,015	0,03	0,03	0,5	0,12	0,12	4	8	0,5
68681	M	74	RP	pneumonia, V.a. Endokarditis	Blood	17.08.2015	C	*stG62647.0*	0,015	0,03	0,03	0,5	0,12	0,12	2	0,5	1
68669	M	81	SN	pneumonia, Sepsis	Blood	15.08.2015	C	*stG62647.0*	0,015	0,03	0,03	0,5	0,12	0,12	4	0,5	0,5
68615	M	78	NW	no data	Blood	06.08.2015	G	*stG11.0*	0,015	0,015	0,015	0,5	0,12	0,12	4	0,5	0,5
68592	F	79	NW	no data	Blood	05.08.2015	C	*stG62647.0*	0,015	0,03	0,03	0,5	0,12	0,12	4	0,5	1
68576	F	82	NW	Erysipel, Fieber	Blood	28.07.2015	G	*stC5345.0*	0,015	0,03	0,015	0,5	0,12	0,12	4	0,5	1
68491	M	70	SN	Phlegmone	Gewebe	16.07.2015	C	*stG62647.0*	0,015	0,03	0,03	0,5	4	256	4	0,5	1
68452	M	53	RP	Erysipel	Blood	16.07.2015	G	*stC74a.0*	0,015	0,03	0,03	0,5	0,12	0,12	2	8	1
68443	F	88	SN	no data	Blood	17.07.2015	C	*stG62647.0*	0,015	0,03	0,03	0,5	64	128	4	0,5	1
68441	M	69	SN	no data	Blood	13.07.2015	C	*stG62647.0*	0,015	0,03	0,03	0,5	0,12	0,12	2	0,5	1
68399	M	50	SN	Bursitis	Puncture Ellenbogen	05.07.2015	G	*stC74a.0*	0,015	0,03	0,03	0,5	0,12	0,12	4	32	1
68242	M	46	RP	V.a. Endokarditis, Sepsis, sept. Arthritis	Blood	20.06.2015	G	*stG485.0*	0,015	0,015	0,015	0,5	0,12	0,12	4	0,5	1
68171	F	75	SN	Sepsis	Blood	18.06.2015	G	*stG245.0*	0,015	0,03	0,03	0,5	0,12	0,12	4	8	0,5
68153	F	77	SN	Erysipel, Sepsis	Blood	12.06.2015	C	*stG62647.0*	0,015	0,03	0,03	0,5	0,12	0,12	4	0,5	1
68109	M	73	SN	Erysipel, Sepsis	Blood	09.06.2015	G	*stG245.0*	0,015	0,03	0,03	0,5	0,12	0,12	4	16	0,5
68083	M	28	RP	Spondylitis	Puncture Wirbelkörper	06.06.2015	C	*stG62647.0*	0,015	0,03	0,03	0,5	0,12	0,12	4	0,5	0,5
67939	M	55	NW	no data	Blood	27.05.2015	G	*stG6.1*	0,015	0,03	0,03	0,5	0,12	0,12	2	32	0,5
67914	M	50	NW	no data	Blood	22.05.2015	C	*stG62647.0*	0,015	0,015	0,015	0,5	0,12	0,12	2	0,5	1

67822	F	93	NW	no data	Blood	19.05.2015	G	*stG166b.0*	0,015	0,03	0,03	0,5	0,12	0,12	4	4	0,5
67808	F	84		Endokarditis	Blood	16.05.2015	C	*stG62647.0*	0,015	0,03	0,03	0,5	0,12	0,12	4	0,5	1
67799	F	81	HE	no data	Blood	07.05.2015	G	*stC74a.0*	0,015	0,03	0,015	0,5	0,12	0,12	4	16	1
67725	M	75	NW	Sepsis	Blood	09.05.2015	C	*stG62647.0*	0,015	0,03	0,03	0,5	0,12	0,12	4	1	1
67691	M	46	SN	Sepsis	Blood	07.05.2015	G	*stC74a.0*	0,015	0,015	0,015	0,5	0,12	0,12	2	0,5	1
67567	M	79	HE	no data	Blood	20.04.2015	G	*stC74a.0*	0,015	0,03	0,03	0,5	1	0,12	4	64	1
67434	M	75	RP	Sepsis, STSS,V.a. Erysipel	Blood	13.04.2015	G	*stG485.0*	0,015	0,03	0,03	0,5	0,12	0,12	4	1	1
67384	M	66	SN	Sepsis	Blood	11.04.2015	G	*stC74a.0*	0,015	0,03	0,03	0,5	0,25	0,12	4	8	0,5
67383	M	42	SN	Sepsis	Blood	13.04.2015	G	*stG480.2*	0,015	0,015	0,015	0,5	0,12	0,12	4	0,5	1
67367	M	83		no data	Blood	14.04.2015	G	*stG485.0*	0,015	0,03	0,03	0,5	0,12	0,12	2	1	1
66868	M	71	BY	Diabetes mell., Leberzirrhose	Blood	12.03.2015	G	*stG245.0*	0,015	0,03	0,03	0,5	256	256	4	8	0,5
66833	M	49	SN	Empyem	Blood	12.03.2015	G	*stG643.0*	0,015	0,03	0,015	0,5	0,12	0,12	2	32	0,5
66522	F	59	NW	no data	Blood	27.02.2015	G	*stG6.1*	0,03	0,03	0,03	0,5	0,12	0,12	4	0,12	0,5
66439	F	76	RP	Erysipel, Sepsis	Blood	19.02.2015	C	*stG62647.0*	0,015	0,03	0,015	0,5	0,12	0,12	2	0,25	1
65854	M	77	RP	Sepsis, Bypassinfektion	Blood	08.01.2015	C	*stG62647.0*	0,015	0,03	0,03	0,5	0,12	0,12	2	0,5	1
65675	F	85	HE	no data	Blood	02.01.2015	G	*stG652.0*	0,015	0,03	0,015	0,5	0,12	0,12	2	0,5	2
65587	M	70	SN	Endokarditis, Sepsis	Blood	04.01.2015	C	*stG62647.0*	0,015	0,03	0,03	0,5	0,12	0,12	4	0,5	1
65525	M	63	SN	Gelenkerguss	Puncture joint	26.12.2014	G	*stG652.0*	0,015	0,03	0,03	0,5	256	0,12	4	32	1
65475	M	71		STSS	Blood	24.11.2014	G	*stG10.0*	0,015	0,03	0,03	0,5	0,12	0,12	2	32	1
65464	F	77	RP	Knie-TEP-Infektion	Puncture Kniegelenk	20.12.2014	G	*stG6.1*	0,015	0,015	0,015	0,5	0,12	0,12	2	0,5	0,5
65266	M	72	SN	no data	Puncture knee	10.12.2014	G	*stG245.0*	0,015	0,03	0,03	0,5	0,12	0,12	2	32	1
65161	F	89	NW	no data	Blood	08.12.2014	G	*stG6.3*	0,015	0,015	0,015	0,5	0,12	0,12	4	0,5	1
65042	M	56	RP	no data	Blood	14.11.2014	C	*stG62647.0*	0,015	0,03	0,015	0,5	0,12	0,12	2	0,5	1
64984	M	69	SN	no data	Blood	17.11.2014	G	*stC74a.0*	0,015	0,03	0,03	0,5	0,12	0,12	2	4	0,5
64888	M	56	RP	no data	Blood	06.11.2014	C	*stG62647.0*	0,015	0,03	0,03	0,5	0,12	0,12	4	0,5	1
64750	M	79	NW	no data	Blood	29.10.2014	G	*stG652.0*	0,015	0,03	0,03	0,5	0,12	0,12	4	32	0,5
64635	F	75	NW	no data	Blood	20.10.2014	C	*stG62647.0*	0,015	0,03	0,03	0,5	0,12	0,12	2	0,5	1
64504	n/a	71	SN	no data	Blood	04.10.2014	G	*stG10.0*	0,015	0,03	0,03	0,5	0,12	0,12	2	32	1
64494	M	76	NW	no data	Blood	04.10.2014	G	*stG485.0*	0,015	0,03	0,03	0,5	0,12	0,25	4	4	0,5
64347	M	84	NW	akute Infektion der unteren Atemwege	Blood	15.09.2014	C	*stG62647.0*	0,015	0,03	0,03	0,5	0,12	0,12	2	0,5	1
64317	M	67	NW	no data	Blood	14.09.2014	G	*stC74a.0*	0,015	0,015	0,015	0,5	0,12	0,12	2	0,5	1
64307	F	93	NW	no data	Blood	10.09.2014	C	*stG62647.0*	0,015	0,03	0,03	0,5	0,12	0,12	2	0,5	1
64299	M	50	NW	no data	Blood	14.09.2014	C	*stG62647.0*	0,015	0,03	0,03	0,5	0,12	0,12	4	0,5	1
64282	M	86	BW	Sepsis	Blood	08.09.2014	G	*stG485.0*	0,015	0,015	0,015	0,5	8	0,5	4	4	0,5
64036	M	64	HE	sept. Arthritis	Puncture	21.08.2014	G	*stG643.0*	0,015	0,03	0,015	0,5	0,12	0,12	2	32	1
63887	M	69	NW	STSS, Meningitis	CSF	17.08.2014	G	*stG10.0*	0,015	0,03	0,03	0,5	0,12	0,12	2	64	1
63859	M	72	HE	Erysipel	Blood	06.08.2014	G	*stG6792.0*	0,015	0,03	0,03	0,5	0,25	0,12	2	0,5	1
63853	F	0	SH	pneumonia, Erysipel	Blood	07.08.2014	G	*stG10.0*	0,015	0,03	0,03		0,12	0,12	2	64	1
63835	F	88	SH	Sepsis, NSTEMI	Blood	03.08.2014	C	*stG62647.0*	0,015	0,03	0,015	0,5	0,12	0,12	4	0,5	1
63826	F	44	RP	Sepsis, Axilla-Abszess mit+ beginnender nekrotisierenden Fasziitis	Blood	30.07.2014	C	*stG62647.0*	0,015	0,03	0,03	0,5	0,12	0,12	4	0,5	1
63808	F	71	SH	pneumonia, Sepsis	Blood	01.08.2014	G	*stG10.0*	0,015	0,03	0,015	0,5	0,12	0,12	2	0,5	1
63773	M	75	RP	Endokarditis	Mitralklappe	18.07.2014	G	*stG485.0*	0,015	0,015	0,015	0,5	0,12	0,12	2	64	0,5
63740	M	58	BW	no data	Blood	23.07.2014	A	*stG485.0*	0,015	0,03	0,03	0,5	0,25	0,25	2	8	2
63739	F	59	SN	Sepsis, STSS	Blood	24.07.2014	C	*stG62647.0*	0,015	0,03	0,03	0,5	0,12	0,12	2	0,25	1
63697	F	76	SL	no data	Blood	18.07.2014	G	*stG10.0*	0,015	0,03	0,015	0,5	0,12	0,12	4	64	1
63651	F	75	BY	V.a. Ischämie	Blood	30.06.2014	G	*stG2078.0*	0,015	0,03	0,015	0,5	4	0,25	4	1	0,5
63597	M	73	RP	Erysipel	Blood	30.06.2014	G	*stG6.3*	0,015	0,03	0,03	0,5	256	0,25	2	64	1
63559	M	43	RP	pneumonia	Blood	23.06.2014	G	*stG480.0*	0,015	0,03	0,03	0,5	0,12	0,12	4	1	1

63444	F	92	HE	Sepsis	Blood	09.06.2014	C	*stG62647.0*	0,015	0,03	0,03	0,5	0,12	0,12	2	0,5	1
63400	M	84	BB	no data	Blood	10.06.2014	G	*stC74a.0*	0,015	0,12	0,03	0,5	0,25	0,12	2	2	0,5
63203	F	52	RP	Sepsis	Blood	23.05.2014	G	*stC74a.0*	0,015	0,03	0,03	0,5	0,12	0,12	4	32	1
63104	M	63	NW	no data	Blood	18.05.2014	C	*stG62647.0*	0,015	0,015	0,015	0,5	0,12	0,12	2	0,25	1
62930	F	44	NW	Pericardempyem	Puncture Pericard	21.04.2014	C	*stG62647.0*	0,015	0,03	0,015	0,5	0,12	0,12	4	0,5	1
62876				no data	Blood	30.04.2014	G	*stC74a.0*	0,015	0,015	0,015	0,5	0,12	0,12	2	4	1
62617	F	89	HE	no data	Blood	03.04.2014	G	*stG245.0*	0,015	0,03	0,03	0,5	0,12	0,12	4	64	1
62435	F	71	BY	unklares Fieber	Blood	29.03.2014	C	*stG62647.0*	0,015	0,015	0,015	0,5	0,12	0,12	2	0,5	1
62340	F	57	MV	Fieber, Endometriumkarzinom	Blood	23.03.2014	C	*stG62647.0*	0,015	0,015	0,015	0,5	0,12	0,12	4	0,5	1
62183	M	61	NW	no data	Blood	17.03.2014	G	*stG485.0*	0,015	0,03	0,03	0,5	0,12	0,12	4	32	1
62164	M	50		no data	Blood	07.03.2014	G	*stG2078.0*	0,015	0,03	0,015	0,5	0,12	0,12	4	0,5	0,5
62113	M	58	BY	Meningitis, Phlegmone, Sepsis	Blood	10.03.2014	G	*stG245.0*	0,015	0,015	0,015	0,5	0,12	0,12	4	16	0,5
61834	M	73	NW	Erysipel	Blood	26.02.2014	G	*stG652.0*	0,015	0,03	0,03	0,5	0,12	0,12	4	0,5	0,5
61833	F	90	NW	Sepsis, Harwegsinfektion	Blood	24.02.2014	G	*stG10.0*	0,015	0,03	0,03	0,5	0,12	0,12	2	64	1
61724	F	79	SN	Sepsis	Blood	22.02.2014	L	*stL1376.0*	0,015	0,03	0,03	0,5	0,12	0,12	4	4	1
61703	M	62	SL	no data	Blood	18.02.2014	G	*stG485.0*	0,015	0,03	0,015	0,5	256	256	4	64	0,5
61393	M	87	HE	Sepsis	Blood	30.01.2014	C	*stG485.0*	0,015	0,015	0,015	0,5	0,12	0,12	4	0,5	1
61266	M	70	NW	Sepsis	Blood	24.01.2014	C	*stG62647.0*	0,015	0,03	0,03	0,5	4	256	4	0,5	1
60990	M	75	NW	no data	Blood	01.01.2014	C	*stG62647.0*	0,015	0,03	0,03	0,5	0,12	0,12	4	1	1
60989	F	45	NW	no data	Blood	02.01.2014	G	*stG166b.0*	0,015	0,03	0,03	0,5	0,12	0,12	4	1	1
60951	M	70	RP	no data	others Schrittmachersonde	27.12.2013	C	*stG62647.0*	0,015	0,015	0,015	0,5	0,12	0,12	4	1	1
60886	M	62	HE	others, Sarkom	Blood	26.12.2013	G	*stG166b.0*	0,015	0,03	0,015	0,5	0,12	0,12	4	0,5	0,5
60878	M	79	NW	no data	Blood	02.01.2014	C	*stG62647.0*	0,015	0,03	0,03	0,5	0,12	0,12	4	0,5	0,5
60656	M	73	HE	Sepsis	Blood	11.12.2013	G	*stG652.0*	0,015	0,03	0,03	0,5	256	256	4	32	0,5
60480	M	74	NW	no data	Blood	28.11.2013	G	*stG6.1*	0,015	0,03	0,03	0,5	0,12	0,12	4	0,5	0,5
60326	M	59	RP	Sepsis	Blood	19.11.2013	C	*stG485.0*	0,015	0,03	0,03	0,5	0,12	0,12	2	32	1
60254	F	71	RP	Erysipel	Blood	09.11.2013	C	*stG62647.0*	0,015	0,03	0,03	0,5	0,12	0,12	2	0,5	1
60198	M	77	SL		Blood	06.11.2013	G	*stG245.0*	0,015	0,03	0,03	0,5	0,12	0,12	4	64	1
60139	F	75	HE	Erysipel, Pneumonie	Blood	28.10.2013	G	*stG245.0*	0,015	0,03	0,03	0,5	0,12	0,12	2	64	1
60011	M	46	HE	unknown	Blood	19.10.2013	C	*stG62647.0*	0,015	0,03	0,03	0,5	0,12	0,12	4	0,5	1
59868	M	45	NW		Blood	08.10.2013	G	*stG6792.17*	0,015	0,03	0,03	0,5	0,25	0,12	4	0,5	1
59707	M	65	BY	Infekt bei K.-Tep	Gelenkpunktat	16.09.2013	C	*stG62647.0*	0,015	0,015	0,015	0,5	0,12	0,12	4	0,5	1
59570	F	64	HE	Erysipel, Sepsis	Blood	19.08.2013	G	*stG245.0*	0,015	0,03	0,03	0,5	4	256	2	32	1
59566	M	74	RP	Erysipel	Blood	14.08.2013	G	*stG10.0*	0,015	0,03	0,03	0,5	0,12	0,12	2	32	1
59460	M	66	NW		Blood	09.08.2013	G	*stG6792.0*	0,015	0,03	0,03	0,5	0,12	0,12	2	0,25	1
59448	F	69	HE	Sepsis	Blood	05.08.2013	G	*stG10.0*	0,015	0,03	0,03	0,5	0,12	0,12	4	0,5	1
59439	M	78	SL		Blood	02.08.2013	C	*stG62647.0*	0,015	0,03	0,03	0,5	0,12	0,12	4	0,5	2
59328	M	73	NW	Erysipel, Fasciitis necroticans	Blood	18.07.2013	C	*stC6979.0*	0,015	0,03	0,03	0,5	0,25	0,25	2	64	1
59320	M	68	RP	Erysipel	Blood	11.07.2013	C	*stG62647.0*	0,015	0,03	0,03	0,5	0,25	0,25	4	1	1
59316	M	38	NW	Sepsis	Blood	14.07.2013	C	*stG62647.0*	0,015	0,03	0,03	0,5	0,25	0,25	2	0,5	0,5
59312	F	40	HE	chronische Infektion	Gelenkpunktat	10.07.2013	C	*stG62647.0*	0,03	0,06	0,03	0,5	0,12	0,12	2	1	1
59288	F	81	BB	no data	Blood	09.07.2013	G	*stG652.0*	0,015	0,03	0,03	0,5	256	256	2	32	0,5
59287	F	82	BB	no data	Blood	07.07.2013	C	*stG62647.0*	0,015	0,03	0,03	0,5	0,12	0,12	4	0,5	1
59276	F	77	HE	no data	Blood	07.07.2013	G	*stC74a.0*	0,015	0,015	0,015	0,5	0,12	0,12	2	4	0,5
59108	M	74	RP	Erysipel	Blood	21.06.2013	C	*stG62647.0*	0,015	0,03	0,03	0,5	0,12	0,12	4	0,5	1
59072	M	72	NW	no data	Blood	23.06.2013	G	*stG485.0*	0,015	0,03	0,03	0,5	4	0,12	2	64	0,5
58946	M	62	HE	Sepsis	Blood	14.06.2013	C	*stG62647.0*	0,03	0,03	0,03	0,5	0,12	0,12	2	0,5	1
58861	M	73	SL	no data	Blood	31.05.2013	G	*stG643.0*	0,015	0,03	0,03	0,5	0,12	0,12	2	32	0,5
58860	M	69	SL	no data	Blood	28.05.2013	C	*stG62647.0*	0,015	0,015	0,015	0,5	0,12	0,12	2	0,5	1
58723	F	57	RP	sept. Arthritis	Gelenkpunktat	25.05.2013	G	*stG6792.0*	0,015	0,015	0,015	0,5	0,12	0,12	2	0,5	0,5
58592	F	58	NW	Erysipel	Blood	20.05.2013	G	*stG480.0*	0,015	0,03	0,015	0,5	0,12	0,12	4	0,5	1

58409	M	47	NW	Erysipel	Blood	06.05.2013	G	*stG22.1*	0,015	0,015	0,015	0,5	0,12	0,12	4	0,5	1
58398	F	80	NW	no data	Blood	14.05.2013	G	*stG480.0*	0,015	0,03	0,03	0,5	0,12	0,12	4	0,5	1
58366	M	33	RP	Erysipel, Sepsis	Blood	29.04.2013	C	*stG62647.0*	0,015	0,03	0,03	0,5	4	0,12	4	0,5	1
58272	M	73	HE	Erysipel, Pneumonie	Blood	28.04.2013	G	*stG480.0*	0,015	0,015	0,015	0,5	0,12	0,12	2	0,5	1
58121	n/a	57	NW	no data	Blood	23.04.2013	G	*stC74a.0*	0,015	0,03	0,03	0,5	8	0,12	4	8	1
58020	M	66	RP	Endokarditis	Blood	10.04.2013	C	*stG62647.0*	0,015	0,015	0,015	0,5	0,12	0,12	2	0,5	1
58002	F	74	HE	no data	Blood	10.04.2013	G	*stG652.0*	0,015	0,015	0,015	0,5	0,12	0,12	4	0,5	0,5
57884	F	80	NW	Gefäßpathogene Infektion	Blood	06.04.2013	G	*stC74a.0*	0,015	0,03	0,015	0,5	0,12	0,12	4	64	0,5
57255	M	48	NW	no data	Puncture knee	28.02.2013	G	*stG62647.0*	0,015	0,03	0,03	0,5	0,12	0,12	4	1	1
57112	M	83	BB	pneumonia	Blood	18.02.2013	G	*stG2078.0*	0,015	0,03	0,03	0,5	0,12	0,12	4	0,5	1
57100	M	78	RP	Fieber ohne Fokus	Blood	14.02.2013	C	*stG62647.0*	0,015	0,03	0,03	0,5	0,25	0,12	4	0,5	0,5
56735	M	78	TH	Sepsis	Blood	03.02.2013	C	*stG62647.0*	0,015	0,03	0,03	0,5	0,12	0,12	4	0,5	1
56734	F	78	TH	Sepsis	Blood	05.02.2013	G	*stC74a.0*	0,015	0,015	0,015	0,5	0,12	0,12	4	8	0,5
56204	F	80	SL	Sepsis	Blood	03.12.2012	G	*stG485.0*	0,015	0,015	0,015	0,5	0,12	0,12	4	64	0,5
56052	F	80	TH	Sepsis	Blood	28.12.2012	C	*stG62647.0*	0,015	0,03	0,03	0,5	0,25	0,25	4	1	1
55993	M	73	NW	acute exacerbation by bronchitis	Blood	23.12.2012	G	*stG6.1*	0,015	0,03	0,03	0,5	0,25	0,12	2	0,5	0,5
55928	M	84	RP	pneumonia	Blood	16.12.2012	C	*stG62647.0*	0,015	0,03	0,03	0,5	0,25	0,25	4	0,5	1
55760	M	52	SN	Fieber unklarer Genese	Blood	28.11.2012	C	*stG62647.0*	0,015	0,03	0,015	0,5	0,12	0,12	4	0,5	1
55507	M	61	RP	Erysipel	Blood	19.11.2012	C	*stC6979.0*	0,015	0,03	0,03	0,5	256	256	4	64	1
55455	M	55	BE	STSS	Blood	17.11.2012	C	*stG62647.0*	0,015	0,03	0,03	0,5	0,12	0,12	2	0,5	1
55213	F	75	SL	Sepsis	Blood	20.10.2012	G	*stG480.0*	0,015	0,03	0,03	0,5	0,12	0,12	2	0,5	1
55205	F	73	TH	Endokarditis, Sepsis	Blood	11.10.2012	G	*stG485.0*	0,015	0,03	0,015	0,5	0,12	0,12	4	0,5	8
55068	M	85	RP	Erysipel	Blood	07.10.2012	G	*stG6.1*	0,015	0,03	0,03	0,5	0,25	0,12	4	32	0,5
54959	M	71	NW	Erysipel	Blood	20.09.2012	G	*stG6.0*	0,015	0,03	0,03	0,5	0,25	0,12	4	0,5	0,5
54874	F	74	HE	Bronchitis, Asthma	Blood	10.09.2012	C	*stG6.1*	0,015	0,03	0,03	0,5	0,12	0,12	4	0,25	1
54855	F	74	HE	no data	Blood	30.08.2012	G	*stG166b.0*	0,03	0,03	0,03	0,5	0,25	0,12	4	0,5	0,5
54845	M	78	RP	Erysipel	Blood	29.08.2012	G	*stG485.0*	0,015	0,03	0,03	0,5	0,12	0,12	4	64	0,5
54842	M	65	BY	ZNS-Erkrankung	Blood	28.08.2012	A	*stG245.0*	0,015	0,03	0,03	0,5	0,12	0,12	2	64	0,5
54823	M	48	SN	Meningitis, Sepsis	Blood	27.07.2012	C	*stGM220.0*	0,015	0,03	0,03	0,5	0,25	0,12	4	2	0,5
54807	M	60	BB	no data	Blood	01.08.2012	C	*stC36.0*	0,015	0,015	0,03	0,5	0,12	0,12	4	0,5	0,5
54804	M	41	RP	Erysipel, Sepsis	Blood	18.08.2012	G	*stG485.0*	0,015	0,03	0,03	0,5	0,12	0,12	4	2	1
54803	M	58	RP	Erysipel, Pneumonie, Sepsis	Blood	17.08.2012	C	*stG62647.0*	0,015	0,03	0,03	0,5	0,12	0,12	2	0,5	1
54784	F	84	RP	pneumonia	Blood	16.08.2012	G	*stG6792.0*	0,015	0,03	0,03	0,5	0,12	0,12	4	32	1
54733	M	49	RP	Erysipel	Blood	04.08.2012	G	*stG480.0*	0,015	0,015	0,015	0,5	0,12	0,12	4	64	1
54593	M	82	BY	Phlegmone, Sepsis, STSS	Blood	23.07.2012	G	*stG245.0*	0,015	0,03	0,03	0,5	0,12	0,12	2	64	0,5
54568	M	91	NW	pneumonia	Blood	21.07.2012	A	*stG485.0*	0,015	0,015	0,015	0,5	0,25	0,12	4	8	1
54542	F	70	SN	Fieber	Blood	14.07.2012	C	*stG62647.0*	0,015	0,03	0,03	0,5	0,12	0,12	4	0,5	1
54540	F	78	SN	Erysipel	Blood	09.07.2012	G	*stG480.0*	0,015	0,015	0,015	0,5	0,12	0,12	4	0,5	1
54529	M	71	BY	Fieber	Blood	05.07.2012	G	*stG6.1*	0,015	0,03	0,03	0,5	0,12	0,12	2	0,5	0,5
54528	F	82	BY	Infektion	Blood	08.07.2012	G	*stG485.16*	0,015	0,06	0,03	0,5	0,12	0,12	4	1	1
54396	M	74	HE	no data	Blood	24.06.2012	G	*stG6792.0*	0,015	0,03	0,03	0,5	0,12	0,12	4	0,5	1
54382	M	61	BY	Fieber	Blood	20.06.2012	G	*stC74a.0*	0,015	0,03	0,03	0,5	0,12	0,12	4	4	1
54148	M	88	RP	Erysipel, Sepsis	Blood	04.06.2012	G	*stG6.1*	0,015	0,03	0,03	0,5	0,12	0,12	4	0,5	0,5
53941	M	75	TH	Sepsis	Blood	02.06.2012	G	*stG10.0*	0,015	0,03	0,03	0,5	0,12	0,12	4	32	1
53720	M	54	BY	no data	Blood	13.05.2012	G	*stG643.0*	0,015	0,03	0,03	0,5	0,25	0,12	4	0,5	1
53662	M	39	RP	sept. Arthritis	Puncture knee	13.05.2012	C	*stG62647.0*	0,015	0,03	0,03	0,5	0,25	0,25	4	0,5	1
53408	M	74	TH	Sepsis	Blood	13.04.2012	G	*stG485.0*	0,015	0,03	0,03	0,5	0,25	0,25	4	64	0,5
53406	M	72	TH	Sepsis	Blood	25.04.2012	G	*stG643.0*	0,015	0,03	0,03	0,5	0,12	0,25	4	0,5	0,5
53284	F	64	NW	sept. Arthritis	Blood	07.04.2012	C	*stG62647.0*	0,015	0,03	0,03	0,5	0,25	0,12	4	0,5	1
53258	M	72	HE	no data	Blood	14.04.2012	G	*stG485.0*	0,015	0,03	0,03	0,5	0,12	0,12	2	0,5	1
53184	M	64	HE	no data	Blood	07.04.2012	G	*stG643.0*	0,015	0,03	0,03	0,5	0,12	0,12	4	32	0,5

53166	M	70	RP	Sepsis	Blood	28.03.2012	G	*stG6.18*	0,015	0,03	0,03	0,5	0,12	0,12	4	4	0,5
52503	F	64	HE	pneumonia, Sepsis	Blood	28.02.2012	G	*stG166b.0*	0,015	0,015	0,015	0,5	0,12	0,12	2	0,5	0,5
52312	M	60	HE	no data	Blood	12.02.2012	C	*stG62647.0*	0,015	0,03	0,03	0,5	0,12	0,12	2	0,25	1
52241	F	59	TH	Sepsis	Blood	12.02.2012	G	*stG652.0*	0,015	0,03	0,03	0,5	0,25	0,12	2	16	0,5
52057	M	76	NW	no data	Blood	02.02.2012	G	*stC74a.14*	0,015	0,015	0,015	0,5	0,12*	0,12	4	4	1
52023	F	86	TH	Sepsis	Blood	01.02.2012	G	*stG643.0*	0,015	0,03	0,03	0,5	0,12*	0,12	4	16	0,5
51953	F	77	HE	no data	Blood	27.01.2012	C	*stG62647.0*	0,015	0,03	0,015	0,5	0,12*	0,12	4	0,25	1
51578	M	67	SL	no data	Blood	23.12.2011	C	*stG62647.0*	0,015	0,03	0,03	0,5	0,12*	0,12	4	0,5	0,5
51066	M	67	SN	Endokarditis, Sepsis	Blood	26.11.2011	G	*stG485.0*	0,015	0,03	0,015	0,5	0,12*	0,12	4	0,5	1
51054	F	25	SN	Bursitis	Puncture Ellenbogen	29.11.2011	C	*stG62647.0*	0,015	0,03	0,03	0,5	0,12*	0,12	4	0,5	1
50904	F	88	HE	Sepsis	Blood	17.11.2011	C	*stG643.1*	0,015	0,03	0,03	0,5	0,12*	0,12	4	0,5	1
50826	M	75	BY	no data	Blood	14.11.2011	G	*stG6.1*	0,015	0,03	0,03	0,5	0,12*	0,12	4	0,5	0,5
50747	M	82	RP	unklarer Fokus	Blood	31.10.2011	G	*stG6.1*	0,015	0,03	0,03	0,5	0,12*	0,12	4	0,25	0,5
50385	M	61	HE		Blood	08.10.2011	G	*stG6792.0*	0,015	0,03	0,03	0,5	2*	0,12	4	0,5	1
50301	M	68	HE	no data	Blood	02.10.2011	G	*stG652.0*	0,015	0,03	0,015	0,5	16*	16	4	16	0,5
50205	M	71	RP	Erysipel	Blood	27.09.2011	C	*stG62647.0*	0,015	0,03	0,015	0,5	0,12*	0,12	4	0,25	1
50115	F	96	RP	Erysipel	Blood	16.09.2011	G	*stC74a.0*	0,015	0,06	0,06	1	0,12*	0,12	8	8	0,5
50098	M	87	RP	Erysipel	Blood	12.09.2011	G	*stG2078.0*	0,015	0,03	0,03	0,5	16*	16	4	0,5	1
50085	M	66	NW	Fasciitis necroticans	Gewebe Wadenmuskel	09.09.2011	G	*stG652.0*	0,015	0,03	0,03	0,5	0,12*	0,12	4	16	0,5
50083	F	71	RP		Blood	08.09.2011	C	*stG62647.0*	0,015	0,03	0,03	0,5	0,12*	0,12	4	0,25	1
49980	M	76	HE	Erysipel linker Unterschenkel	Blood	23.08.2011	G	*stG643.0*	0,015	0,03	0,03	0,5	0,12*	0,12	4	0,5	0,5
49957	M	62	BY		Blood	11.08.2011	G	*stG2574.3*	0,015	0,03	0,03	0,5	0,12*	0,12	4	16	1
49906	M	78	TH	Sepsis	Blood	07.08.2011	C	*stG62647.0*	0,015	0,015	0,015	0,5	0,12*	0,12	4	0,25	1
49846	M	71	TH	septische Arthritis	Kniegelenkpunktat	26.07.2011	G	*stG10.0*	0,015	0,015	0,015	0,5	0,12*	0,12	4	16	0,5
49831	M	73	BY	Sepsis	Blood	15.07.2011	G	*stG485.0*	0,015	0,03	0,015	0,5	0,12*	0,12	4	0,25	1
49800			RP	no data	Blood	19.07.2011	G	*stG245.10*	0,015	0,015	0,015	0,5	0,12*	0,12	4	16	0,5
49796			RP	no data	Blood	19.07.2011	G	*stG245.10*	0,015	0,015	0,015	0,5	0,12*	0,12	4	16	0,5
49683	F	82	BY	Erysipel	Blood	28.06.2011	G	*stC74a.0*	0,015	0,03	0,015	0,5	0,12*	0,12	4	4	0,5
49568	M	41	BY	no data	Blood	08.06.2011	G	*stG2078.0*	0,015	0,03	0,03	0,5	4*	0,25	4	0,5	1
49547	M	69	SL	no data	Blood	11.06.2011	G	*stG652.0*	0,015	0,03	0,03	0,5	0,12*	0,12	4	16	0,5
49435	M	60	SN	Sepsis	Blood	24.05.2011	G	*stG6.3*	0,015	0,03	0,03	0,5	0,12*	0,12	4	0,25	1
48871	M	72	SL	Sepsis	Blood	05.04.2011	G	*stG2574.3*	0,015	0,03	0,015	0,5	0,12*	0,12	4	16	1
48860	M	81	RP	Sepsis	Blood	04.04.2011	G	*stG6792.0*	0,015	0,03	0,015	0,5	0,12*	0,12	4	0,5	1
48328	M	68	SL	Sepsis	Blood	03.03.2011	C	*stG62647.0*	0,015	0,03	0,015	0,5	0,12*	0,12	4	0,5	1
48269	M	76	HE	Endokarditis	Blood	24.02.2011	G	*stG485.0*	0,015	0,015	0,015	0,5	0,12*	0,12	4	16	0,5
48249	M	58	HE	Endokarditis	Blood	01.02.2011	G	*stG485.0*	0,015	0,03	0,015	0,5	4*	0,12	4	0,5	1
47927	M	56	HE	others	Blood	05.02.2011	C	*stG62647.0*	0,015	0,03	0,03	0,5	0,12*	0,25	4	0,5	1
47873	M	46	NW	Fasciitis necroticans, Leiste	Swab wound	04.02.2011	G	*stG485.0*	0,015	0,03	0,03	0,5	0,12*	0,12	4	2	1
47651	F	83	TH	Sepsis	Blood	09.01.2011	G	*stG6.1*	0,015	0,03	0,03	0,5	0,12*	0,12	4	0,5	0,5
47590	M	85	BY	Sepsis, STSS	Blood	15.01.2011	G	*stG643.0*	0,015	0,03	0,03	0,5	0,12*	0,12	4	16	1
47589	M	48	SN	Sepsis, Superinfiz. erythromderm. Ekzem	Blood	14.01.2011	C	*stG62647.0*	0,015	0,03	0,03	0,5	0,12*	0,12	4	0,5	1
47588	M	60	SN	Pyodermie (US), Wundheilungsstörung, chron. Verlauf	Blood	18.01.2011	G	*stG643.0*	0,015	0,015	0,015	0,5	0,12*	0,12	4	16	1
47247	M	63	BW	Sepsis, Muskelschmerzen	Blood	20.12.2010	G	*stC74a.0*	0,015	0,03	0,03	0,5	0,12*	0,12	4	16	0,5
46894	M	71	BW	Infektion Hüft-TEP	Puncture hip	24.11.2010	G	*stG6.1*	0,015	0,03	0,03	0,5	0,12*	0,12	4	0,25	0,5
46793	M	67	SN	unklares Fieber	Blood	13.11.2010	G	*stG6.1*	0,015	0,03	0,03	0,5	0,12*	0,12	4	0,25	0,5
46731	M	63	RP		Blood	07.11.2010	G	*stG485.0*	0,015	0,03	0,03	0,5	0,12*	0,12	4	4	0,5
46652	M	61	TH	Sepsis	Blood	02.11.2010	G	*stC74a.0*	0,015	0,03	0,03	0,5	0,12*	0,12	4	2	0,5
46575	M	76	TH	Sepsis	Blood	29.10.2010	C	*stG62647.0*	0,015	0,03	0,03	0,5	0,12*	0,12	4	0,25	1

46520	F	89	HE	erysipel, Sepsis, phlegmone, Pneumonie	Blood	19.10.2010	G	*stG643.0*	0,015	0,03	0,03	0,5	0,12*	0,12	4	16	1
46394	M	76	BW	Sepsis	Blood	02.10.2010	G	*stG840.0*	0,015	0,03	0,03	0,5	0,12*	0,12	4	4	1
46393	M	74	BW	Sepsis	Blood	03.10.2010	G	*stC74a.0*	0,015	0,03	0,03	0,5	0,12*	0,12	4	4	0,5
46311	F	85	SL	Sepsis	Blood	08.10.2010	G	*stG485.0*	0,015	0,03	0,03	0,5	0,12*	0,12	4	16	1
46304	F	87	RP	Erysipel, Sepsis	Blood	04.10.2010	G	*stG10.0*	0,015	0,03	0,03	0,5	0,12*	0,12	4	16	0,5
45993	F	83	SL	Sepsis	Blood	11.09.2010	G	*stG2078.0*	0,015	0,03	0,03	0,5	0,12*	0,12	4	0,5	1
45965	F	60	TH	Sepsis	Blood	08.09.2010	G	*stC74a.0*	0,015	0,03	0,03	0,5	0,12*	0,12	4	4	0,5
45912	M	0	SL	Sepsis	Blood	05.09.2010	C	*stG62647.0*	0,015	0,03	0,03	0,5	0,12*	0,12	4	0,5	1
45728	M	78	BW		Blood	14.08.2010	C	*stG62647.0*	0,015	0,03	0,015	0,5	0,12*	0,12	4	0,25	1
45715	F	72	NW		Blood	09.08.2010	G	*stG643.0*	0,015	0,015	0,015	0,5	0,12*	0,12	4	0,5	1
45670	F	84	BW	Sepsis	Blood	04.08.2010	G	*stG6.1*	0,015	0,015	0,015	0,5	0,12*	0,25	4	0,25	0,5
45617	M	69	NW	Erysipel	Blood	27.07.2010	C	*stG62647.0*	0,015	0,03	0,03	0,5	0,12*	0,12	4	0,5	1
45506	M	73	NW	Sepsis	Blood	13.07.2010	G	*stG6.1*	0,015	0,03	0,03	0,5	0,12*	0,12	4	0,25	0,5
45443	F	80	SL	Sepsis	Blood	06.07.2010	G	*stG643.0*	0,015	0,03	0,015	0,5	0,12*	0,12	4	16	1
45419	F	73	SH		Blood	27.06.2010	C	*stC6979.0*	0,015	0,03	0,03	0,5	0,12*	0,12	4	2	1
45119	M	52	HE		Blood	18.06.2010	G	*stG485.0*	0,015	0,03	0,03	0,5	0,12*	0,12	4	2	1
45118	M	59	HE		Blood	20.06.2010	C	*stG62647.0*	0,015	0,03	0,015	0,5	0,12*	0,25	4	0,5	1
45101	M	75	HE	Sepsis, STSS	Blood	10.06.2010	C	*stG62647.0*	0,015	0,03	0,03	0,5	0,12*	0,12	4	0,5	1
44766	F	66	RP	Phlegmone	Blood	13.05.2010	G	*stG6.1*	0,015	0,015	0,015	0,5	2*	0,12	4	0,25	0,5
44336	M	55	NW		Muskelgewebe	02.05.2010	G	*stG10.0*	0,015	0,03	0,03	0,5	0,12*	0,12	4	16	1
44317	M	52	BW	Erysipel, Sepsis	Blood	24.04.2010	G	*stG485.0*	0,015	0,03	0,03	0,5	0,12*	0,12	4	4	0,5
44250	M	43	HE		Blood	22.04.2010	C	*stG62647.0*	0,015	0,03	0,03	0,5	0,12*	0,12	4	0,5	1
43241	M	82	HE		Blood	11.03.2010	G	*stG652.1*	0,015	0,015	0,015	0,5	16*	16	4	16	1
43112	M	73	BW	Sepsis, Pneumonie	Blood	01.03.2010	G	*stG643.0*	0,015	0,03	0,03	0,5	0,12*	0,12	4	16	1
43107	M	76	HE		Blood	01.03.2010	G	*stC74a.0*	0,015	0,015	0,015	0,5	0,12*	0,12	4	4	0,5

Number: Sequential sample number of GNRCS; Age: Patient Age in years at the time of isolation; Federal state: German federal state code; Diagnosis: Primary diagnosis; Material: Sampling Material for microbiological examination; Isolation: Date of isolation; Lancefield: Lancefield-antigen of the corresponding SDSE isolate; EMM: Allotted *emm*-type of the corresponding SDSE isolate; Penicillin, Amoxicillin, Cefotaxime, Vancomycin, Erythromycin, Clindamycin, Chloramphenicol, Tetracyclin, Levofloxacin: Minimum inhibitory concentration of the corresponding antimicrobial substance in µg/ml determined by broth microdilution following CLSI guidelines (*MIC values determined for clarithromycin as surrogate substance)
